# CandiMeth: Powerful yet simple visualization and quantification of DNA methylation at candidate genes

**DOI:** 10.1093/gigascience/giaa066

**Published:** 2020-06-22

**Authors:** Sara-Jayne Thursby, Darin K Lobo, Kristina Pentieva, Shu-Dong Zhang, Rachelle E Irwin, Colum P Walsh

**Affiliations:** Genomic Medicine Research Group, School of Biomedical Sciences, Ulster University, 1 Cromore Road, Coleraine, BT52 1SA, UK; Genomic Medicine Research Group, School of Biomedical Sciences, Ulster University, 1 Cromore Road, Coleraine, BT52 1SA, UK; Present address: Republic Polytechnic, 9 Woodlands Avenue 9, Singapore 738964; Nutrition Innovation Centre for Food & Health (NICHE), School of Biomedical Sciences, Ulster University, 1 Cromore Road, Coleraine, BT52 1SA, UK; Stratified Medicine Research Groups, School of Biomedical Sciences, Ulster University, 1 Cromore Road, Coleraine, BT52 1SA, UK; Stratified Medicine Research Groups, School of Biomedical Sciences, Ulster University, 1 Cromore Road, Coleraine, BT52 1SA, UK; Genomic Medicine Research Group, School of Biomedical Sciences, Ulster University, 1 Cromore Road, Coleraine, BT52 1SA, UK

**Keywords:** galaxy, methylation, workflow, DNA methylation, arrays, epigenetics, EWAS

## Abstract

**Background:**

DNA methylation microarrays are widely used in clinical epigenetics and are often processed using R packages such as ChAMP or RnBeads by trained bioinformaticians. However, looking at specific genes requires bespoke coding for which wet-lab biologists or clinicians are not trained. This leads to high demands on bioinformaticians, who may lack insight into the specific biological problem. To bridge this gap, we developed a tool for mapping and quantification of methylation differences at candidate genomic features of interest, without using coding.

**Findings:**

We generated the workflow "CandiMeth" (Candidate Methylation) in the web-based environment Galaxy. CandiMeth takes as input any table listing differences in methylation generated by either ChAMP or RnBeads and maps these to the human genome. A simple interface then allows the user to query the data using lists of gene names. CandiMeth generates (i) tracks in the popular UCSC Genome Browser with an intuitive visual indicator of where differences in methylation occur between samples or groups of samples and (ii) tables containing quantitative data on the candidate regions, allowing interpretation of significance. In addition to genes and promoters, CandiMeth can analyse methylation differences at long and short interspersed nuclear elements. Cross-comparison to other open-resource genomic data at UCSC facilitates interpretation of the biological significance of the data and the design of wet-lab assays to further explore methylation changes and their consequences for the candidate genes.

**Conclusions:**

CandiMeth (RRID:SCR_017974; Biotools: CandiMeth) allows rapid, quantitative analysis of methylation at user-specified features without the need for coding and is freely available at https://github.com/sjthursby/CandiMeth.

## Introduction

Epigenetics can be defined as stable, and most often heritable, changes to the chromatin that do not alter the DNA sequence itself but still affect gene expression and/or are required to maintain genomic stability [1]. These modifications consist of reversible marks such as cytosine DNA methylation or histone modifications, each critical to gene expression regulation, imprinting, X-inactivation, and many other processes from mammalian gestation to later life [1].

Cytosine DNA methylation is the most common and thoroughly investigated of these epigenetic alterations. It is characterized by the addition of a methyl group to a cytosine residue, many of which are located within so-called CpG islands (CGI) close to gene promoters [2]. High levels of DNA methylation at promoters aid in the stable long-term repression of the cognizant genes, such as can be seen on the inactive X chromosome in mammals [3]. Methylation at control elements such as insulators or enhancers can also help regulate regional gene expression, with multiple examples being seen among imprinted genes [4] or gene clusters such as the protocadherins [5]. High levels of methylation are seen on selfish DNA elements such as endogenous retroviruses, where they play an important role in their suppression [6] as well as at inert regions of the genome such as pericentromeric repeats [7]. More recently, methylation through the body of the gene has been recognized as contributing to maintaining gene transcription levels at highly expressed genes [8, 9]. As well as showing such developmental programming, DNA methylation is susceptible to environmental influence, with inputs such as diet [10, 11] and exposure to pollutants such as cigarette smoke [12] having clear and reproducible effects on methylation levels, sparking great interest in analysis at a population level, particularly in humans [13].

Advances in sequencing technology have allowed us to quantify and analyse methylation via whole-genome bisulphite sequencing at ∼28 million CpG resolution [14]. While this technique remains the gold standard for whole-genome methylation assessment, it can be very expensive, and when there are hundreds of samples to be tested and analysed prohibitively so; quantifying small differences reproducibly between multiple samples is also challenging. An alternative technology known as a microarray, which predates the era of whole-genome bisulphite sequencing, is often a popular solution for such cases, where a lower CpG resolution is satisfactory but where greater intersample reproducibility is required [15]. A popular choice here is the Illumina Infinium Methylation BeadChip array [15], which currently covers 850,000 CpG sites across the human genome, including 99% of RefSeq genes and large numbers of enhancers and other features. This can help elucidate the effects of an intervention across hundreds of samples in a cost-effective manner. There are many packages across multiple computational languages to analyse the outputs from these arrays such as RnBeads [16,] or ChAMP [17], but these pipelines operate in the statistical programming environment R and require some coding. Additionally, the output file formats can be overwhelming and difficult to investigate further without experience in data analytics and bioinformatics. This situation is exacerbated by the typically higher number of samples in epidemiological or intervention studies where such arrays are commonly used.

To help solve this predicament, we developed a Galaxy workflow known as CandiMeth, which takes the main output from such methylation analysis pipelines and pairs this with a list of features that the user may wish to investigate. The workflow first generates tracks showing both absolute methylation levels in samples and differences in methylation between samples. These can be viewed via the University of California Santa Cruz (UCSC) genome browser and overlaid with other available tracks such as CpG island, enhancers, chromatin immunoprecipitation (ChIP) data, and so forth to allow data exploration and more intuitive analysis. This also facilitates the design of assays to cover specific CGs using BLAT. The workflow can then help confirm any patterns observed by quantifying data across the identified regions or features, e.g., methylation differences at specific sets of genes between cases and controls. It also has a bespoke analysis allowing estimation of methylation differences at repetitive sequences by leveraging the RepeatMasker tracks at UCSC. The workflow removes the need for further analysis in R and increases reproducibility by using an automated process, but in a more user-friendly manner.

## Methods

CandiMeth (CandiMeth, RRID:SCR_017974) (Biotools: CandiMeth) is designed to work downstream of DNA methylation analysis pipelines in R. It was developed initially using RnBeads as reference but has been subsequently successfully run with ChAMP and other packages (see below). ChAMP (ChAMP, RRID:SCR_012891) [18] and RnBeads (RnBeads, RRID:SCR_010958) [15, 18] are end-to-end pipelines in R that can take raw data files such as IDATs and bam files from microarray readers or sequencers and process these to allow data exploration, visualization, and comparison. For array data, which is the main area where CandiMeth addresses an unmet need, IDAT files containing raw values for the red and green channels for each of ∼850,000 probes are exported from the microarray reader. RnBeads/ChAMP can perform quality control, remove probes with low signal or overlapping with single-nucleotide polymorphisms (SNPs), and provide a cleaned dataset giving absolute levels of methylation as β or *M* values. The packages can also facilitate exploratory visualization through principal component analysis or similar and allow grouping of data prior to looking for differential methylation. Probes showing substantial differences in methylation (Δβ) can be identified and then ranked on the basis of a variety of parameters, including probability of occurrence (*P*-value), Δβ, false discovery rate (FDR), or a combination of several of these. The packages can look for enriched gene sets using gene ontologies/GSEA [19] and visualize differences for annotated categories of array probe such as promoter and gene body.

While packages for array analysis provide genome-level data such as whether promoters in general are losing or gaining methylation, querying specific gene sets that might give more biological insight cannot be easily done in this or other R packages with similar functionality without extracting the processed dataset and writing bespoke code. Visualization of the data against the genome map is also of great attraction for the wet-lab biologist but is also not easily done within these packages. While RnBeads can map methylation values to the genome as customized tracks, this can only be carried out if a local instance is installed on the user's server, which requires substantial investment for set-up and maintenance. ChAMP does not currently provide tracks at all, to our knowledge. Typically, many biologists have specific genes that are of interest to them, or they may want to examine the area in which top sites are located and determine whether adjacent probes are also losing or gaining methylation. A ready way of assessing the degree to which methylation is changing across a particular region and the exact location of the probes also greatly facilitates the design of gene-specific assays such as primer sets for pyrosequencing or clonal analysis. It is also generally of interest to try and leverage the enormous pool of publicly available data accessible through UCSC Genome Browser tracks to explore possible novel correlations between methylation changes in a particular dataset and other genome characteristics such as replication timing, histone modifications, or similar.

We therefore wished to develop a user-friendly non–computationally intensive method of candidate feature investigation that avoided the command line but was more powerful than browser-only interfaces. To this end we chose the Galaxy (Galaxy, RRID:SCR_006281) platform [20], which is a free open-source environment for user-friendly and reproducible bioinformatics [21]. It provides a variety of data manipulation and analysis tools via a web interface with no prior installation or dependency packages required, with results stored within the Galaxy infrastructure and every action producing a new history entry so the original data are never compromised via destructive edits. Galaxy also allows users to aggregate analysis steps into repeatable pipelines called workflows, which can be easily shared, along with the histories, via URL or username. These can allow biologists with little bioinformatics experience to conduct complex analyses on their own data within a system that has a low maintenance requirement and with little worry over data storage or data corruption. Moreover, workflows can be published to a repository such as GitHub (RRID:SCR_002630) or MyExperiment (RRID:SCR_001795) [22] or within a scientific journal—further encouraging open data science and reproducibility. Galaxy also provides many plugins such as interactive visualization software to view results, the option to export results to genome browsers, and the option to configure tools, or indeed an entire Galaxy instance, to the desired end-user needs.

### Overview of workflow

**Figure 1:**
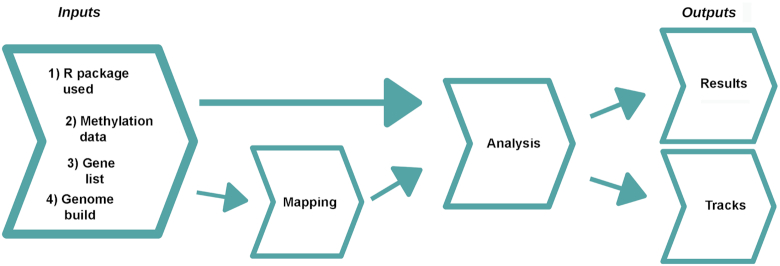
Overview of CandiMeth workflow. When the CandiMeth workflow is started the user needs to specify as Inputs (*left*): (i) the type of R package used; (ii) the methylation data, normally in the form of a differential methylation table generated by the package; (iii) a list of genes to be analysed; and (iv) a human genome build to match the data to. The methylation data are then mapped (*centre lef*t) to the genome and sites overlapping features of interest analysed (*centre right*). The data are then output quantitatively as Results and visually as Tracks (*right*).

The main process undertaken by CandiMeth is to take as input the methylation data from an R pipeline such as RnBeads or ChAMP and (i) visualize the data as tracks in the UCSC Genome Browser and (ii) analyse the methylation differences relative to genomic features specified by the user. The workflow comprises 3 main steps: Inputs, Feature Mapping, and Analysis (Fig. 1). There are also 4 items required at input stage: the user must (i) indicate the R package used with the keywords “RnBeads,” “ChAMP,” or “Custom,” then supply (ii) the methylation data, (iii) a list of the genes of interest, and (iv) specify the human genome build to be used, e.g., hg19. The basic workflow for CandiMeth is that the genes of interest are mapped to the reference genome and then cross-referenced with the input methylation data to get feature-specific statistics. The workflow can currently look at either the promoters (−500 to +1 bp relative to transcription start site; suffix “_P” on results) or gene bodies (the transcription unit; “_GB”), or both parts of the gene together (“_all”). We have found this to be a particularly useful split because the current consensus is that promoters and gene bodies can show opposite methylation patterns, with methylation at the promoter largely associated with repression, whereas gene body methylation instead is a feature of transcribed genes. Outputs are then grouped in the history into 2 types, Results or Tracks (Fig. 1). The methylation data from the R packages are output as a standard differential methylation table as routinely generated, and either a single table comparing 2 groups, or several tables can be processed at once as inputs, e.g., comparing different experimental conditions with the control. Each comparison will result in a separate table and tracks, grouped together and given a condition-specific identifier to avoid confusion. The CandiMeth workflow, together with the example datasets used and a step-by-step tutorial, are available on GitHub [23].CandiMeth is optimized to work on the latest version of Galaxy (19.0) through the Galaxy website [20, 24], thus making it platform-independent. For users who have their own instance of Galaxy, the workflow can be downloaded and imported via a link on the GitHub page, where a .yaml file is also available.

### Example outputs

**Figure 2:**
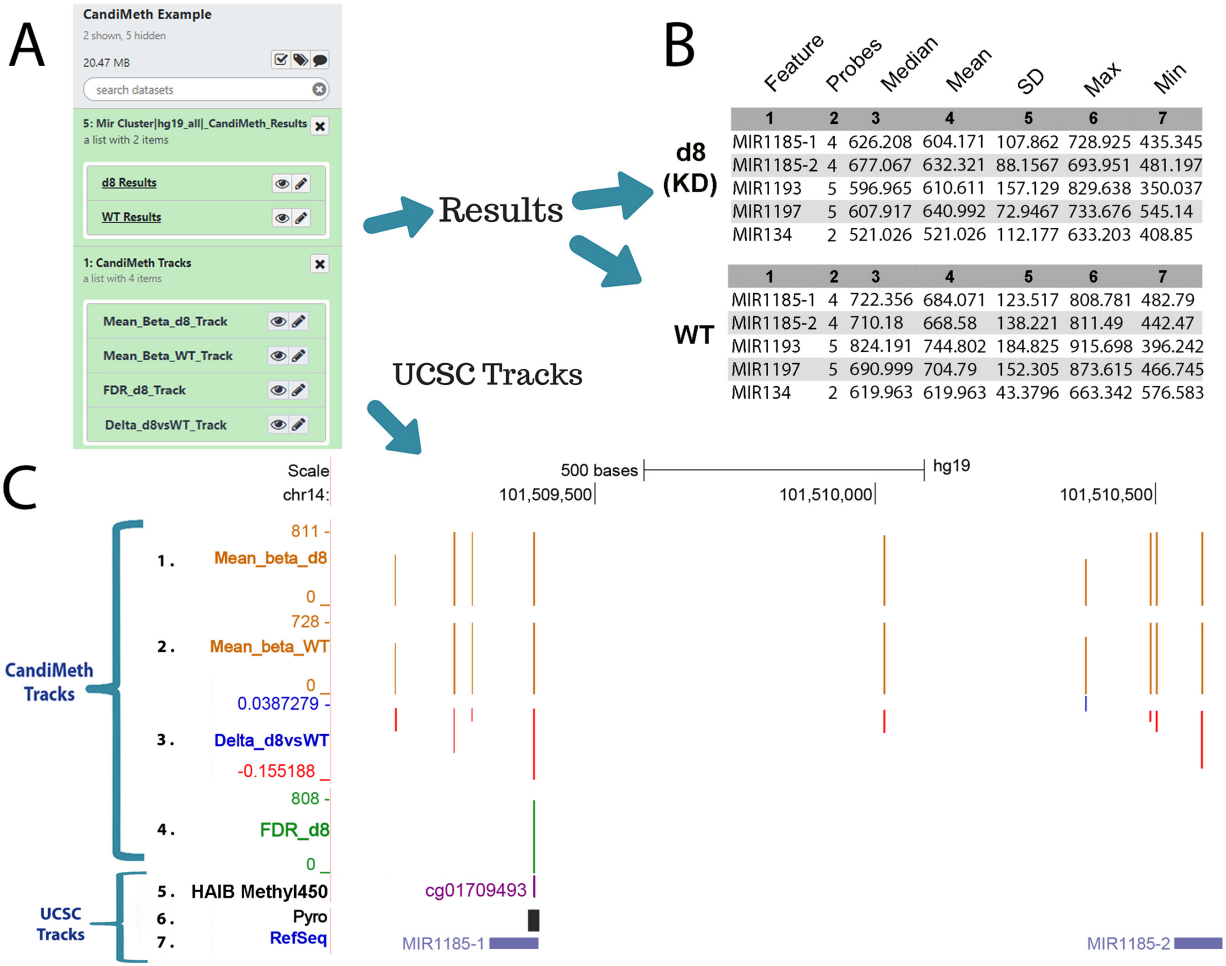
Case Study 1: analysing new genes in a published dataset. Our previously published dataset GSE90012 using RnBeads to compare methylation levels in cells deficient in DNA methyltransferase 1 (d8 KD) with wild type (WT) was reanalysed for methylation levels at microRNA (*MIR*) using CandiMeth. (**A**) The workflow generated 2 grouped sets of outputs (white boxes at left) on completion, “Mir Cluster | hg_19_all | CandiMeth Results” containing links to the tabular quantitative data and “CandiMeth Tracks” with links to the tracks on UCSC (**B**) CandiMeth Results box expanded: a separate dataset for each cell line is generated showing the list of candidates, probe coverage, median, and a variety of other statistics for each gene analysed (top 5 rows only shown). (**C**) CandiMeth Tracks: UCSC Genome Browser view, accessible via the eye symbols on the Galaxy history shown in (A): (From the top down) Scale bar, size of region in kilobases of DNA; chr1, chromosome number and exact coordinates from the hg19 genome build. (1–4) CandiMeth tracks: (1) Mean_beta_d8, absolute methylation track reflecting array output going from 1, no methylation, to 1,000, fully methylated, e.g., 811 = 81.1%, maximum and minimum indicated at left; (2) Mean_beta_WT, absolute methylation in WT; (3) delta_d8vsWT, a differential methylation track showing proportional change going from −1.0 (100% loss, red) to +1.0 (100% gain, blue), e.g., −0.155 = loss of 15.5% compared to WT; (4) FDR_D8, a significance score track showing only those sites whose differential methylation meets the cut-off criterion of a 0.05 false discovery rate. (5–7) Examples of some of the tracks available through the UCSC Genome Browser, which can be aligned and directly compared to CandiMeth tracks: (5) HAIB Methyl450, data on comparative methylation from ENCODE projects; (6) Pyro, the BLAT tool in UCSC, which can be used to find primers for pyroassays to cover 1 or multiple CG; (7) RefSeq track, showing the location of the top 2 MIR from (B).

To illustrate the type of analysis that can be done, Fig. 2 shows outputs from 1 of the example dataset runs. Here we used as input 1 of our previously published differential methylation tables generated by RnBeads (NCBI Gene Expression Omnibus [GEO] identifier GSE90012; the table is also given as Suppl. Table 1) [25]. The experiment compared wild-type hTERT1604 human fibroblast cells (WT) and a clonal derivative with a stable knockdown (KD) of the maintenance DNA methyltransferase DNMT1 (d8 KD), which gave large alterations in DNA methylation levels, very suitable for the purpose of illustration here. The second item needed for CandiMeth, namely, features of interest, was in this case a set of microRNA (MIR) genes not analysed in the original article, which was input here simply as a list of names (given in Supp. File 2). CandiMeth first mapped the MIR locations to the human genome (in this case hg19), then analysed the co-occurrence of probes at these locations. The results appeared in Galaxy as 2 grouped sets of datasets (Fig.   2A): “Mir Cluster | hg19 all | CandiMeth Results” and “CandiMeth Tracks.”

The Results set contained an output table for each condition, namely, KD (d8) and WT cells (Fig. 2B, first 5 rows of each shown). Each table consisted of 7 numbered columns. It should be noted here that methylation values from the array are expressed as a number from 1 (no methylation) to 1,000 (fully or 100% methylated) to facilitate visualization. The numbered columns correspond to (1) Feature, the candidate region of interest, in this case each of the *MIR* in the initial list; (2) Probes, the number of array probes that are found in the specified feature; (3) Median, the methylation value that is the median of all probes mapping to that feature, e.g., 626.208/1,000 is the median of all probes at *MIR1185–1*, or 62.6% methylated; (4) Mean, the mean methylation value across all probes; (5) SD, the standard deviation; (6) Max, the maximum probe value seen in the feature; and (7) Min, the minimum probe value (Fig. 2B). It can be seen that methylation values are much lower in the DNA methyltransferase-depleted cells (d8) for each miR compared to the parental or WT cells, e.g., *MIR1185–1* 62.6% median methylation in d8 vs 72.2% in KD. It can be seen that, while usually in reasonable agreement, in some cases the median and mean vary substantially, and having data on the numbers of probes can be useful for deciding confidence in the results and on any threshold to be applied.

In the Tracks folder CandiMeth also generated 4 tracks on the UCSC Genome Browser (Fig. 2C, 1–4), which can be visualized by clicking on the eye icon on the Galaxy datasets under CandiMeth Tracks in Fig. 2A (clicking on each track overlaid it on the previous one to generate the cumulative view shown).

Tracks 1 and 2 are absolute methylation (raw β) tracks, denoted as “Mean_beta” in CandiMeth outputs. These show the methylation per probe for all probes in the differential methylation table that passed quality control and other screening steps in RnBeads, and not just the feature-specific (here *MIR*) probes, as we have found that the genomic methylation context is very valuable to consider when looking at features. In other words, even if Promoters is selected at input, the tracks will show all probes, including those in the gene body and other regions. Track 1 is the DNMT1-depleted cell line (“Mean_beta_d8”) data, and Track 2 is from the WT cells (“Mean_beta_WT”).

Track 3 is the Δβ track (“Delta_d8vsWT”) showing the difference between methyltransferase-deficient and WT cells. These are BedGraph files like Tracks 1 and 2, but because methylation can be higher or lower in 1 sample versus another, the visualization is different from the absolute methylation tracks. Instead, gains in methylation in the experimental condition are shown as blue columns above the zero (no change) line, and losses are shown as red columns below the line, with a change of +1 being 100% increase and −1 being −100%, i.e., an array probe going from 100% methylated to 0% methylated. The Delta track also allows the user to see how many array probes in a region are showing large differences in methylation and whether a differentially methylated region (DMR) identified by RnBeads extends farther than originally estimated [26]. Note that this track shows all differences in methylation, however small: the FDR-corrected probes are shown in the next track.

Last, an FDR-corrected track (“FDR_D8,” Track 4) was also produced: this only showed information for those probes where the R package has assessed the FDR to be <0.05 because this is a statistical cut-off implemented by many array users. This is an excellent method for visualizing only CG that have high-confidence differences in methylation between samples. Here, only a single probe passed the FDR threshold and is shown: the absolute methylation level at the probe is given because *P*-values would not scale correctly.

One of the most powerful features of using this approach is that data can easily and more intuitively be compared to other UCSC tracks (Fig. 2C, 5–7). The specific CpG site can be identified in UCSC, e.g., by right-clicking on the column on the track, or by typing the CG identity into the UCSC browser search window, which will then pull out a track with the site highlighted, in this case the ENCODE project's HAIB Methyl450 (Fig. 2C, Track 5). A particularly useful tool in this context is UCSC's BLAT, which can be used to help ensure that primers designed to verify methylation differences at specific regions of interest by pyrosequencing or similar do indeed overlap the crucial sites (Fig. 2C Track 6, Pyro), in this case the FDR-significant site. Off-the-rack assays for each CG on the EPIC array can also now be purchased commercially. Other UCSC tracks shown in Fig. 2C include the RefSeq track (Track 7), invaluable for identifying well-curated genes rather than predicted or rare products. These tracks were all overlaid on the CandiMeth tracks, allowing the user to see whether methylation changes were located in or near any of these features. These are examples only; any track available through UCSC or that can be called through Galaxy can potentially be aligned with the CandiMeth tracks.

### Data preparation and inputs

**Figure 3:**
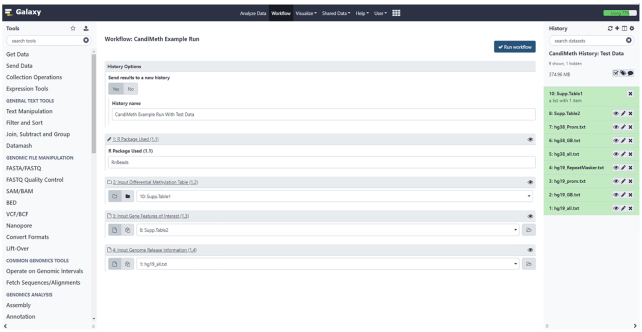
User Interface for the workflow. Screenshot of the workflow start window (*middle pane*) that appears on right-clicking >CandiMeth>Run. The right-hand side shows the CandiMeth starting history, where preloaded data used with the workflow can be found, together with any user-uploaded datasets. Galaxy tools (*left*) are not used. For the workflow the user chooses whether to save results to a new history (recommended), then specifies (1) which R package was used to pre-process the data, e.g., RnBeads; (2) the dataset collection table of pre-processed data—available sets will appear in the drop-down menu; (3) a list of the genes/other features of interest to analyse; and (4) the reference genome to be mapped to, e.g., hg19. Once all 4 have been decided, the user clicks on the blue “Run workflow” button at top right to initiate a run.

A complete User Guide document with step-by-step tutorials is available [23]; here we describe more general features of the workflow. As indicated, CandiMeth runs in the Galaxy environment: users must first create an account and copy the CandiMeth test history and workflows to their account, as explained in the Guide. Once these simple steps have been carried out the first time, they do not need to be repeated. When CandiMeth is being run, the initial window will look as shown in Fig. 3: the workflow occupies the central window, while the example data and datasets required for the workflow are in the History window at right; the left window Tools will not be used. Upon initialization, the workflow window will look as shown, with 1 Yes/No choice and 4 fields (numbered 1–4) to fill in. We recommend saving the outputs of CandiMeth to a new history when initiating the pipeline. This will (i) make it possible to continue working on other tasks while CandiMeth is running in the background—the workflow can take a while to run depending on server usage and (ii) segregate the current job from the reference datasets in the CandiMeth initial history, which avoids cluttering the initial history or causing problems if a particular run fails and generates incompletely processed datasets. The 4 fields are the 4 forms of inputs required, as indicated in the example above and dealt with below.

#### Input Type 1: R package used

CandiMeth works downstream of R-based packages that are designed to process epigenome-wide datasets. The 2 most popular packages (by Bioconductor download) ChAMP and RnBeads both automatically generate tabular data outputs that are suitable as input for CandiMeth without further processing, but the tables are in slightly different formats. Therefore, CandiMeth users should select either “RnBeads” or “ChAMP” when asked which R package was used. CandiMeth also supports other packages via a “Custom” keyword.

#### Input Type 2: Differential methylation table

The user needs to identify the location of, or upload directly, a copy of the output table from the R package containing the differential methylation data. For RnBeads this can be found via the html interface by opening “differential_methylation_html” and choosing the desired comparison table. Once uploaded, the differential methylation table must be converted to a dataset collection through a 1-step operation (see the User Guide [23, 27]), which allows all the data from the table to be processed at once. An example table is available in dataset collection format in the CandiMeth default history; the raw table itself is also available as Supp. Table 1. In addition, an example ChAMP output is also available as Suppl. Table 5 in the CandiMeth History.

If the custom option is chosen at Input 1 above, the user can input a data frame of any origin as long as it follows the default CandiMeth format, namely: Chromosome; Start; cgid; mean.X; mean.Y; the difference between the 2 groups; and the FDR-corrected *P*-value (where X and Y equal the names of the experimental and control groups, respectively). Data frames can also be rearranged in Galaxy using the text manipulation tools “cut” and “join” within the Galaxy tool panel to produce an acceptable input table. We hope to extend the number of preformatted options beyond RnBeads and ChAMP to reduce the need for custom inputs in future.

#### Input Type 3: Gene features of interest

Here the user can choose which features they want to investigate. This can be done in a customized fashion, but commonly biologists initially want to see how much methylation is present across well-defined genomic features such as genes. This can easily be done in CandiMeth by following the commands >Get Data >upload File >paste/Fetch and then typing the official gene names, 1 per line, into the window that opens there (see Step-by-Step Guide). Alternatively, they can be uploaded as a list in a tab-delimited file format at this step. To facilitate initial trials, the MIR gene names used above have been preloaded into the default CandiMeth history for use and are also supplied as Supp. Table 2. The features associated with the gene names are then mapped to the genome using the genomic data discussed next.

#### Input Type 4: Genome information

An important part of the CandiMeth workflow is the parsed human genome information used to assign array probes to various genomic features. Example human genome build information used for the mapping part of the CandiMeth pipeline can be found within the CandiMeth history (right-hand pane in Fig. 3). The data provided here cover 2 genome assemblies, hg19/hg38, and will aid the mapping of candidate features to promoters, whole gene body region, or both (hg19_all option) as defined by RefSeq [28].

Using CandiMeth, users can query RefSeq-defined genes or repeats to obtain the same types of information as can be obtained by analysis in an R package. One advantage here however is that the simultaneous visualization allows the user to inspect the match between probe location and gene structure for candidate regions of interest: e.g., the initial screen may indicate changes in promoter methylation from the manifest-defined promoter, when inspection shows that all of the probes lie in the first exon of a single-exon gene and therefore are in fact gene body, the discrepancy being due to the definition of promoter in the manifest. CandiMeth allows the user to refine or alter the promoter definition to exclude bases downstream of the transcriptional start site, for example, and re-evaluate. An approximation of promoter areas of these RefSeq genes was generated for the example data analysis and was defined as the region from 500 bp upstream to the first base (−500→ +1 bp) and is available in the CandiMeth history [29] mentioned above. Similarly, probes were also parsed into gene body and repeat categories for CandiMeth to facilitate user analysis of effects over these types of genomic intervals for their candidate genes of interest.

### Processing steps

**Figure 4:**
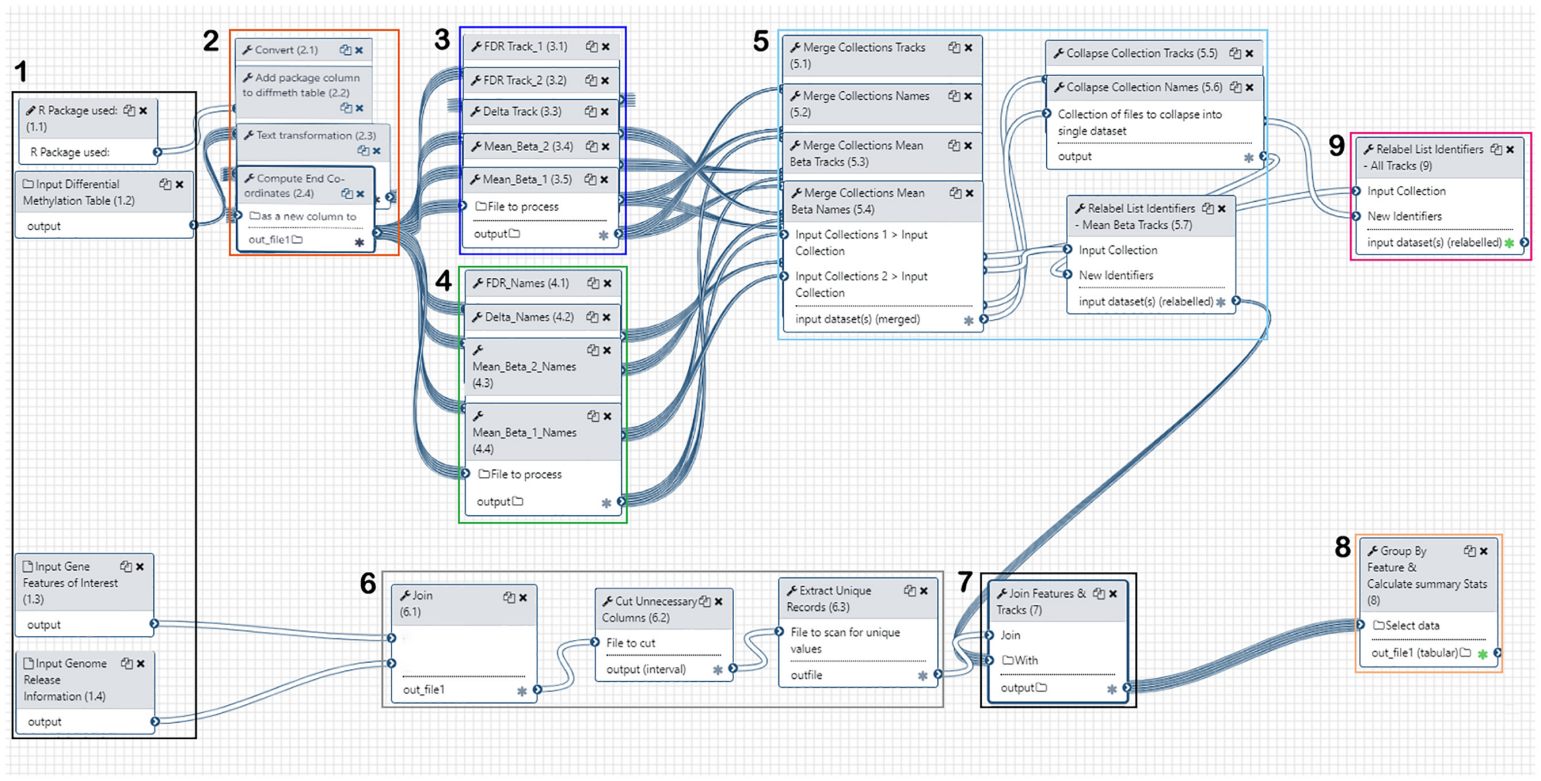
Galaxy Workflow Editor View of CandiMeth. Detailed view of the workflow using the editing tool in Galaxy. Steps in the workflow have been grouped for clarity. (1) Inputs: here the user indicates which R package was used to analyse their array using the keywords “RnBeads,” “ChAMP,” or “Custom” (1.1), identifies the differential methylation table resulting from this R package (1.2) and the genomic features that they wish to analyse (1.3), and specifies the desired genome build (1.4). (2) Standardizing the input data: Using the R package information in 1.1 and the differential methylation table in 1.2, CandiMeth generates a table showing the chromosome location, start, end, mean methylation in the control and experimental groups, the difference between these groups, and the FDR-adjusted *P*-value. (3) Track generation: maps the data on absolute as well as differential methylation from the table to the genome build. (4) Track naming: generates unambiguous labels for each type of track. (5) Merging of tracks and names: this ensures logical labelling and grouping of tracks. (6) Feature mapping: this maps the specific features to the same genome build. (7) Compilation of feature methylation: this parses the data in the tracks to only examine the features of interest. (8) Output Tables: these contain summary statistics on the features of interest and are 1 major output. (9) Output Tracks: the user can also see the mapping on which the summary statistics are based, which allows them to see areas adjacent to the features of interest, and overlay other UCSC tracks, as well as use tools such as BLAT.

Fig. 4 shows a workflow editor view of CandiMeth: different sections have been numbered for ease of reference here.

1. Inputs: Inputs are indicated at left; R package used to generate the table (1.1), differential methylation table (1.2), features of interest (1.3), and parsed genome information specific to that type of interval, e.g., promoters (1.4). Once the 4 input types have been decided (see aforementioned examples) the workflow proceeds as follows.
2. Generation of a standardized data frame between RnBeads and ChAMP: First the CSV file output from the R package is processed by converting the delimiters used into tabs (2.1), then the keyword identifier for that package (either “RnBeads,” “ChAMP,” or “Custom”) added to the differential methylation table (2.2) to form an extra column. A table is then output showing the chr, start, cgid, mean methylation between control and experimental groups, the difference between these experimental groups, and FDR-corrected *P*-value (2.3). Subsequently, the end coordinates for each cg site are calculated and added to this table (2.4), so the data can be configured to run on UCSC Genome Browser at a later stage in the workflow.
3,4. Track generation and naming: Differential Table inputs from RnBeads (1.2) are converted into a variety of tracks compatible with UCSC Genome Browser. These include 2 absolute methylation tracks (3.4, 3.5) in this case, 1 FDR track showing only FDR significant sites (3.1), and 1 Δβ track (3.3) showing the difference in β-value between the 2 absolute methylation tracks. Track and results names (4.1–4.4) are also generated from the differential table inputs: this is an important step because both absolute methylation data for individual samples and a number of types of comparison data must be separated and given logical and intuitive names to allow easy identification among the multiple output datasets. The workflow uses a number of pre-existing tools available in Galaxy to carry out these steps (Table 1).
5. Merging of tracks and names: Following track creation (3), the resultant tracks and their names are merged into separate dataset collections (5.1–5–4) and then collapsed into singular dataset collections (5.5, 5.6), one for all comparative tracks (5.5), one for all comparative track names, and one for all absolute methylation (mean β) tracks (5.4) with their associated names (5.7). The mean β tracks will be used for feature investigation later in the workflow. The results here are compilations containing information on methylation at each probe across the genome in each sample, or the differences in methylation at specific probes between pairs of samples.
6. Feature mapping: Features of interest (1.3) input by the user such as a particular set of genes are joined (6.1) to the specified genome release information (1.4) using the Paste tool. The gene features of interest are overlapped with the genome release information to obtain the desired genome intervals using AWK (6.2). Any repeated columns or rows that are no longer required are discarded and unique records extracted (6.3). The output here is a set of genomic coordinates matching only the specific features of interest, e.g., a specific set of genes.
7. Compilation of methylation data for features: The dataset collection containing now correctly named absolute methylation tracks (5.7) is now joined with the mapped features of interest (7). This allows the generation of feature-specific statistics.
8,9 Outputs: Feature-specific statistics such as mean methylation over all probes in each feature, median, maximum, etc. (see below), are tabulated and form 1 major output (8). The comparative tracks (generated in 3) are also given unambiguous final names, collated, and output as a dataset collection called “CandiMeth Tracks” (9, with green stars marking final output states).

**Table 1:** List of Galaxy tools used

Tool name	Tool ID	Version	CandiMeth step	Reference
Convert delimiters to TAB	convert_characters	1.0.0	2.1	[30]
Add column to an existing dataset	add_value	1.0.0	2.2	[31]
Text transformation with SED	tp_sed_tool	1.1.1	2.3	[32]
Compute an expression on every row	column_maker	1.2.0	2.4	[33]
Merge collections into single list of datasets	__MERGE_COLLECTIONS__	1.0.0	5	[34]
Relabel list identifiers from contents of a file	__RELABEL_FROM_FILE__	1.0.0	5.7/9	[35]
Collapse Collection into single dataset in order of collection	collapse_dataset	4.1.0	5.5/5.6	[36]
Paste 2 files side by side	Paste1	1.0.0	6.1	[37]
Text reformatting using AWK	tp_awk_tool	1.1.1	6.2	[38]
Unique occurences of each record	tp_sorted_uniq	1.1.0	6.3	[39]
Join the intervals of 2 datasets side by side	tp_easyjoin_tool	1.0.0	7	[40]
Group data by a column and perform aggregate operations on other columns	Grouping1	2.1.4	8	[41]

### Output files

The CandiMeth workflow produced as indicated above under Example outputs 2 main types of output files:

#### Tables

Results tables all follow the same layout: feature name, probe coverage, median methylation, mean methylation, standard deviation, maximum, and minimum. A partial example of a tabular output for the set of miRs used in the example above is shown in Fig. 2B (first 5 lines) and given in full in Suppl. Table 3. Methylation values for the features can then be plotted within Galaxy via their integrated visualization software or the Table can be exported and downloaded then plotted within the user's preferred visualization software such as Prism or Excel as desired.

#### Tracks

CandiMeth produced 4 different tracks from the differential methylation table input in the first step, of 3 different kinds (absolute methylation, relative differences in methylation [Δβ], and FDR-significant methylation difference), as shown in the example above for a cell line system.

## Findings

The utility of the CandiMeth workflow may be best illustrated by some case studies.

### Case Study 1: Application to array results from model systems

One straightforward use of CandiMeth that has found common use in our laboratory and among collaborators is to test a specific gene set, as illustrated by the *MIR* example above (Fig. 2). To do this, the user only has to specify a list of the names of the genes they are interested in, together with the genome release, then upload a table containing differential methylation data. This can either be one generated by the bioinformatics team in-house; one that was supplied, typically when array services are brought in; or one that was generated from publicly available array data such as our dataset GSE90012 described previously [42] and used above.

### Case Study 2: Application to EWAS study outputs

**Figure 5:**
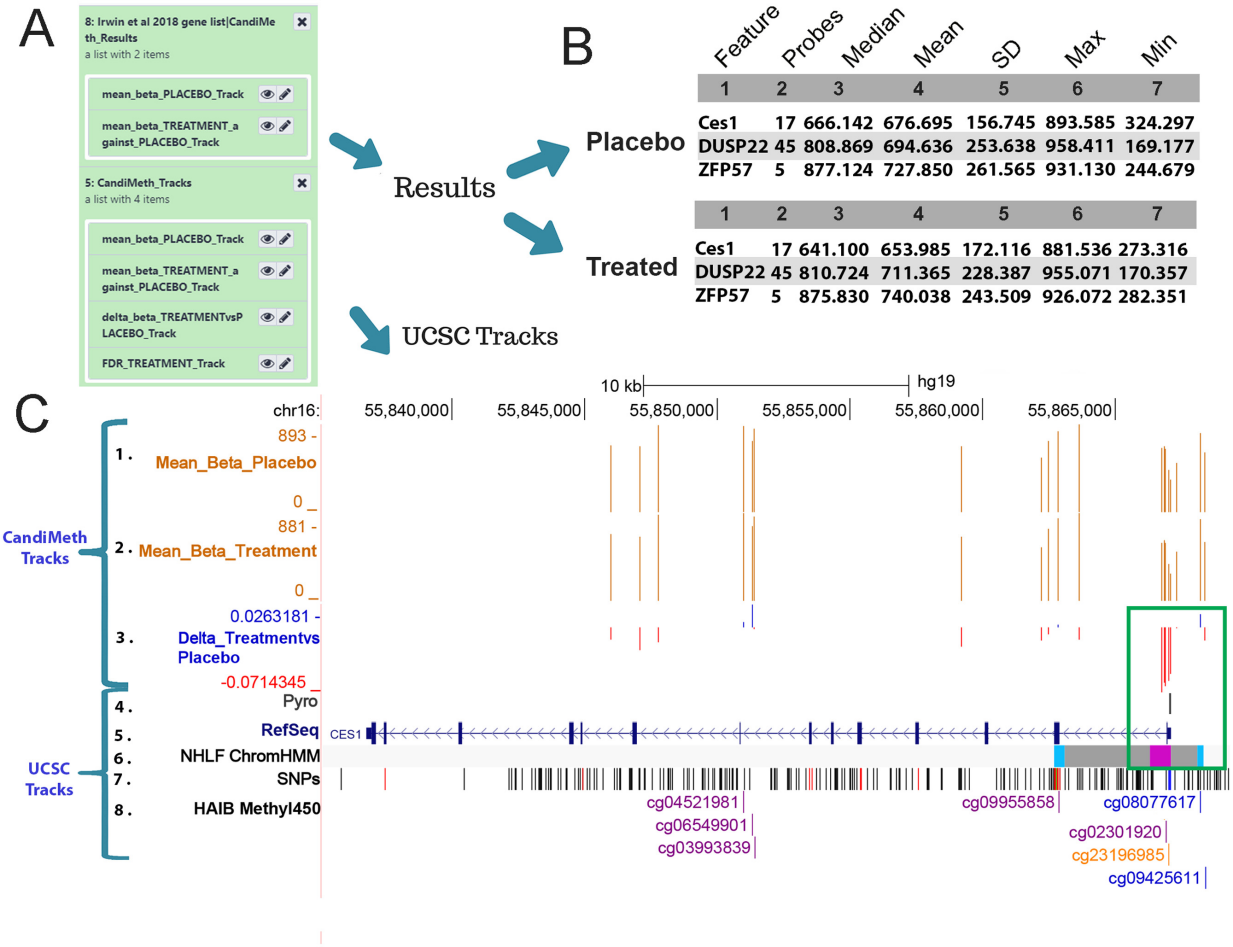
Case Study 2: Using CandiMeth to mine EWAS data. Example data from an EWAS dataset comparing 45 placebo and 41 treated samples from a randomized controlled trial of a folic acid intervention during the second and third trimester of pregnancy. (**A**) Output Results and Tracks from the workflow when the RnBeads differential methylation table and a list of the top-ranked differentially methylated promoters were used as inputs. (**B**) Summary statistics generated by the workflow indicates the number of probes and methylation values (from 1 to 1,000) for the top promoters. (**C**) Tracks view for the CES1 locus showing the absolute levels of methylation (Tracks 1, 2) as well as the most differentially methylated probes (Track 3) located at the promoter (boxed in green). Comparison to ChromHMM data in UCSC (Track 6) shows this to be a poised promoter (pink). Identification of individual CG (numbered in Track 8) facilitated the design of a pyrosequencing assay (Track 4) covering the CG to be validated in the laboratory.

A major application of methylation array technology is in epigenome-wide association studies (EWAS). CandiMeth can provide a very useful tool for quickly examining in detail and quantifying methylation differences around candidate regions identified either by the R-based packages or from the literature. Fig. 5 shows the application of this approach to an EWAS that we have recently published containing data from 86 participants divided into 45 receiving placebo and 41 receiving folic acid supplementation during trimesters 2 and 3 of pregnancy to assess the potential positive effects of prolonging this vitamin supplementation beyond the currently recommended periconception and first trimester periods [26]. Output differential methylation tables from RnBeads were used as input for CandiMeth, together with the names of the top candidate promoters reported earlier. This produced a collection of outputs (Fig. 5A) including a set of tabular Results for the 2 groups Placebo and Treatment, as well as a set of Tracks. The latter included absolute mean β, Δβ, and an FDR track, although the latter returned the message “#No FDR significant sites” (not shown), often the case for EWAS if the sample set was small or the perturbation mild. Clicking through to the tabular results (Fig. 5B) showed tables indicating the number of probes present at each promoter and mean methylation, revealing, e.g., that median methylation at the *CES1* promoter is 2.5% lower in folic acid–treated participants than placebo (666.142 – 641.100 = 25.042/1,000 = 0.025, or 2.5%).

Examination of the CandiMeth Tracks (Fig. 5C) was however also informative here. This BedGraph track type is set by default to scale to the maximum loss and gain on visualization, so that when the UCSC browser is opened on a genomic region of interest, not only are the maximum loss and gain shown, but the graph is scaled to these, meaning that even when small differences in methylation occur, as typically seen in epidemiological studies, the areas of the genome with the greatest changes can be easily identified at a glance. In-house testing has found Δβ tracks to be particularly useful because it can easily be seen whether a feature contains any probes with methylation differences between samples big enough to assess by other means—e.g., pyrosequencing can accurately assess differences in methylation >5%. It can be easily seen from the Δβ (Track 3) that the biggest loss of methylation was 7% (–0.071). The clustering of sites losing methylation at the promoter is also striking (boxed in green) compared to the rest of the gene, suggestive of a step-change in methylation at this important regulatory element rather than a point source. The seamless integration of BLAT [43] meant that designing primers to verify methylation changes could be done very intuitively and the area covered by the assay mapped against the methylation data to confirm that the assay could confirm methylation levels at the exact same location (Fig. 5C Track 4 “Pyro”).

It was also seen from the absolute methylation levels in the samples (Tracks 1, 2, values for promoters given in Fig. 5B) that loss of methylation at the CES1 promoter occurred against a background of high methylation at this region, which suggested that this control element is normally methylated and silenced, a type that often responds to even small losses of methylation. Additional data to corroborate this could be obtained by examining chromatin state data available through the ChomHMM track in UCSC (Fig. 5C, Track 6), which showed that the promoter falls into the “poised promoter” category (colour-coded pink) and is regulated in part by polycomb-group proteins (grey shading). A low likelihood of SNPs at the pyroassay region could be confirmed by examination of the Common SNPs dataset (Fig. 5C, Track 7) and individual CpGs labelled by searching using the UCSC query window, and their status in other public datasets highlighted if desired (Fig. 5C, Track 8). Thus CandiMeth allowed quick examination of candidate regions, quantification of differences specifically at these, the assessment of sites that could be verified in the laboratory, exclusion of confounding SNPs, and eased assay design and gave additional valuable insights through mining of UCSC datasets using only a few simple inputs and no coding.

### Case Study 3: Analysis of methylation at genomic repeats such as LINE1

**Figure 6:**
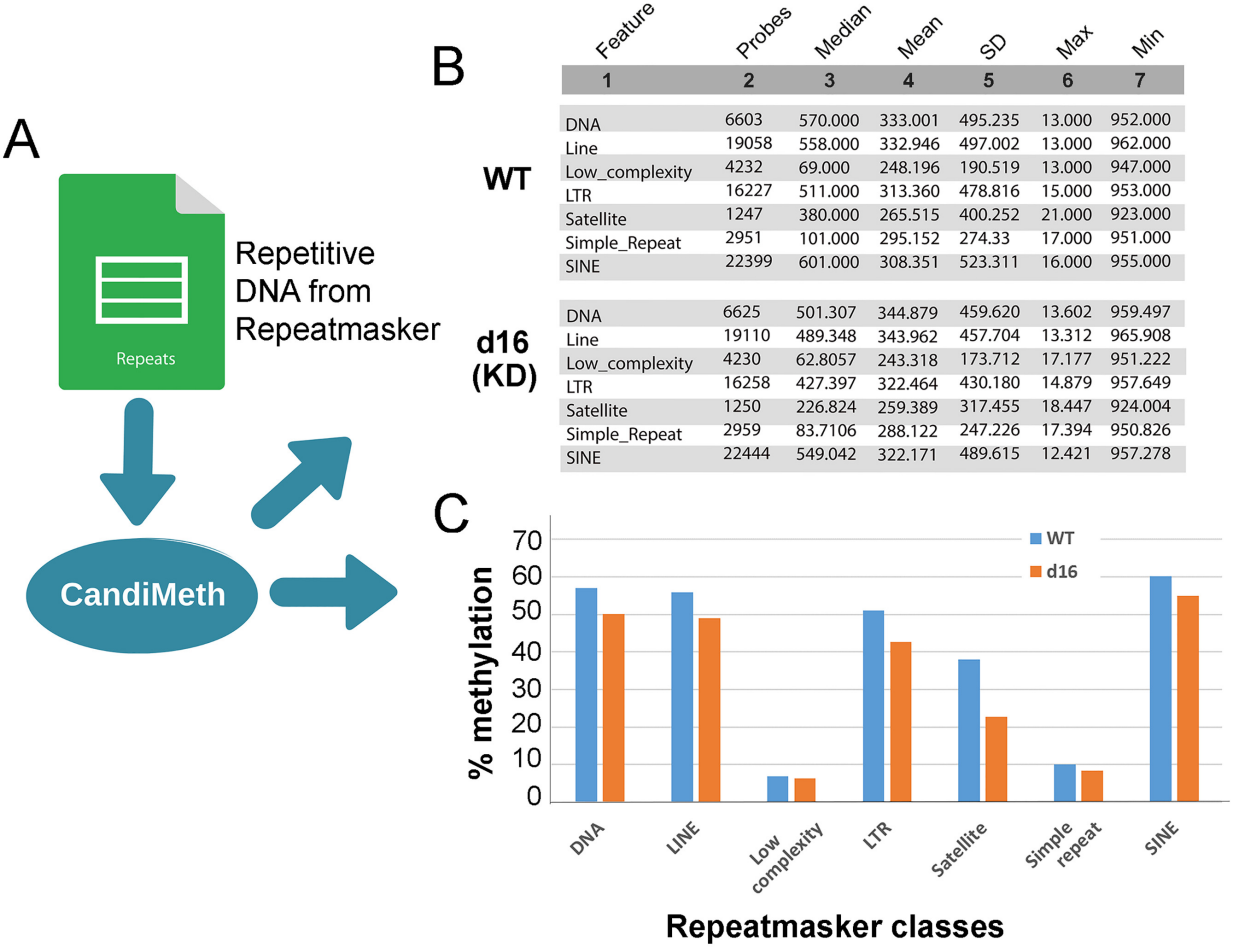
Case Study 3: Analysis of LINEs and SINEs. Use of CandiMeth to give an overview of methylation at repetitive elements. (**A**) Data on repeat location and type from the RepeatMasker track on UCSC has been parsed and made available through the workflow: users can therefore simply type in the name(s) of a class of repeats as a query. (**B**) Example outputs showing probe coverage of repeats on the EPIC array and methylation statistics for each repeat class in a DNMT1 knockdown cell line (d16 KD) versus WT. (**C**) Tables from B were exported, median methylation levels converted to percent, and then graphed to highlight differences between the 2 cell lines: decreases in methylation are seen at some (LINE, SINE, satellite) but not other repeats (low complexity, simple).

Many studies looking for epigenetic changes also try to assess DNA methylation outside of the coding regions. One common approach is to assess methylation at a highly repetitive interspersed repeat such as LINE1, which is found scattered throughout the genome at ∼500,000 copies, so in theory sampling methylation across many locations. This normally has to be done using a separate wet-lab assay such as pyrosequencing because the 450 K and EPIC arrays are designed to cover genes and their associated control elements, not repetitive DNA. However, as has been noted elsewhere [29, 42], a substantial number of probes on the arrays, particularly the EPIC, nevertheless fall within repeats such as LINEs and SINEs. Taking advantage of this, we parsed data from the RepeatMasker track on UCSC to allow mapping and quantification of methylation at the major repeat classes using array data (Fig. 6A). By simply listing the categories of repeat given by RepeatMasker (as in Suppl. Table 4), it is possible to obtain summary statistics indicating the numbers of probes overlapping the respective elements, together with median methylation, and so forth, from any differential methylation table, in this case from our experiment comparing WT and DNMT1-deficient cell lines (Fig. 6B). It can be seen from the tables that very substantial numbers of probes on the EPIC map to the various repeat classes, with ∼20,000 probes in LINE elements spread across the genome, and equal numbers in SINE elements, with satellite repeats near centromeres showing the lowest coverage, at ∼1,000. The summary data were exported to Excel and graphed to highlight where the greatest differences lay (Fig. 6C), which showed that satellite sequences appear to be most demethylated on average, with notable decreases at LINE and long terminal repeat (LTR)-containing elements too, which would include endogenous retroviruses for example, whereas low-complexity and simple repeats show almost no changes, despite good probe coverage (Fig. 6B). Thus CandiMeth allowed straightforward assessment of repeat methylation across the genome without the need for wet-lab analysis and gave novel insights into the differential effects of DNMT1 loss on individual repetitive DNA classes.

### Case Study 4: Analysis of methylation changes seen at a large complex gene locus in multiple samples using parallel processing in CandiMeth

A powerful feature of CandiMeth is the ability to process data from multiple differential methylation analyses at once. To illustrate this, we took 3 sets of comparisons between the independently derived DNMT1 KD cell lines described earlier (d8, d10, and d16), each of which had been compared to the parental WT cell line, and processed them simultaneously. In our earlier publication [25] we had found differences between the variable A and B classes and the variable C class of exons at the important neurodevelopmental gene cluster Protocadherin β (*PCDHB*), with the A and B classes showing severe loss of methylation but no change at the C class. This highlighted differences between these classes, which indicate (i) a hyper-dependence on DNMT1 for maintenance of methylation levels and (ii) a potential difference in methylation dependence that may track with allele usage because the A and B classes show monoallelic expression but not the C class. Here, we wished to examine the neighbouring *PCDHG* locus, which has a similar structure, and see whether the same effect could be seen there.

**Figure 7:**
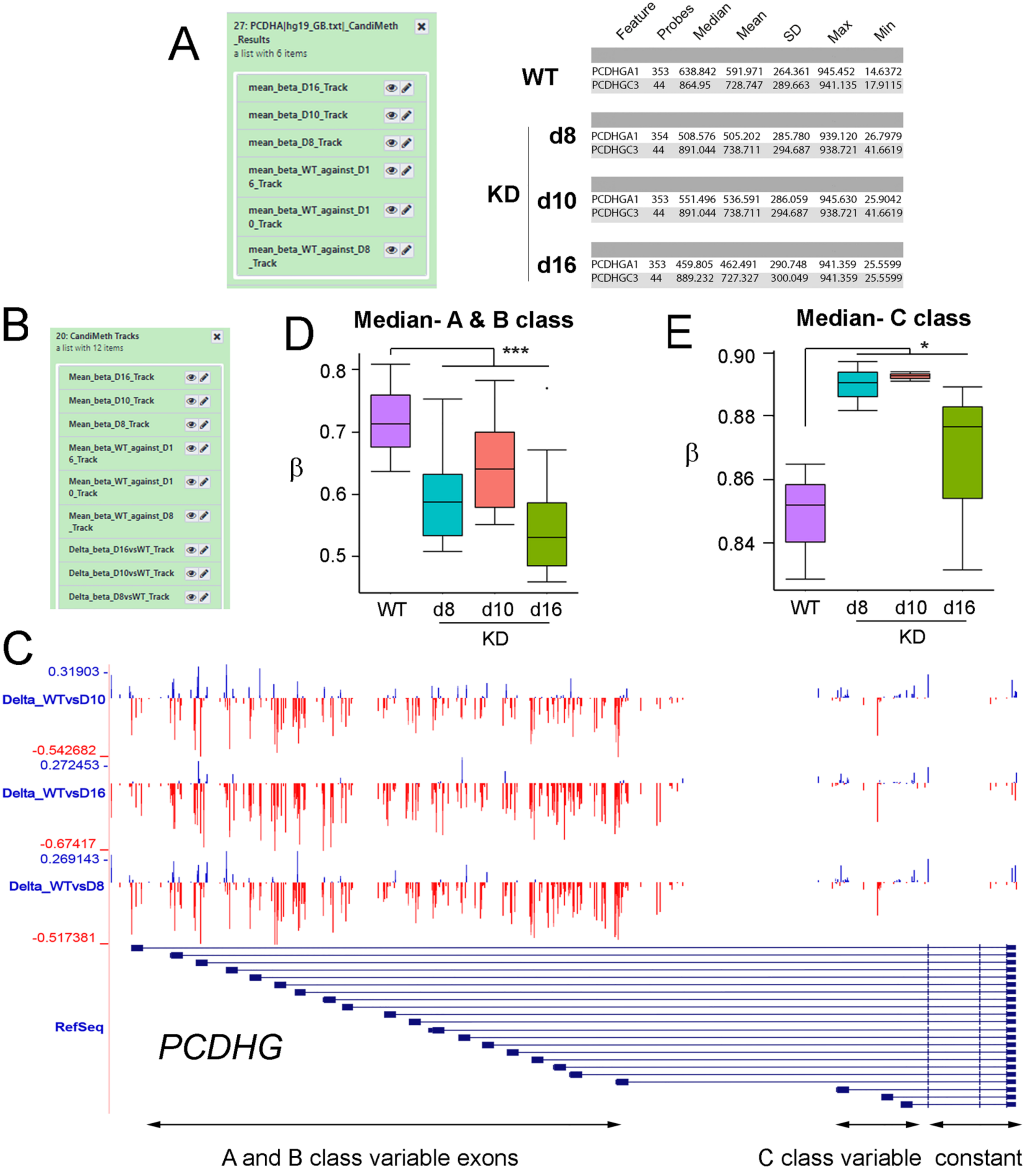
Case Study 4: Parsing data from a complex gene locus using parallel processing. Analysis of methylation at variable exons in the large (∼200 kb) clustered *Protocadherin γ* (*PCDHG*) locus on human chromosome 5. (**A**) Results: changes at the variable exons across 3 independent cell lines deficient in DNMT1 (d8, d10, d16) were scored using the RnBeads tables comparing each to WT as input, together with a list of variable exon names. Example quantitative outputs are shown at right for WT and knockdown (KD) cells. (**B**) Tracks: part of the output set of tracks is shown, which included mean β, differential methylation, and FDR significant sites (not shown) for all cell lines, generated simultaneously in 1 run. (**C**) The UCSC browser view available by following the links in (B). The region covering the A and B class variable exons appears to show more loss of methylation while the C class appear to show predominantly gains; however, this is not exclusive and many probes lie between exons. (**D, E**) Data on probes that lie solely in exons and not introns, obtained through Results (B), were exported and grouped as indicated. The numbers were then converted back into β-values and graphed. This confirmed that methylation was lost on average at the A and B class exons, while the C class predominantly gained methylation. ***P < 0.001, *P < 0.05 by Kruskal-Wallis test.

We therefore generated a candidate region list containing the names of the γ-cluster genes and input this as our candidate feature list input to CandiMeth, together with the 3 differential methylation tables from RnBeads (d8 vs WT, d10 vs WT, d16 vs WT). All 3 sets are processed at once (Fig. 7A, left) and give as outputs data on absolute methylation levels in each KD line as well as the WT parental line (which will not vary), from which summary tables were derived specific to the *PCDHG* exons; example data for 1 A and 1 C exon in each cell line only are shown (Fig. 7A, right). Interestingly, the summary statistics indicated that, while levels of methylation appeared to be decreased across A and B class variable exons at this locus too (e.g., *PCDHGA1* 63.8% median methylation in WT vs 50.9%, 55.1%, and 46% in d8, d10, and d16, respectively), median methylation at C class variable exons appeared to be increasing rather than remaining constant (e.g., *PCDHGC3* 86.5% in WT vs 89.1%, 89.1%, and 88.9% in d8, d10, and d16).

CandiMeth additionally generated Tracks outputs including the full range of tracks for each input table (absolute methylation in WT and each KD, Δβ and FDR for each vs WT). In Fig. 7C we show the differential methylation (Δβ) tracks, from which it appeared that methylation was largely lost across the region of the gene containing the A and B class variable exons (Fig. 7C, region boxed in red), although some gains (blue peaks) could be seen particularly in the d10 track. Additionally, given the size of the region (∼200 kb) it cannot be assessed whether many of the probes lie in the introns rather than the exons themselves. For the C class exons (Fig. 7C, blue box at right) most changes appeared to be gains (blue) although peak sizes were smaller and interspersed with some individual large losses in red. To resolve the exact nature of the changes seen, the tabular data (Fig. 7A) were exported and median values across all A and B exons vs WT generated, converted back to β-value to allow direct comparison to previous results [26], and plotted (Fig. 7D). This clearly showed a general loss of methylation at A and B class exons in all 3 cell lines (*P* < 0.001 vs WT by Kruskal-Wallis test), although the effect was least marked in the d10 cell line. When values were averaged in a similar fashion across the C class variable exons, however (Fig. 7E), we saw a clear gain of methylation in all 3 cell lines (*P* < 0.05, Kruskal-Wallis test). The reason this effect was not noted before is likely to be because our previous examination of the C class exons at PCDHG used the FDR-significant probes only, and as can be seen the magnitude of the gains at the C class exons is much smaller than the losses at the A and B classes (compare scales in Fig. 7D and E).

The analysis thus confirmed and extended observations from our previous study that the A and B class variable exons at the clustered protocadherin loci are hypersensitive to loss of DNMT1 across multiple independently derived cell lines, suggesting a strong dependence on this enzyme for maintenance of epigenetic state at this important neurodevelopmental locus. Furthermore, we have uncovered new evidence for differences between the A and B exons and the C exons, which may reflect divergent transcriptional control, or an increased transcription across the C class exons in response to loss of DNMT1, in line with observations that intragenic DNA methylation is associated with transcription at active loci [9, 43]. In terms of CandiMeth functionality, the study highlights the ability of the workflow to process multiple comparisons in parallel and the value of being able to directly compare the visual outputs and the quantitative data where complex genetic loci are being examined, giving insights into the underlying biology.

## Conclusions and Future Directions

CandiMeth provides a user-friendly non–computationally intensive method of candidate feature investigation. With a minimum of training and no coding, users of CandiMeth can set up and run quite advanced exploratory and confirmatory analyses and use the rich set of existing data in UCSC to formulate and test hypotheses regarding the methylation changes ntat they are seeing.

In future versions, we hope to add support for further methylation processing pipelines and continue to grow the CandiMeth history with additional genomic data such as DNA hypersensitivity sites. In addition to the current pipeline, we also wish to make CandiMeth more intuitive via the creation of a Galaxy tool that would allow the pipeline to be extended to whole-genome bisulphite sequencing or RNA-sequencing data and would also allow further analysis options for those with a private instance of Galaxy.

## Availability of Supporting Source Code and Requirements

Project Name: CandiMeth

Project home page: https://github.com/sjthursby/CandiMeth

Operating system: www.usegalaxy.org

License: GNU GPL

## Availability of Supporting Data and Materials

All supporting data and materials are available in the *GigaScience* GigaDB database [30].

## Supplementary Materials

**Supplementary File 1:** CandiMeth User Guide. A complete Guide to setting up and using CandiMeth, including some background on Galaxy and UCSC browser, how to import the workflow and example files, tutorials on the use of the example data, and further guidance and instruction.

**Supplementary Table 1:** Example Differential Methylation Table generated by RnBeads from GSE90012 for input to CandiMeth. Table comparing wild-type hTERT1604 human fibroblasts (WT) and a clonally derived daughter cell line with depleted levels of DNA methyltransferase 1 (d8) from GEO database entry GSE90012, used as Input 2 to CandiMeth in Case Study 1 (Fig. 2).

**Supplementary Table 2:** MIR gene list used to query data from GSE90012. List of human microRNA genes (*MIR*) used as Input 3 to CandiMeth in Case Study 1.

**Supplementary Table 3:** Methylation summary for MIR genes derived by CandiMeth. Full table of Results for *MIR* methylation in GSE90012 WT vs DNMT1-depleted (d8) cells given as output from CandiMeth (Fig. 2B).

**Supplementary Table 4:** Classes of repetitive DNA sequence that can be analysed. List of repetitive DNA classes as given by RepeatMasker and that can be used as Input 3 by CandiMeth to query datasets, as in Case Study 3 (Fig. 6).

**Supplementary Table 5:** Example Differential Methylation Table from ChAMP. Example data in ChAMP format for use in tutorial as Input 2 in CandiMeth.

## Abbreviations

BLAT: BLAST-Like Alignment Tool; bp: base pairs; CGI: CpG island; ChIP: chromatin immunoprecipitation; CSV: comma-separated values; DMR: differentially methylated region; EWAS: epigenome-wide association studies; FDR: false discovery rate; GEO: Gene Expression Omnibus; GSEA: Gene Set Enrichment Analysis; HGNC: Human Genome Nomenclature Committee; kb: kilobase pairs; KD: knock-down; LINE: long interspersed nuclear element; LTR: long terminal repeat; MIR: microRNA; NCBI: National Center for Biotechnology Information; RefSeq: Reference Sequence; SINE: short interspersed nuclear element; SNPs: single-nucleotide polymorphisms; UCSC: University of California Santa Cruz; WT: wild type.

## Competing Interests

The authors declare that they have no competing interests.

## Funding

Work was funded in part by grants from the Medical Research Council (MR/J007773/1), the EpiFASSTT grant from the ESRC/BBSRC(ES/N000323/1), and the HDHL EpiBrain award from the BBSRC (BB/S020330/1).

## Authors’ Contributions

S.J.T. generated and tested the workflow and accompanying datasets and drafted the manuscript; D.K.L. helped with SED and workflow debugging; R.E.I. provided supervision and feedback on early versions; K.P. and S.D.Z. provided guidance and comments; C.P.W. designed the study, generated early versions of the workflow, and co-wrote the manuscript; all authors commented on and approved the final version.

## Supplementary Material

giaa066_GIGA-D-19-00417_Original_Submission

giaa066_GIGA-D-19-00417_Revision_1

giaa066_Response_to_Reviewer_Comments_Original_Submission

giaa066_Reviewer_1_Report_Original_Submission Miriam PayÃ¡ Milans -- 12/24/2019 Reviewed

giaa066_Reviewer_1_Report_Revision_1 Miriam PayÃ¡ Milans -- 4/29/2020 Reviewed

giaa066_Reviewer_2_Report_Original_Submission Yuan Tian -- 12/30/2019 Reviewed

giaa066_Supplemental_Files
