# Supplementary material for: CandiMeth: Powerful yet simple visualization and quantification of DNA methylation at candidate genes
Source: Gigascience. 2020 Jun 22;9(6):giaa066. doi: 10.1093/gigascience/giaa066 (PMC7307318; doi:10.1093/gigascience/giaa066)
Supplement: giaa066_GIGA-D-19-00417_Original_Submission [file giaa066_giga-d-19-00417_original_submission.pdf]

GigaScience

CandiMeth: Powerful yet simple visualization and quantification of DNA methylation at candidate genes

--Manuscript Draft--

**Manuscript Number:**	GIGA-D-19-00417
**Full Title:**	CandiMeth: Powerful yet simple visualization and quantification of DNA methylation at candidate genes
**Article Type:**	Technical Note
**Funding Information:**	Economic and Social Research Council (ES/N000323/1) — Professor Colum P Walsh
**Abstract:**	Background<br><br>DNA methylation microarrays are widely used in clinical epigenetics and are often processed using R packages like ChAMP or RnBeads by trained bioinfomaticians. However, looking at specific genes requires bespoke coding which wet-lab biologists or clinicians are not trained for. This leads to high demands on bioinfomaticians, who in turn may lack insight into the specific biological problem. We therefore wished to develop a tool for mapping and quantification of methylation differences at candidate genomic features of interest, without using coding, to bridge this gap.Findings<br><br>We generated the workflow CandiMeth (CANDIdate METHylation) in the web-based environment Galaxy. CandiMeth takes as input any table listing differences in methylation generated by either of the popular R -based packages above and maps these to the human genome. A simple interface then allows the user to query the data using lists of gene names. CandiMeth generates 1)Tracks in the popular UCSC genome browser with an intuitive visual indicator of where differences in methylation occur between samples, or groups of samples 2) Tables containing quantitative data on the candidate regions, allowing interpretation of significance. In addition to genes and promoters, CandiMeth can analyse methylation differences at LINEs and SINEs. Cross-comparison to other open-resource genomic data at UCSC facilitates interpretation of the biological significance of the data and the design of wet lab assays to further explore methylation changes and their consequences for the candidate genes.Conclusions<br><br>CandiMeth allows rapid, quantitative analysis of methylation at user-specified features without the need for coding and is freely available here (http://bit.do/canidmeth-github).
**Corresponding Author:**	Colum P Walsh, Ph.D.<br>University of Ulster<br>Coleraine, Co. Londonderry UNITED KINGDOM
**Corresponding Author Secondary Information:**	
**Corresponding Author's Institution:**	University of Ulster
**Corresponding Author's Secondary Institution:**	
**First Author:**	Sara-Jayne Thursby
**First Author Secondary Information:**	
**Order of Authors:**	Sara-Jayne Thursby<br>Darin K Lobo<br>Kristina Pentieva<br>Shu-Dong Zhang, Ph.D.<br>Rachelle E Irwin

Powered by Editorial Manager® and ProduXion Manager® from Aries Systems Corporation

	Colum P Walsh, Ph.D.
**Order of Authors Secondary Information:**	
**Additional Information:**	
**Question**	**Response**
Are you submitting this manuscript to a special series or article collection?	No
**Experimental design and statistics**<br><br>Full details of the experimental design and statistical methods used should be given in the Methods section, as detailed in our Minimum Standards Reporting Checklist. Information essential to interpreting the data presented should be made available in the figure legends.<br><br>Have you included all the information requested in your manuscript?	Yes
**Resources**<br><br>A description of all resources used, including antibodies, cell lines, animals and software tools, with enough information to allow them to be uniquely identified, should be included in the Methods section. Authors are strongly encouraged to cite Research Resource Identifiers (RRIDs) for antibodies, model organisms and tools, where possible.<br><br>Have you included the information requested as detailed in our Minimum Standards Reporting Checklist?	Yes
**Availability of data and materials**<br><br>All datasets and code on which the conclusions of the paper rely must be either included in your submission or deposited in publicly available repositories (where available and ethically appropriate), referencing such data using a unique identifier in the references and in	Yes

Powered by Editorial Manager® and ProduXion Manager® from Aries Systems Corporation

the “Availability of Data and Materials” section of your manuscript.<br><br>Have you have met the above requirement as detailed in our Minimum Standards Reporting Checklist?	

Powered by Editorial Manager® and ProduXion Manager® from Aries Systems Corporation

Manuscript Click here to download Manuscript CandiMeth MS GigaSciencev1.pdf

Editorial Team
GigaScience

Coleraine, 4th Oct 2019

Dear Editors

We wish to submit the attached MS *"CandiMeth: Powerful yet simple visualization and quantification of DNA methylation at candidate genes"* for consideration at *GigaScience*. In it, we report on the development of an open-source tool (Galaxy workflow) for handling large-scale data. The journal seems very appropriate for the work because of the strong bioinformatics papers published there and it's special interest in Galaxy work.

Furthermore, we were recommended to submit to you by **two members of the Editorial Board**, Dr Titus Brown at UC Davis and Prof. Stephan Beck at UCL in London. The first author met Dr. Brown at a Galaxy workshop in Portland, having been awarded a bursary to attend. She presented the work there and it was well-received, after which she spoke with Dr. Brown.

Prof. Beck's lab has developed *ChAMP,* the most popular end-to-end *R* package for analysing DNA methylation arrays currently available (ranked 183/1823 for Bioconductor packages, hundreds of downloads per month). Our new workflow takes up where *ChAMP* leaves off and allows wet-lab biologists to more easily analyse methylation differences without the need for coding in *R*. It also works with the second most popular *R* package for this, *RnBeads* (ranked 268/1823 in Bioconductor). Furthermore it can be adapted simply by the user to work with any other *R*-based methylation preprocessing package. Prof. Beck has seen the MS and indicated it looked suitable for submission to *GigaScience*. In addition to the MS, we provide a Tutorial and example datasets to analyse, with all materials and workflow available at GitHub and through Galaxy.

The authors declare no competing interests: all authors have seen and approved the MS and the content has not been submitted or published elsewhere.

Sincerely

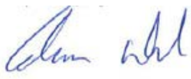

Colum Walsh (Ph.D.),
Professor of Genetics,
Group Leader, Genomic Medicine Research Group,
Biomedical Sciences Research Institute,
Centre for Molecular Biosciences,
University of Ulster,
Coleraine BT52 1SA
UK
Email: cp.walsh@ulster.ac.uk
Ph.+44 28 7012 4484

CandiMeth: Powerful yet simple visualization and quantification of DNA methylation at candidate genes

Sara-Jayne Thursby[1], Darin K. Lobo[1,4], Kristina Pentieva[2], Shu-Dong Zhang[3], Rachelle E. Irwin[1], Colum P. Walsh[1,*]

[1]Genomic Medicine, [2]Nutrition Innovation Centre for Food & Health (NICHE) and [3]Stratified Medicine Research Groups, School of Biomedical Sciences, Ulster University,

1 Cromore Road, Coleraine, BT52 1SA UK

[4]Current Address: Republic Polytechnic, 9 Woodlands Ave 9, Singapore 738964

Sara-Jayne Thursby, Thursby-SJ@ulster.ac.uk

Darin Kris Lobo, darin.lobo@gmail.com

Kristina Pentieva, k.pentieva@ulster.ac.uk

Shu-Dong Zhang, sd.zhang@ulster.ac.uk

Rachelle Irwin, r.irwin@ulster.ac.uk

Colum Patrick Walsh, cp.walsh@ulster.ac.uk

*corresponding author: +44 28 7012 4484

Abstract

Background: DNA methylation microarrays are widely used in clinical epigenetics and are often processed using *R* packages like *ChAMP* or *RnBeads* by trained bioinfomaticians. However, looking at specific genes requires bespoke coding which wet-lab biologists or clinicians are not trained for. This leads to high demands on bioinfomaticians, who in turn may lack insight into the specific biological problem. We therefore wished to develop a tool for mapping and quantification of methylation differences at candidate genomic features of interest, without using coding, to bridge this gap.

Findings: We generated the workflow *CandiMeth* (CANDIdate METHylation) in the web-based environment Galaxy. CandiMeth takes as input any table listing differences in methylation generated by either of the popular *R*-based packages above and maps these to the human genome. A simple interface then allows the user to query the data using lists of gene names. CandiMeth generates 1)Tracks in the popular UCSC genome browser with an intuitive visual indicator of where differences in methylation occur between samples, or groups of samples 2) Tables containing quantitative data on the candidate regions, allowing interpretation of significance. In addition to genes and promoters, CandiMeth can analyse methylation differences at LINEs and SINEs. Cross-comparison to other open-resource genomic data at UCSC facilitates interpretation of the biological significance of the data and the design of wet lab assays to further explore methylation changes and their consequences for the candidate genes.

Conclusions: *CandiMeth* allows rapid, quantitative analysis of methylation at user-specified features without the need for coding and is freely available here (http://bit.do/canidmeth-github).

Keywords: Galaxy, Methylation, Workflow, DNA methylation, arrays, epigenetics, EWAS

Introduction:

Epigenetics can be defined as stable, and most often heritable, changes to the chromatin which do not alter the DNA sequence itself but still impact gene expression and/or are required to maintain genomic stability [1]. These modifications consist of reversible marks such as cytosine DNA methylation or histone modifications, each critical to gene expression regulation, imprinting, X-inactivation and many other processes from mammalian gestation to later life [1].

Cytosine DNA methylation is the most common and thoroughly investigated of these epigenetic alterations. It is characterised by the addition of a methyl group to a cytosine residue, many of which are located within so-called CpG islands (CGI) close to gene promoters [3]. High levels of DNA methylation at promoters aids in the stable long-term repression of the cognisant genes, such as can be seen on the inactive X chromosome in mammals [4]. Methylation at control elements such as insulators or enhancers can also help regulate regional gene expression, with multiple examples being seen among imprinted genes [5] or gene clusters such as the protocadherins [6]. High levels of methylation are seen on selfish DNA elements such as endogenous retroviruses, where it plays an important role in their suppression [7] as well as at inert regions of the genome like pericentromeric repeats [8]. More recently, methylation through the body of the gene has been recognised as contributing to maintaining gene transcription levels at highly-expressed genes [9,10]. As well as showing such developmental programming, DNA methylation is susceptible to environmental influence, with inputs such as diet [11, 12] and exposure to pollutants like cigarette smoke [13] having clear and reproducible effects on methylation levels, sparking great interest in analysis at a population level, particularly in humans [14].

Advances in sequencing technology have allowed us to quantify and analyse methylation via whole genome bisulphite sequencing at approximately a 28 million CpG resolution [15].

While this technique remains the gold standard for whole genome methylation assessment, it can be very expensive, and when there are hundreds of samples to be tested and analysed prohibitively so, and quantifying small differences reproducibly between multiple samples is also challenging. An alternative technology known as a microarray, which predates the era of whole genome bisulphite sequencing, is often a popular solution for such cases, where a lower CpG resolution is satisfactory, but where there is greater inter-sample reproducibility. A popular choice here is the Illumina Infinium Methylation BeadChip array [16], which currently covers 850,000 CpG sites across the human genome, including 99% of *RefSeq* genes and large numbers of enhancers and other features. This can help elucidate the effects of an intervention across hundreds of samples in a cost effective manner. There are many packages across multiple computational languages to analyse the outputs from these arrays such as *RnBeads* [17] or *ChAMP* [18] but these pipelines operate in the statistical programming environment *R*, and require some coding. Additionally, the output file formats can be overwhelming and difficult to investigate further without experience in data analytics and bioinformatics. This situation is exasperated by the typically higher number of samples in epidemiological or intervention studies where such arrays are commonly used.

To help solve this predicament, we developed a *Galaxy* workflow known as *CandiMeth*, which takes the main output from such methylation analysis pipelines and pairs this with a list of features that the user may wish to investigate. The workflow first generates tracks showing both absolute methylation levels in samples and differences in methylation between samples. These can be viewed via the UCSC genome browser and overlaid with other available tracks such as CpG island, enhancers, ChIP data etc. to allow data exploration and more intuitive analysis. The workflow can then help confirm any patterns observed by quantifying data across the identified regions or features e.g. methylation differences at specific sets of genes between cases and controls, or examining whether methylation

differences go hand-in-hand with a particular histone modification. The workflow removes the need for further analysis in *R* and increases reproducibility by using an automated process, but in a more user-friendly manner.

Methods

CandiMeth is designed to work downstream of DNA methylation analysis pipelines in *R*. It was developed initially using RnBeads as reference, but has been subsequently successfully run with *ChAMP* and other packages (see below). *ChAMP* (RRID: SCR_012891) [19] and *RnBeads* (RRID: SCR_010958) [17,20] are end-to-end pipelines in *R* which can take raw data files such as *IDAT*s and *bam* files from microarray readers or sequencers and process these to allow data exploration, visualisation and comparison. For array data, which is the main area where *CandiMeth* addresses an unmet need, *IDAT* files containing raw values for the red and green channels for each of ~850K probes are exported from the microarray reader. *RnBeads/ChAMP* can perform quality control, remove probes with low signal or overlapping with single nucleotide polymorphisms (SNPs) and provide a cleaned dataset giving absolute levels of methylation as beta (β) or *M* values. The packages can also facilitate exploratory visualisation through principle components analysis or similar and allow grouping of data prior to looking for differential methylation. Probes showing substantial differences in methylation (delta beta: Δβ) can be identified and then ranked based on a variety of parameters, including probability of occurrence (*p* value), delta beta, false discovery rate (FDR) or a combination of several of these. The packages can look for enriched gene sets using gene ontologies/GSEA [21] and visualise differences for annotated categories of array probe such as promoter and gene body.

While packages for array analysis provide genome-level data such as whether promoters in general are losing or gaining methylation, querying specific gene sets which might give more biological insight cannot be easily done in this or other *R* packages with similar functionality

without extracting the processed dataset and writing bespoke code. Visualization of the data against the genome map is also of great attraction for the wet-lab biologist, but is also not easily done within these packages. While *RnBeads* can map methylation values to the genome as customised tracks, this can only be carried out if a local instance is installed on the user's server, which requires substantial investment for set-up and maintenance. ChAMP does not currently provide tracks at all, to our knowledge. Typically, many biologists have specific genes which are of interest to them, or they may want to examine the area in which top sites are located and determine if adjacent probes are also losing or gaining methylation. A ready way of assessing the degree to which methylation is changing across a particular region and the exact location of the probes also greatly facilitates the design of gene-specific assays such as primer sets for pyrosequencing or clonal analysis. It is also generally of interest to try and leverage the enormous pool of publicly-available data accessible through UCSC genome browser tracks to explore possible novel correlations between methylation changes in your dataset and other genome characteristics such as replication timing, histone modifications, or similar.

We therefore wished to develop a user-friendly non-computationally intensive method of candidate feature investigation which avoided command-line but was more powerful than browser-only interfaces. To this end we chose the *Galaxy* platform (*Galaxy*, RRID:SCR_006281) which is a free open-source environment for user-friendly and reproducible bioinformatics [22]. It provides a variety of data manipulation and analysis tools via a web interface with no prior installation or dependency packages required, with results stored within the *Galaxy* infrastructure and every action producing a new history entry so the original data is never compromised via destructive edits. *Galaxy* also allows users to aggregate analysis steps into repeatable pipelines called workflows which can be easily shared, along with the histories, via *URL* or username. These can allow biologists with little

bioinformatics experience to conduct complex analyses on their own data within a system which has a low maintenance requirement and with little worry over data storage or data corruption. Moreover, workflows can be published to a repository such as *GitHub* (RRID:SCR_002630) or *MyExperiment* (RRID: SCR_001795) [23] or within a scientific journal – further encouraging open data science and reproducibility. *Galaxy* also provides many plugins such as interactive visualisation software to view results, the option to export results to genome browsers and the option to configure tools, or indeed an entire *Galaxy* instance, to the desired end-user needs.

Overview of workflow

The main process undertaken by *CandiMeth* is to take as input the methylation data from an *R* pipeline such as *RnBeads* or *ChAMP* and 1) Visualise the data as tracks in the UCSC genome browser and 2) Analyse the methylation differences relative to genomic features specified by the user. The workflow is comprised of 3 main steps; Inputs, Feature Mapping and Analysis (Fig.1). There are also 4 items required at input stage: the user must a) indicate the R package used with the keywords ‘Rnbeads’, ‘Champ’ or ‘Custom’, then supply b) the methylation data, c) a list of the genes of interest, and d) specify the human genome build to be used e.g. *hg19*. The basic workflow for *CandiMeth* is that the genes of interest are mapped to the reference genome and then cross referenced with the input methylation data to get feature- specific statistics. The workflow can currently look at either the promoters (-500 to +1 bp relative to transcription start site; suffix “_P” on results) or gene bodies (the transcription unit; “_GB”), or both parts of the gene together (“_all”). We have found this to be a particularly useful split, since the current consensus is that promoters and gene bodies can show opposite methylation patterns, with methylation at the promoter is largely associated with repression, whereas gene body methylation instead is a feature of transcribed genes. Outputs are then grouped in the history into two types, Results or Tracks (Fig.1). The

methylation data from the R packages is a standard differential methylation table as routinely generated, and either a single table comparing two groups, or several tables can be processed at once as inputs, e.g. comparing different experimental conditions with the control. Each comparison will result in a separate table and tracks, grouped together and given a condition-specific identifier to avoid confusion. The *CandiMeth* workflow, together with the example datasets used and a step-by-step tutorial, are available here (http://bit.do/candimeth-github).

Example outputs

To illustrate the type of analysis that can be done, Figure 2 shows outputs from one of the example dataset runs. Here we used as input one of our previously-published differential methylation tables generated by *RnBeads* (NCBI Gene Expression Omnibus (GEO) identifier GSE90012, the table is also given as Suppl. Table 1) [24]. The experiment compared wild type hTERT1604 human fibroblast cells (WT) and a clonal derivative with a stable knockdown (KD) of the maintenance DNA methyltransferase DNMT1 (d8 KD), which gave large alterations in DNA methylation levels, very suitable for the purpose of illustration here. The second item needed for *CandiMeth*, namely features of interest, was in this case a set of microRNA (*MIR*) genes not analysed in the original paper, which was input here simply as a list of names (given in Supp. File 2). *CandiMeth* first mapped the miR locations to the human genome (in this case hg19), then analysed the co-occurrence of probes at these locations. The results appeared in Galaxy as two grouped sets of datasets (Fig.2A): 'Mir Cluster | hg19 all | CandiMeth Results' and 'CandiMeth Tracks'.

The Results set contained an output table for each condition, namely KD (d8) and WT cells (Fig.2B, first 5 rows of each shown). Each table consisted of seven numbered columns. It should be noted here that methylation values from the array are expressed as a number from 1 (no methylation) to 1000 (fully or 100% methylated) to facilitate visualisation. The numbered columns correspond to 1) Feature, the candidate region of interest, in this case each of the

MIR in the initial list; 2) Probes, the number of array probes which are found in the specified feature; 3) Median, the methylation value which is the median of all probes mapping to that feature e.g. 626.208/1000 is the median of all probes at *MIR1185-1*, or 62.6% methylated; 4) Mean, the mean methylation value across all probes; 5) SD, the standard deviation; 6)Max., the maximum probe value seen in the feature and 7) Min, the minimum probe value (Fig.2B). It can be seen that methylation values are much lower in the DNA methyltransferase-depleted cells (d8) for each miR compared to the parental or WT cells e.g. *MIR1185-1* 62.6% median methylation in d8 vs 72.2% in KD. It can be seen that, while usually in reasonable agreement, in some cases the median and mean vary substantially, and having data on the numbers of probes can be useful on deciding confidence in the results and on any threshold to be applied.

In the Tracks folder *CandiMeth* also generated four tracks on the UCSC genome browser (Fig.2C, 1-4), which can be visualised by clicking on the eye icon on the Galaxy datasets under *CandiMeth* Tracks in Fig.2A (clicking on each track overlaid it on the previous one to generate the cumulative view shown).

Tracks 1 and 2 are absolute methylation tracks, denoted as ‘Mean_beta’ in CandiMeth outputs. These show the methylation per probe for all probes in the differential methylation table that passed QC and other screening steps in RnBeads, and not just the feature-specific (here *MIR*) probes, as we have found that the genomic methylation context is very valuable to consider when looking at features. In other words, even if Promoters is selected at input, the tracks will show all probes, including those in gene body and other regions. Track 1 is the DNMT1-depleted cell line (‘Mean_beta_d8’) data, and Track 2 is from the WT cells (‘Mean_beta_WT’).

Track 3 is the delta beta track (‘Delta_d8vsWT’) showing the difference between methyltransferase-deficient and WT cells. These are BedGraph files like Tracks 1 and 2, but since methylation can be higher or lower in one sample versus another, the visualisation is

different from the absolute methylation tracks. Instead, gains in methylation in the experimental condition are shown as blue columns above the zero (no change) line, and losses are shown as red columns below the line, with a change of +1 being 100% increase and -1 being -100% i.e. an array probe going from 100% methylated to 0% methylated. The Delta track also allows the user to see how many array probes in a region are showing large differences in methylation and if a Differentially Methylated Region (DMR) identified by RnBeads extends further than originally estimated [25].

Lastly, an FDR track (‘FDR_D8’, Track 4) was also produced: this only showed information for those probes where the R package has assessed the false discovery rate to be below 0.05, as this is a statistical cut-off implemented by many array users. This is an excellent method for visualising only CG which have high-confidence differences in methylation between samples. Here, only a single probe passed the FDR threshold and is shown: the absolute methylation level at the probe is given, as *p* values would not scale correctly.

One of the most powerful features of using this approach is that data can easily and more intuitively be compared to other UCSC tracks (Fig.2C, 5-7). The specific CpG site can be identified in UCSC for example by right-clicking on the column on the track, or by typing the CG identity into the UCSC browser search window, which will then pull out a track with the site highlighted, in this case the ENCODE project’s HAIB Methyl450 (Fig.2C, Track 5). A particularly useful took in this context is UCSC’s BLAT, which can be used to help ensure primers designed to verify methylation differences at specific regions of interest by pyrosequencing or similar do indeed overlap the crucial sites (Fig.2C Track 6, Pyro), in this case the FDR-significant site. Off-the-rack assays for each CG on the EPIC array can also now be purchased commercially. Other UCSC tracks shown in Fig. 2C include the RefSeq track (Track 7), invaluable for identifying well-curated genes rather than predicted or rare products. These tracks were all overlaid on the *CandiMeth* tracks, allowing the user to see if

methylation changes were located in or near any of these features. These are examples only, any track available through UCSC or which can be called through *Galaxy* can potentially be aligned with the *CandiMeth* tracks.

Data Preparation and inputs:

A Step-by-Step Guide document is available here (http://bit.do/candimeth-github) , here we will describe more general features of the workflow. As indicated, CandiMeth runs in the Galaxy environment: users must first create an account and copy the CandiMeth test history and workflows to their account, as explained in the Guide. Once these simple steps have been carried out the first time, they do not need to be repeated. When *CandiMeth* is being run, the initial window will look as shown in Fig.3: the workflow occupies the central window, while the example data and datasets required for the workflow are in the History window at right; the left window Tools will not be used. One initialising, the workflow window will look as shown, with one Yes/No choice and four fields (numbered 1-4) to fill in. We recommend saving the outputs of *CandiMeth* to a new history when initiating the pipeline. This will 1) enable you to continue working on other tasks while *CandiMeth* is running in the background – the workflow can take a while to run depending on server usage and 2) segregate the current job from the reference datasets in the CandiMeth initial history, which avoids cluttering the initial history or causing problems if a particular run fails and generates incompletely processed datasets. The four fields are the 4 forms of inputs required, as indicated in the example above and dealt with below.

Input Type 1: R package used

CandiMeth works downstream of R-based packages which are designed to process epigenome-wide datasets. The two most popular packages (by *Bioconductor* download) *ChAMP* and *RnBeads* both automatically generate tabular data outputs which are suitable as input for *CandiMeth* without further processing, but the tables are in slightly different

formats. Therefore, *CandiMeth* users should select either '*RnBeads*' or '*ChAMP*' when asked what *R* package was used. *CandiMeth* also supports other packages via a 'Custom' keyword.

Input Type 2: Differential Methylation Table

The user needs to identify the location of, or upload directly, a copy of the output table from the *R* package containing the differential methylation data. For *RnBeads* this can be found via the html interface by opening "differential_methylation_html" and choosing the desired comparison table. Once uploaded, the differential methylation table must be converted to a dataset collection through a 1-step operation (see the Step-by-step Guide, available here at http://bit.do/canidmeth-github and [26]), which allows all the data from the table to be processed at once. An example table is available in dataset collection format in the CandiMeth default history: the raw table itself is also available as Supp. Table 1. In addition, an example ChAMP output is also available as Suppl. Table 5 in the CandiMeth History.

If the custom option is chosen at Input 1 above, the user can input a data frame of any origin as long as it follows the default *CandiMeth* format namely: Chromosome; Start; cgid; mean.X; mean.Y; the difference between the two groups; and the FDR corrected p value (were X and Y equal the names of the experimental and control groups respectively). Data frames can also be rearranged in *Galaxy* using the text manipulation tools "cut" and "join" within the *Galaxy* tool panel to produce an acceptable input table. We hope to extend the number of preformatted options beyond *RnBeads* and *ChAMP* to reduce the need for custom inputs in future.

Input Type 3: Gene Features of Interest

Here the user can choose which features they want to investigate. This can be done in a customised fashion, but commonly biologists initially want to see how much methylation is present across well-defined genomic features such as genes. This can easily be done in *CandiMeth* by following the commands >Get Data >upload File >paste/Fetch and then typing

the official gene names, one per line, into the window which opens there (see Step-by-Step Guide). Alternatively, they can be uploaded as a list in a tab-delimited file format at this step. To facilitate initial trials, the miR gene names used above have been preloaded into the default CandiMeth history for use, and are also supplied as Supp. Table 2. The features associated with the gene names are then mapped to the genome using the genomic data discussed next.

Input Type 4: Genome Information

An important part of the CandiMeth workflow is the parsed human genome information used to assign array probes to various genomic features. Example human genome build information used for the mapping part of the CandiMeth pipeline can be found within the CandiMeth history (right hand pane in Fig.3). The data provided here covers two genome assemblies, hg19/hg38 and will aid the mapping of candidate features to promoters, whole gene body region or both (hg19_all option) as defined by RefSeq [27].

Using CandiMeth, users can query RefSeq-defined genes or repeats to obtain the same types of information as can be obtained by analysis in an R package. One advantage here however is that the simultaneous visualisation allows the user to inspect the match between probe location and gene structure for candidate regions of interest: for example, the initial screen may indicate changes in promoter methylation from the manifest-defined promoter, when inspection shows all of the probes lie in the first exon of a single-exon gene, and therefore are in fact gene body, the discrepancy being due to the definition of promoter in the manifest. CandiMeth allows the user to refine or alter the promoter definition to exclude bases downstream of the TSS for example, and re-evaluate. An approximation of promoter areas of these RefSeq genes was generated for the example data analysis and was defined as the region from 500 bp upstream to the first base (-500-> +1bp) and is available in the CandiMeth history [28] mentioned above. Similarly, probes were also parsed into gene body

and repeat categories for CandiMeth to facilitate user analysis of affects over these types of genomic intervals for their candidate genes of interest.

Processing steps

Figure 4 shows a workflow editor view of CandiMeth: different sections have been numbered for ease of reference here.

1.Inputs: Inputs are indicated at left; R package used to generate the table (1.1) differential methylation table (1.2), features of interest (1.3), and parsed genome information specific to that type of interval e.g. promoters (1.4). Once the four input types have been decided- examples of each of these were given above- the workflow proceeds as follows.

2. Generation of a standardised data frame between RnBeads and ChAMP: The R package keyword used (either 'Rnbeads', 'Champ' or 'Custom') is combined with the differential methylation table (2.1) to form an extra column detailing which R package 1.2 came from. A table is then output showing the chr, start, cgid, mean methylation between control and experimental groups, the difference between these experimental groups and FDR corrected p value (2.2). Subsequently, the end co-ordinates for each cg site are calculated and added to this table (2.3), so the data can be configured to run on UCSC genome browser at a later stage in the workflow.

3,4. Track generation and naming: Differential Table inputs from RnBeads (1.2) are converted into a variety of tracks compatible with UCSC Genome Browser. These include two absolute methylation tracks (3.4, 3.5) in this case, one false discovery rate (FDR) track showing only FDR significant sites (3.1) and one delta beta track (3.3), showing the difference in beta value between the two absolute methylation tracks. Track and results names (4.1 – 4.4) are also generated from the differential table inputs: this is an important step as both absolute methylation data for individual samples and a number of types of comparison data must be separated and given logical and intuitive names to allow easy

identification among the multiple output datasets. The workflow utilises a number of pre-existing tools available in Galaxy to carry out these steps (Table 1).

5. Merging of tracks and names: Following track creation (3), the resultant tracks and their names are merged into separate dataset collections (5.1 – 5-4) and then collapsed into singular dataset collections (5.5, 5.6), one for all comparative tracks (5.5), one for all comparative track names and one for all absolute methylation (mean beta) tracks (5.4) with its associated names (5.7). The mean beta tracks will be used for feature investigation later in the workflow. The results here are compilations containing information on methylation at each probe across the genome in each sample, or the differences in methylation at specific probes between pairs of samples.

6. Feature mapping: Features of interest (1.3) input by the user such as a particular set of genes are mapped using the Join command (6.1) to the specified genome release information (1.4). The Join command creates repeated columns that are no longer required and are therefore discarded (6.2) using the Cut tool, and unique records extracted (6.3). The output here is a set of genomic coordinates matching only the specific features of interest e.g. a specific set of genes.

7. Compilation of methylation data for features: The dataset collection containing now correctly named absolute methylation tracks (5.7) is now joined with the mapped features of interest (7). This allows the generation of feature-specific statistics.

8,9 Outputs: Feature-specific statistics such as mean methylation over all probes in each feature, median, maximum etc. (see below) are tabulated and form one major output (8). The comparative tracks (generated in 3) are also given unambiguous final names, collated and output as a dataset collection called ‘CandiMeth Tracks’ (9, with green stars marking final output states).

Output Files

The CandiMeth workflow produced as indicated above under Example output two main types:

Tables

Results tables all follow the same layout; feature name, probe coverage, median methylation, mean methylation, standard deviation, maximum and minimum. A partial example of a Tabular output for the set of miRs used in the example above is shown in Fig.2B (first five lines) and given in full in Suppl. Table 3. Methylation values for the features can then be plotted within Galaxy via their integrated visualization software or the Table can be exported and downloaded then plotted within the user's preferred visualization software such as Prism, Excel etc. as desired.

Tracks

CandiMeth produced four different tracks from the differential methylation table input in the first step, of three different kinds (absolute methylation, relative differences in methylation (delta beta) and FDR-significant methylation difference, as shown in the example above for a cell line system.

Findings

The utility of the *CandiMeth* workflow may be best illustrated by some case studies.

Case Study 1: Application to array results from model systems

One straightforward use of *CandiMeth* which has found common use in our lab and among collaborators is to test a specific gene set, as illustrated by the *MIR* example above (Fig.2). To do this, the user only has to specify a list of the names of the genes they are interested in, together with the genome release, then upload a table containing differential methylation data. This can either be one generated by the bioinformatics team in-house, one which was

supplied, typically when array services are bought in, or one which was generated from publicly-available array data such as our dataset GSE90012 described previously [29] and used above.

Case Study 2: Application to EWAS study outputs

A major application of methylation array technology is in epigenome-wide association studies (EWAS). CandiMeth can provide a very useful tool for quickly examining in detail and quantifying methylation differences around candidate regions identified either by the R-based packages or from the literature. Fig.5 shows the application of this approach to an EWAS we have recently published containing data from 86 participants divided into 45 on placebo and 41 on folic acid treatment during trimesters 2 and 3 of pregnancy to assess the potential positive effects of prolonging this vitamin treatment beyond the currently-recommended periconception and first trimester periods [25]. Output differential methylation tables from RnBeads were used as input for CandiMeth, together with the names of the top candidate promoters reported earlier. This produced a collection of outputs (Fig.5A) including a set of tabular Results for the two groups Placebo and Treatment, as well as a set of Tracks. The latter included absolute mean beta, delta beta and an FDR track, although the latter returned the message "#No FDR significant sites" (not shown), often the case for EWAS if the sample set was small or the perturbation mild. Clicking through to the tabular results (Fig.5B) showed tables indicating the number of probes present at each promoter and mean methylation, revealing for example that median methylation at the *CES1* promoter is 2.5% lower in folic acid-treated participants than placebo (666.14 - 641.1=25/1000=0.025 or 2.5%).

Examination of the CandiMeth Tracks (Fig.5C) was however also informative here. This BedGraph track type is set by default to scale to the maximum loss and gain on visualisation, so that when the UCSC browser is opened on a genomic region of interest, not only are the

maximum loss and gain shown, but the graph is scaled to these, meaning that even when small differences in methylation occur, as typically seen in epidemiological studies, the areas of the genome with the greatest changes can be easily identified at a glance. In-house testing has found delta-beta tracks to be particularly useful, as it can easily be seen if a feature contains any probes with methylation differences between samples big enough to assess by other means – e.g. pyrosequencing can accurately assess differences in methylation greater than 5%. It can be easily seen from the delta beta (track 3) that the biggest loss of methylation was 7% (-0.071). The clustering of sites losing methylation at the promoter is also striking (boxed in green) compared to the rest of the gene, suggestive of a step-change in methylation at this important regulatory element rather than a point source. The seamless integration of BLAT [30] meant that designing primers to verify methylation changes could be done very intuitively and the area covered by the assay mapped against the methylation data to confirm that the assay could confirm methylation levels at the exact same location (Fig.5C track 4 “Pyro”).

It was also seen from the absolute methylation levels in the samples (tracks 1,2, values for promoters given in Fig.5B) that loss of methylation at the CES1 promoter occurred against a background of high methylation at this region, which suggested this control element is normally methylated and silenced, a type which often responds to even small losses of methylation. Additional data to corroborate this could be obtained by examining chromatin state data available through the ChomHMM track in UCSC (Fig.5 track 6) which showed that the promoter falls into the “poised promoter” category (colour-coded pink) and is regulated in part by polycomb-group proteins (grey shading). A low likelihood of SNPs at the pyroassay region could be confirmed by examination of the Common SNPs dataset (Fig.5C track 7) and individual CpGs labelled by searching using the UCSC query window, and their status in other public datasets highlighted if desired (Fig.5C, track 8). Thus CandiMeth allowed quick

examination of candidate regions, quantification of differences specifically at these, the assessment of sites which could be verified in the lab, exclusion of confounding SNPs, eased assay design and gave additional valuable insights through mining of UCSC datasets using only a few simple inputs and no coding.

Case Study 3: Analysis of methylation at genomic repeats such as LINE1

Many studies looking for epigenetic changes also try to assess DNA methylation outside of the coding regions. One common approach is to assess methylation at a highly-repetitive interspersed repeat such as LINE1, which is found scattered throughout the genome at ~500,000 copies, so in theory sampling methylation across many locations. This normally has to be done using a separate wet-lab assay such as pyrosequencing, since the 450K and EPIC arrays are designed to cover genes and their associated control elements, not repetitive DNA. However, as has been noted elsewhere [29 , 30], a substantial number of probes on the arrays, particularly the EPIC, nevertheless fall within repeats such as LINEs and SINEs. Taking advantage of this, we parsed data from the RepeatMasker track on UCSC to allow mapping and quantification of methylation at the major repeat classes using array data (Fig.6A). By simply listing the categories of repeat given by RepeatMasker (as in Suppl. Table 4), it is possible to obtain summary statistics indicating the numbers of probes overlapping the respective elements, together with median methylation etc. from any differential methylation table, in this case from our experiment comparing WT and DNMT1-deficient cell lines (Fig.6B). It can be seen from the tables that very substantial numbers of probes on the EPIC map to the various repeat classes, with ~20,000 probes in LINE elements spread across the genome, and equal numbers in SINE elements, with satellite repeats near centromeres showing the lowest coverage at ~1000. The summary data was exported to Excel and graphed to highlight where the greatest differences lay (Fig.6C), which showed that satellite sequences appear to be most demethylated on average, with notable decreases at LINE and

long terminal repeat (LTR)-containing elements too, which would include endogenous retroviruses for example, whereas low complexity and simple repeats show almost no changes, despite good probe coverage (Fig.6B). Thus CandiMeth allowed straightforward assessment of repeat methylation across the genome without the need for wet-lab analysis, and gave novel insights into the differential effects of DNMT1 loss on individual repetitive DNA classes.

Case Study 4: Analysis of methylation changes seen at a large complex gene locus in multiple samples using parallel processing in CandiMeth

A powerful feature of CandiMeth is the ability to process data from multiple differential methylation analyses at once. To illustrate this, we took three sets of comparisons between the independently-derived DNMT1 knockdown cell lines described earlier (d8, d10 and d16), each of which had been compared to the parental WT cell line and processed them simultaneously. In our earlier publication [24] we had found differences between the variable A and B classes and the variable C class of exons at the important neurodevelopmental gene cluster Protocadherin β (*PCDHB*), with the A&B classes showing severe loss of methylation but no change at the C class. This highlighted differences between these classes, which 1) indicate a hyper-dependence on DNMT1 for maintenance of methylation levels and 2) a potential difference in methylation-dependence which may track with allele usage, since the A&B classes show monoallelic expression but not the C class. Here, we wished to examine the neighbouring *PCDHG* locus which has a similar structure and see if the same effect could be seen there.

We therefore generated a candidate region list containing the names of the gamma cluster genes and input this as our candidate feature list input to *CandiMeth*, together with the three differential methylation tables from *RnBeads* (d8 vs WT, d10 vs WT, d16 vs WT). All three sets are processed at once (Fig.7A, left) and give as outputs data on absolute methylation

levels in each KD line as well as the WT parental line (which will not vary), from which summary tables were derived specific to the *PCDHG* exons- example data for one A and one C exon in each cell line only is shown (Fig.7A, right). Interestingly, the summary statistics indicated that, while levels of methylation appeared to be decreased across A&B class variable exons at this locus too (e.g. *PCDHGA1* 63.8% median methylation in WT vs 50.9%, 55.1% and 46% in d8, d10 and d16, respectively), median methylation at C class variable exons appeared to be increasing rather than remaining constant (e.g. *PCDHGC3* 86.5% WT, 89.1%, 89.1% and 88.9% d8, d10 and d16).

CandiMeth additionally generated Tracks outputs including the full range of tracks for each input table (absolute methylation in WT and each KD, delta beta and FDR for each vs WT). In Fig.7C we show the differential methylation (delta beta) tracks, from which it appeared that methylation was largely lost across the region of the gene containing the A&B class variable exons (Fig.7C, region boxed in red), though some gains (blue peaks) could be seen particularly in the d10 track. Additionally, given the size of the region (~200kb) it cannot be assessed whether many of the probes lie in the introns rather than the exons themselves. For the C class exons (Fig.7C, blue box at right) most changes appeared to be gains (blue) though peak sizes were smaller and interspersed with some individual large losses in red. To resolve the exact nature of the changes seen, the tabular data (Fig.7A) was exported and median values across all A&B exons vs WT generated, converted back to β value to allow direct comparison to previous results [25] and plotted (Fig.7D). This clearly showed a general loss of methylation at A&B class exons in all three cell lines ($p<0.001$ vs WT by Kruskal-Wallis test), although the effect was least marked in the d10 cell line. When values were averaged in a similar fashion across the C class variable exons however (Fig.7E) we saw a clear gain of methylation in all three cell lines ($p<0.05$, K-W test). The reason this effect was not noted before is likely to be because our previous examination of the C class exons at PCDHG used

the FDR-significant probes only, and as can be seen the magnitude of the gains at the C class exons is much smaller than the losses at the A&B classes (compare scales in Fig.7D and E).

The analysis thus confirmed and extended observations from our previous study that the A&B class variable exons at the clustered protocadherin loci are hypersensitive to loss of DNMT1 across multiple independently-derived cell lines, suggesting a strong dependence on this enzyme for maintenance of epigenetic state at this important neurodevelopmental locus. Further, we have uncovered new evidence for differences between the A&B exons and the C exons, which may reflect divergent transcriptional control, or an increased transcription across the C class exons in response to loss of DNMT1, in line with observations that intragenic DNA methylation is associated with transcription at active loci [10 , 31]. In terms of *CandiMeth* functionality, the study highlights the ability of the workflow to process multiple comparisons in parallel and the value of being able to directly compare the visual outputs and the quantitative data where complex genetic loci are being examined, giving insights into the underlying biology.

Conclusions and Future Directions

CandiMeth provides a user-friendly non-computationally intensive method of candidate feature investigation. With a minimum of training and no coding, users of *CandiMeth* can set up and run quite advanced exploratory and confirmatory analyses and use the rich set of existing data in UCSC to formulate and test hypotheses regarding the methylation changes they are seeing.

In future versions, we hope to add support for further methylation processing pipelines and continue to grow the *CandiMeth* history with additional genomic data such as DNA hypersensitivity sites. In addition to the current pipeline, we also wish to make CandiMeth more intuitive via the creation of a Galaxy tool which would allow the pipeline to be

extended to whole genome bisulphite sequencing or RNA-seq data and would also allow further analysis options for those with a private instance of Galaxy.

Availability of supporting source code and requirements

Project Name: CandiMeth

Project home page: http://bit.do/canidmeth-github

Operating system: www.usegalaxy.org

License: GNU GPL

Availability of supporting data

Declarations

List of abbreviations

CGI – CpG Island

DMR – Differentially Methylated Region

FDR – False Discovery Rate

HGNC – Human Genome Nomenclature Committee

SNPs – Single Nucleotide Polymorphisms

RefSeq – Reference Sequence

UCSC – University of California Santa Cruz

Ethics approval and consent to participate

Not Applicable

Consent for publication

Not Applicable

Competing interests

The authors declare that they have no competing interests.

Funding

Work was funded by grants from the Medical Research Council (MR/J007773/1) and the ESRC/BBSRC (ES/N000323/1).

Authors' contributions

SJT generated and tested the workflow and accompanying datasets and drafted the MS; DL helped with Sed and workflow debugging; REI provided supervision and feedback on early versions; KP and SDZ provided guidance and comments; CPW designed the study, generated early versions of the workflow and co-wrote the MS: all authors commented on and approved the final version.

Acknowledgements

We are grateful to members of the CPW lab for beta-testing of the workflow and accompanying histories in Galaxy, and especially to Sarah Cairns for help with the Guide which accompanies the workflow on GitHub.

Bibliography

[1] D. Schübeler, “Function and information content of DNA methylation,” *Nature*, vol. 517, no. 7534, pp. 321–326, Jan. 2015.

[2] C. Beard, E. Li, and R. Jaenisch, “Loss of methylation activates Xist in somatic but not in embryonic cells.,” *Genes Dev.*, vol. 9, no. 19, pp. 2325–2334, Oct. 1995.

[3] A. M. Deaton and A. Bird, “CpG islands and the regulation of transcription.,” *Genes Dev.*, vol. 25, no. 10, pp. 1010–22, May 2011.

[4] F. Lyko, “The DNA methyltransferase family: a versatile toolkit for epigenetic regulation,” *Nat. Rev. Genet.*, vol. 19, no. 2, pp. 81–92, Oct. 2017.

[5] M. S. Bartolomei and A. C. Ferguson-Smith, “Mammalian Genomic Imprinting,” *Cold Spring Harb. Perspect. Biol.*, vol. 3, no. 7, pp. a002592–a002592, Jul. 2011.

[6] G. Mountoufaris, D. Canzio, C. L. Nwakeze, W. V. Chen, and T. Maniatis, “Writing, Reading, and Translating the Clustered Protocadherin Cell Surface Recognition Code for Neural Circuit Assembly,” *Annu. Rev. Cell Dev. Biol.*, vol. 34, no. 1, pp. 471–493, Oct. 2018.

[7] C. P. Walsh, J. R. Chaillet, and T. H. Bestor, “Transcription of IAP endogenous retroviruses is constrained by cytosine methylation,” *Nat. Genet.*, vol. 20, no. 2, pp. 116–117, Oct. 1998.

[8] G. L. Xu *et al.*, “Chromosome instability and immunodeficiency syndrome caused by mutations in a DNA methyltransferase gene,” *Nature*, vol. 402, no. 6758, pp. 187–191, Nov. 1999.

[9] R. E. Irwin, A. Thakur, K. M. O’ Neill, and C. P. Walsh, “5-Hydroxymethylation marks a class of neuronal gene regulated by intragenic methylcytosine levels,”

Genomics, vol. 104, no. 5, pp. 383–392, 2014.

[10] H. Wu *et al.*, “Dnmt3a-dependent nonpromoter DNA methylation facilitates transcription of neurogenic genes,” *Science (80-.).*, vol. 329, no. 5990, pp. 444–447, 2010.

[11] C. Sapienza and J.-P. Issa, “Diet, Nutrition, and Cancer Epigenetics,” *Annu. Rev. Nutr.*, vol. 36, no. 1, pp. 665–681, Jul. 2016.

[12] A. J. Stevens, J. J. Rucklidge, and M. A. Kennedy, “Epigenetics, nutrition and mental health. Is there a relationship?,” *Nutr. Neurosci.*, vol. 21, no. 9, pp. 602–613, Oct. 2018.

[13] M. Vaz *et al.*, “Chronic Cigarette Smoke-Induced Epigenomic Changes Precede Sensitization of Bronchial Epithelial Cells to Single-Step Transformation by KRAS Mutations.,” *Cancer Cell*, vol. 32, no. 3, p. 360–376.e6, 2017.

[14] Q. A. Abdul, B. P. Yu, H. Y. Chung, H. A. Jung, and J. S. Choi, “Epigenetic modifications of gene expression by lifestyle and environment,” *Arch. Pharm. Res.*, vol. 40, no. 11, pp. 1219–1237, Nov. 2017.

[15] M. Suzuki *et al.*, “Whole-genome bisulfite sequencing with improved accuracy and cost.,” *Genome Res.*, vol. 28, no. 9, pp. 1364–1371, Sep. 2018.

[16] M. Bibikova *et al.*, “High density DNA methylation array with single CpG site resolution.,” *Genomics*, vol. 98, no. 4, pp. 288–95, Oct. 2011.

[17] Y. Assenov, F. Müller, P. Lutsik, J. Walter, T. Lengauer, and C. Bock, “Comprehensive analysis of DNA methylation data with RnBeads.,” *Nat. Methods*, vol. 11, no. 11, pp. 1138–40, Nov. 2014.

[18] T. J. Morris *et al.*, “ChAMP: 450k Chip Analysis Methylation Pipeline,”

Bioinformatics, vol. 30, no. 3, pp. 428–430, Feb. 2014.

[19] Y. Tian *et al.*, “ChAMP: Updated methylation analysis pipeline for Illumina BeadChips,” *Bioinformatics*, vol. 33, no. 24, pp. 3982–3984, Dec. 2017.

[20] F. Müller *et al.*, “RnBeads 2.0: comprehensive analysis of DNA methylation data,” *Genome Biol.*, vol. 20, no. 1, p. 55, Dec. 2019.

[21] A. Subramanian *et al.*, “Gene set enrichment analysis: a knowledge-based approach for interpreting genome-wide expression profiles.,” *Proc. Natl. Acad. Sci. U. S. A.*, vol. 102, no. 43, pp. 15545–50, Oct. 2005.

[22] B. Giardine *et al.*, “Galaxy: a platform for interactive large-scale genome analysis.,” *Genome Res.*, vol. 15, no. 10, pp. 1451–5, Oct. 2005.

[23] C. A. Goble and D. C. De Roure, “myExperiment,” in *Proceedings of the 2nd workshop on Workflows in support of large-scale science - WORKS ’07*, 2007, pp. 1–2.

[24] K. M. O’Neill *et al.*, “Depletion of DNMT1 in differentiated human cells highlights key classes of sensitive genes and an interplay with polycomb repression,” *Epigenetics and Chromatin*, vol. 11, no. 1, 2018.

[25] R. E. Irwin *et al.*, “A randomized controlled trial of folic acid intervention in pregnancy highlights a putative methylation-regulated control element at ZFP57,” *Clin. Epigenetics*, vol. 11, no. 1, p. 31, Dec. 2019.

[26] B. Batut *et al.*, “Collections: Using dataset collection,” *galaxyproject.github.io*, 2017. [Online]. Available: https://galaxyproject.github.io/training-material/topics/galaxy-data-manipulation/tutorials/collections/tutorial.html. [Accessed: 05-Aug-2019].

[27] N. A. O’Leary *et al.*, “Reference sequence (RefSeq) database at NCBI: current status,

taxonomic expansion, and functional annotation,” *Nucleic Acids Res.*, vol. 44, no. D1, pp. D733–D745, Jan. 2016.

[28] S.-J. Thursby, “CandiMeth History,” 2019. [Online]. Available: https://is.gd/candiMeth_history/. [Accessed: 12-Aug-2019].

[29] K. M. O’Neill *et al.*, “Depletion of DNMT1 in differentiated human cells highlights key classes of sensitive genes and an interplay with polycomb repression,” *Epigenetics Chromatin*, vol. 11, no. 1, p. 12, Dec. 2018.

[30] W. J. Kent, “BLAT---The BLAST-Like Alignment Tool,” *Genome Res.*, vol. 12, no. 4, pp. 656–664, Mar. 2002.

[31] Y. Zheng *et al.*, “Prediction of genome-wide DNA methylation in repetitive elements,” *Nucleic Acids Res.*, vol. 45, no. 15, pp. 8697–8711, Sep. 2017.

[32] R. Pidsley *et al.*, “Critical evaluation of the Illumina MethylationEPIC BeadChip microarray for whole-genome DNA methylation profiling,” *Genome Biol.*, vol. 17, no. 1, p. 208, Dec. 2016.

[33] R. D. Hawkins *et al.*, “Distinct epigenomic landscapes of pluripotent and lineage-committed human cells,” *Cell Stem Cell*, vol. 6, no. 5, pp. 479–491, May 2010.

[34] B. Gruening, “Sed.” GitHub, Freiburg, p. 1, 2016.

[35] P. Mabon, J. Chilton, and M. Van Den Beek, “Merge Collections.” GitHub, Pennsylvania, p. 1, 2018.

[36] M. Van Den Beek and J. Chilton, “Relabel List Identifiers.” GitHub, Pennsylvania, p. 1, 2018.

[37] P. Mabon, “Collapse Collection.” GitHub, Winnipeg, p. 1, 2017.

[38] N. Coraor *et al.*, “Join Two Datasets.” GitHub, Pennsylvania, p. 1, 2009.

[39] H. Gamaleldin and B. Gruening, “Cut.” GitHub, Freiburg, p. 1, 2016.

[40] B. Gruening and H. Gamaleldin, “Unique.” GitHub, Freiburg, p. 1, 2015.

[41] B. Gruening, H. Gamaleldin, and N. Soranzo, “Join.” GitHub, Freiburg, p. 1, 2015.

[42] D. Baker *et al.*, “Group.” GitHub, Atlanta, p. 1, 2011.

[43] G. Von Kuster, P. Cock, N. Soranzo, J. Taylor, and D. Baker, “Add Column.” GitHub, p. 1, 2018.

[44] M. Van Den Beek, “Compute Expression on Every Row.” 2017.

Tables

Table 1 List of Galaxy tools used

TOOL NAME	TOOL ID	VERSION	REFERENCE
**TEXT REFORMATTING**	sed_stream_editor	0.0.1	[34]
**MERGE COLLECTIONS**	__MERGE_COLLECTION__	1.0.0	[35]
**RELABEL LIST IDENTIFIERS**	__RELABEL_FROM_FILE__	1.0.0	[36]
**COLLAPSE COLLECTION**	Collapse Collection	4.0.0	[37]
**JOIN TWO DATASETS**	join1	2.1.2	[38]
**CUT**	tp_cut_tool	1.1.0	[39]
**UNIQUE**	tp_sorted_uniq	1.1.0	[40]
**JOIN**	tp_easyjoin_tool	1.0.0	[41]
**GROUP**	Grouping1	2.1.2	[42]
**ADD COLUMN**	addValue	1.0.0	[43]
**COMPUTE**	Add_a_column1	1.2.1	[44]

Figures

Figure 1 Overview of *CandiMeth* workflow

When the CandiMeth workflow is started the user needs to specify as Inputs (left): 1) the type of R package used; 2) the methylation data, normally in the form of a differential methylation table generated by the package 3) a list of genes to be analysed and 4) a human genome build to match the data to. The methylation data are then mapped (centre left) to the genome and sites overlapping features of interest analysed (centre right). The data are then output quantitatively as Results and visually as Tracks (right).

Figure 2 Case Study 1: analysing new genes in a published dataset

Our previously-published dataset GSE90012 using *RnBeads* to compare methylation levels in cells deficient in DNA methyltransferase 1 (d8 KD) with wild type (WT) was reanalysed for methylation levels at microRNA (*MIR*) using *CandiMeth*. **(A)** The workflow generated two grouped sets of outputs (white boxes at left) on completion, 'Mir Cluster | hg_19_all | CandiMeth Results' containing links to the tabular quantitative data and 'CandiMeth Tracks' with links to the tracks on UCSC **(B)** *CandiMeth* Results box expanded: a separate dataset for each cell line is generated showing the list of candidates, probe coverage, median and a variety of other statistics for each gene analysed (top 5 rows only shown). **(C)** *CandiMeth* Tracks: UCSC genome browser view, accessible via the eye symbols on the *Galaxy* history shown in (A): (From the top down) Scale bar, size of region in kilobases of DNA; chr1, chromosome number and exact coordinates from the *hg19* genome build. *(1-4)* CandiMeth tracks: *1) Mean_beta_d8R*, absolute methylation track reflecting array output going from 1, no methylation, to 1000, fully methylated e.g. 811= 81.1% , max and min indicated at left *2) Mean_beta_d8R*, absolute methylation in WT *3) delta_d8vsWT*, a differential methylation track showing proportional change going from -1.0 (100% loss, red) to +1.0 (100% gain, blue) e.g -0.155 = loss of 15.5% compared to WT; *4) FDR_D8,* a significance score track

showing only those sites whose differential methylation meets the cut-off criteria of a 0.05 false discovery rate. *(5-7)* Examples of some of the tracks available through the UCSC genome browser which can be aligned and directly compared to *CandiMeth* tracks: *5) HAIB Methyl450*, data on comparative methylation from ENCODE projects; *6) Pyro*, the *Blat* tool in UCSC, which can be used to find primers for pyroassays to cover one or multiple CG *7) RefSeq* track, showing the location of the top two MIR from (B) above.

Figure 3: User Interface for the workflow

Screenshot of the workflow start window (middle pane) which appears on right-clicking >CandiMeth>Run. The right-hand side (RHS) shows the *CandiMeth* starting history, where preloaded data used with the workflow can be found, together with any user-uploaded datasets. Galaxy tools (LHS) are not used. For the workflow the user chooses whether to save results to a new history (recommended), then specifies 1) which *R* package was used to pre-process the data e.g. *RnBeads*; 2) the dataset collection table of pre-processed data- available sets will appear in the drop-down menu; 3) a list of the genes/other features of interest to analyse 4) the reference genome to be mapped to e.g. *hg19.* Once all four have been decided, the user clicks on the blue "Run workflow" button at top right to initiate a run.

Figure 4: Galaxy Workflow Editor View of *CandiMeth*

Detailed view of the workflow using the editing tool in Galaxy. Steps in the workflow have been grouped for clarity. *(1) Inputs*: here the user indicates which R package was used to analyse their array using the keywords '*Rnbeads*', '*Champ*' or 'Custom' (1.1) the differential methylation table resulting from this *R* package (1.2), genomic features they wish to analyse (1.3) and specifies the desired genome build (1.4). *(2) Standardising the input data*: Using the *R* package information in 1.1 and the differential methylation table in 1.2, *CandiMeth* generates a table showing the chromosome location, start, end, mean methylation in the control and experimental groups, the difference between these groups and the FDR adjusted p

value. *3) Track generation*: maps the data on absolute as well as differential methylation from the table to the genome build *(4) Track naming*: generates unambiguous labels for each type of track. *(5) Merging of tracks and names*: this ensures logical labelling and grouping of tracks. *(6) Feature mapping*: this maps the specific features to the same genome build. *(7) Compilation of feature methylation*: this parses the data in the tracks to only examine the features of interest *(8) Output Tables*: these contain summary statistics on the features of interest and are one major output *(9) Output Tracks*: the user can also see the mapping which the summary stats are based on, which allows them to see areas adjacent to the features of interest, and overlay other UCSC tracks, as well as use tools such as Blat.

Figure 5: Case Study 2: Using *CandiMeth* to mine EWAS data

Example data from an EWAS dataset comparing 45 placebo and 41 treated samples from a randomised control trial of a folic acid intervention during second and third trimester of pregnancy. **(A)** Output Results and Tracks from the workflow when the *RnBeads* differential methylation table and a list of the top-ranked differentially methylated promoters were used as inputs. **(B)** Summary statistics generated by the workflow indicates the number of probes and methylation values (from 1-1000) for the top promoters **(C)** Tracks view for the CES1 locus showing the absolute levels of methylation (tracks 1,2) as well as the most differentially-methylated probes (track 3) located at the promoter (boxed in green). Comparison to ChromHMM data in UCSC (track 6) shows this to be a poised promoter (pink). Identification of individual CG (numbered in track 8) facilitated the design of a pyrosequencing assay (track 4) covering the CG to be validated in the lab.

Figure 6: Case Study 3: Analysis of *LINE*s and *SINE*s

Use of CandiMeth to give an overview of methylation at repetitive elements. **(A)** Data on repeat location and type from the RepeatMasker track on UCSC has been parsed and made available through the workflow: users can therefore simply type in the name(s) of a class of

repeats as a query. **(B)** Example outputs showing probe coverage of repeats on the EPIC array and methylation statistics for each repeat class in a DNMT1 knockdown cell line (d16 KD) versus WT. **(C)** Tables from B were exported median methylation levels converted to % and then graphed to highlight differences between the two cell lines: decreases in methylation are seen at some (LINE, SINE, satellite) but not other repeats (low complexity, simple).

Figure 7: Case Study 4: Parsing data from a complex gene locus using parallel processing

Analysis of methylation at variable exons in the large (~200kb) clustered *Protocadherin γ* (*PCDHG*) locus on human chromosome 5. **(A)** Results: changes at the variable exons across three independent cell lines deficient in DNMT1 (d8, d10, d16) were scored using the *RnBeads* tables comparing each to WT as input, together with a list of variable exon names. Example quantitative outputs are shown at right for WT and knockdown (KD) cells. **(B)** Tracks: part of the output set of tracks is shown, which included mean beta, differential methylation and FDR significant sites (not shown) for all cell lines, generated simultaneously in one run. **(C)** The UCSC browser view available by following the links in (B). The region covering the A and B class variable exons (boxed in red) appears to show more loss of methylation while the C class (boxed in blue) appear to show predominantly gains, however this is not exclusive and many probes lie between exons. **(D, E)** Data on probes which lie solely in exons and not introns, obtained through Results (B), was exported and grouped as indicated. The numbers were then converted back into beta values and graphed. This confirmed that methylation was lost on average at the A and B class exons, while the C class predominantly gained methylation. *** $p<0.001$, *, $p<0.05$ by Kruskal-Wallis test.

Supplementary Materials

Supplementary File 1: *CandiMeth* Tutorial

Guide to the initial steps for setting up in Galaxy and importing the *CandiMeth* workflow and example files.

Supplementary Table 1: Example Differential Methylation Table generated by *RnBeads* from GSE90012 for input to *CandiMeth*

Table comparing wild type hTERT1604 human fibroblasts (WT) and a clonally-derived daughter cell line with depleted levels of DNA methyltransferase 1 (d8) from GEO database entry GSE90012, used as Input 2 to *CandiMeth* in Case Study 1 (Fig.2).

Supplementary Table 2: MIR gene list used to query data from GSE90012

List of human microRNA genes (*MIR*) used as Input 3 to *CandiMeth* in Case Study 1.

Supplementary Table 3: Methylation summary for MIR genes derived by CandiMeth

Full table of Results for *MIR* methylation in GSE90012 WT vs DNMT1-depleted (d8) cells given as output from *CandiMeth* (Fig.2B).

Supplementary Table 4: Classes of repetitive DNA sequence which can be analysed

List of repetitive DNA classes as given by *RepeatMasker* and which can be used to as Input 3 by *CandiMeth* to query datasets, as in Case Study 3 (Fig.6).

Supplementary Table 5: Example Differential Methylation Table from ChAMP

Example data in *ChAMP* format for use in tutorial as Input 2 in *CandiMeth.*

Figure 1

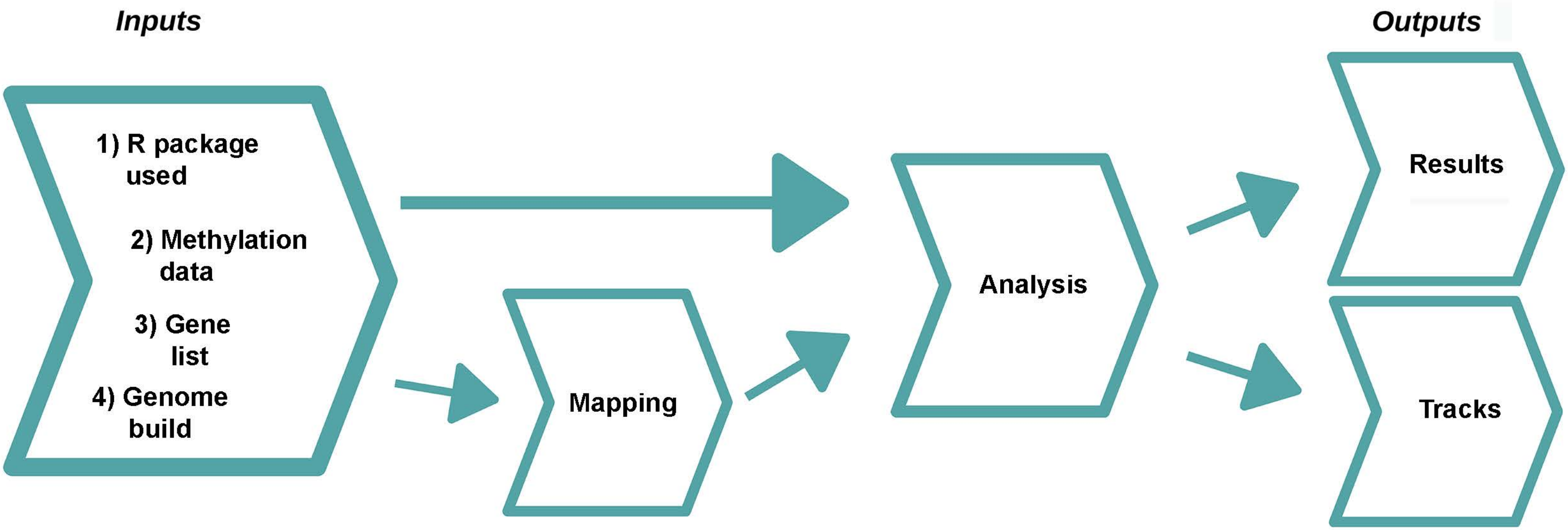
Inputs
Outputs
1) R package used
2) Methylation data
3) Gene list
4) Genome build
Mapping
Analysis
Results
Tracks

Figure 2

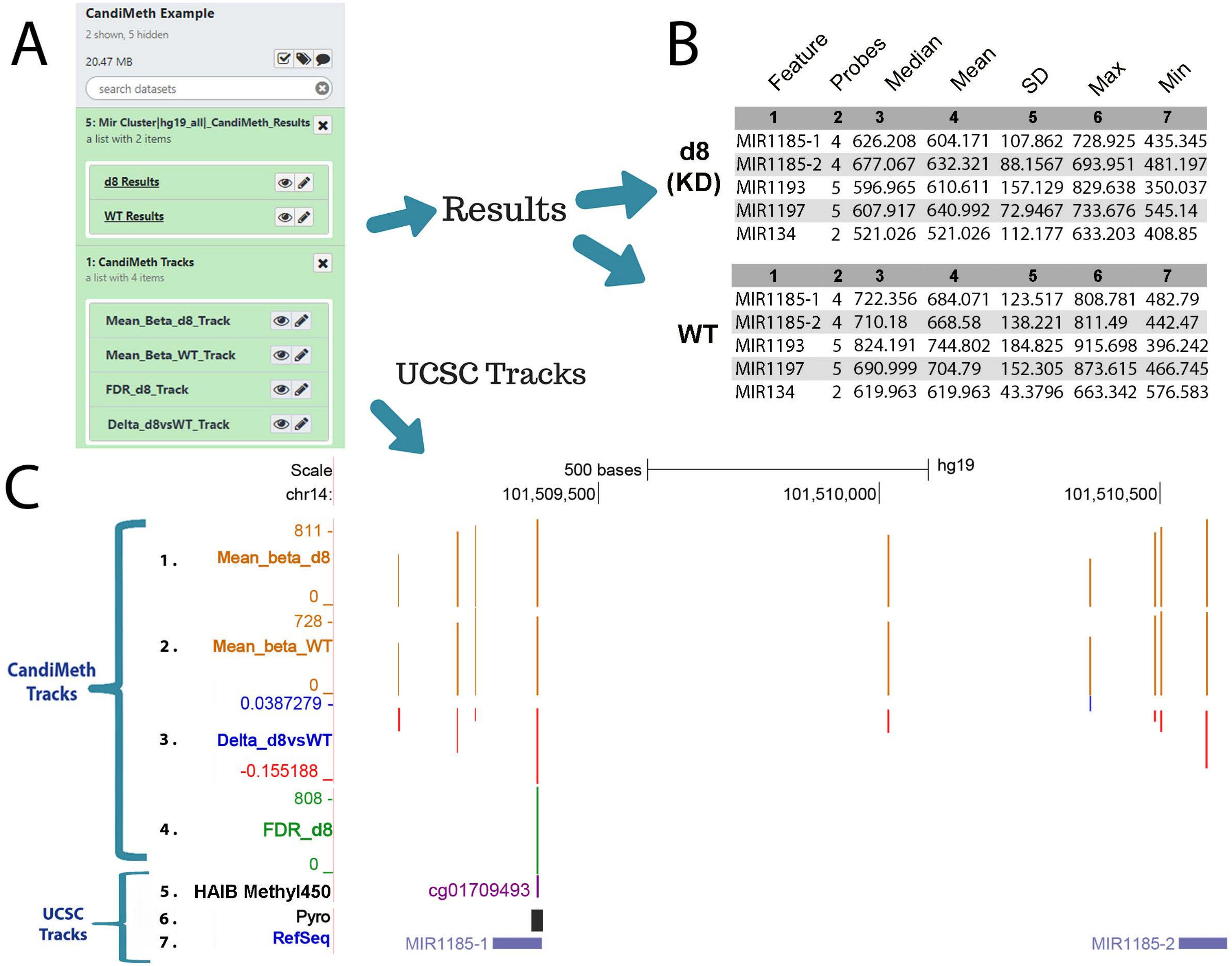

d8 (KD)

1	2	3	4	5	6	7
MIR1185-1	4	626.208	604.171	107.862	728.925	435.345
MIR1185-2	4	677.067	632.321	88.1567	693.951	481.197
MIR1193	5	596.965	610.611	157.129	829.638	350.037
MIR1197	5	607.917	640.992	72.9467	733.676	545.14
MIR134	2	521.026	521.026	112.177	633.203	408.85

WT

1	2	3	4	5	6	7
MIR1185-1	4	722.356	684.071	123.517	808.781	482.79
MIR1185-2	4	710.18	668.58	138.221	811.49	442.47
MIR1193	5	824.191	744.802	184.825	915.698	396.242
MIR1197	5	690.999	704.79	152.305	873.615	466.745
MIR134	2	619.963	619.963	43.3796	663.342	576.583

Figure 3

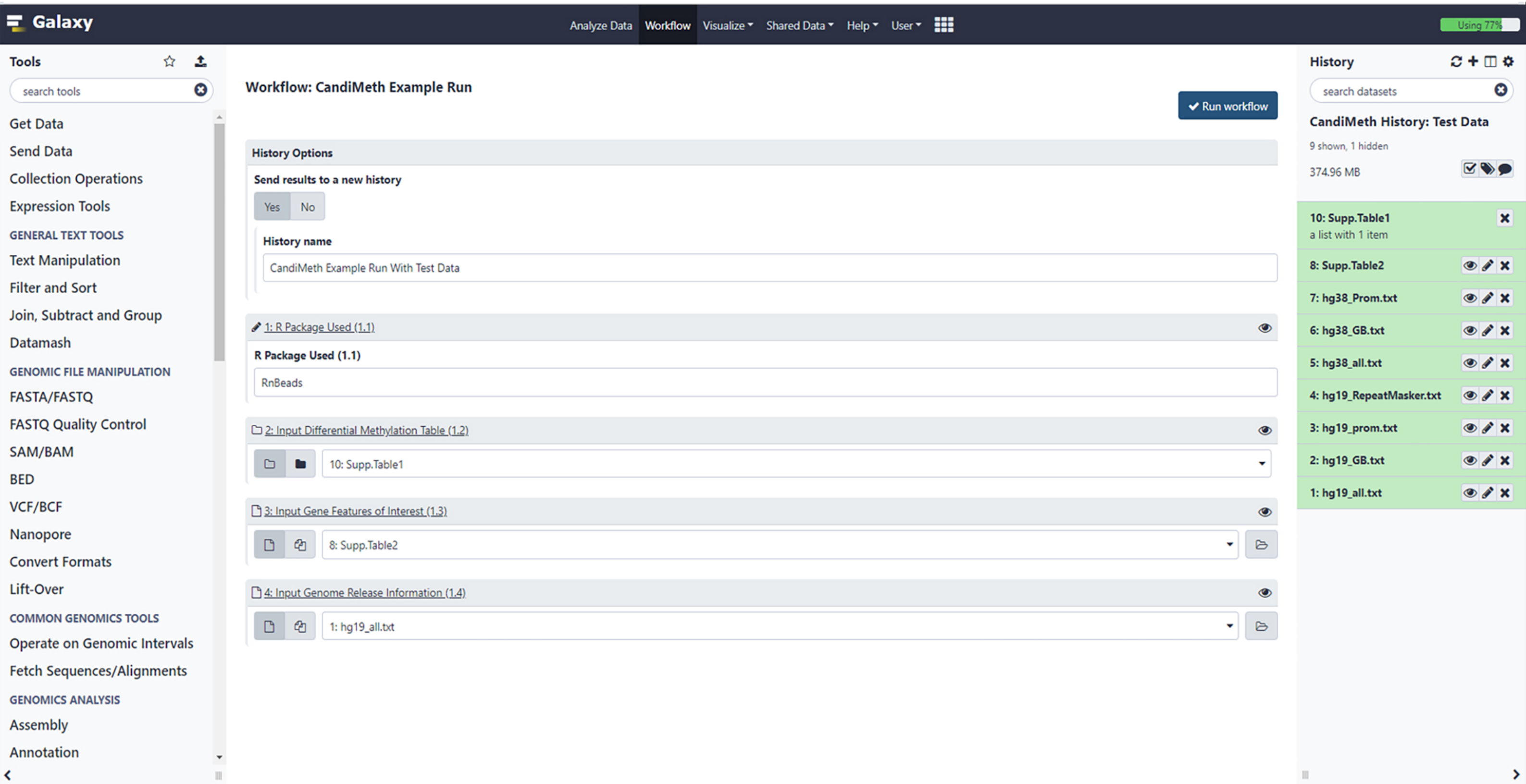

Galaxy
Analyze Data
Workflow
Visualize
Shared Data
Help
User
Using 77%
Tools
search tools
Get Data
Send Data
Collection Operations
Expression Tools
GENERAL TEXT TOOLS
Text Manipulation
Filter and Sort
Join, Subtract and Group
Datamash
GENOMIC FILE MANIPULATION
FASTA/FASTQ
FASTQ Quality Control
SAM/BAM
BED
VCF/BCF
Nanopore
Convert Formats
Lift-Over
COMMON GENOMICS TOOLS
Operate on Genomic Intervals
Fetch Sequences/Alignments
GENOMICS ANALYSIS
Assembly
Annotation
Workflow: CandiMeth Example Run
Run workflow
History Options
Send results to a new history
Yes
No
History name
CandiMeth Example Run With Test Data
1: R Package Used (1.1)
R Package Used (1.1)
RnBeads
2: Input Differential Methylation Table (1.2)
10: Supp.Table1
3: Input Gene Features of Interest (1.3)
8: Supp.Table2
4: Input Genome Release Information (1.4)
1: hg19_all.txt
History
search datasets
CandiMeth History: Test Data
9 shown, 1 hidden
374.96 MB
10: Supp.Table1
a list with 1 item
8: Supp.Table2
7: hg38_Prom.txt
6: hg38_GB.txt
5: hg38_all.txt
4: hg19_RepeatMasker.txt
3: hg19_prom.txt
2: hg19_GB.txt
1: hg19_all.txt

Figure 4

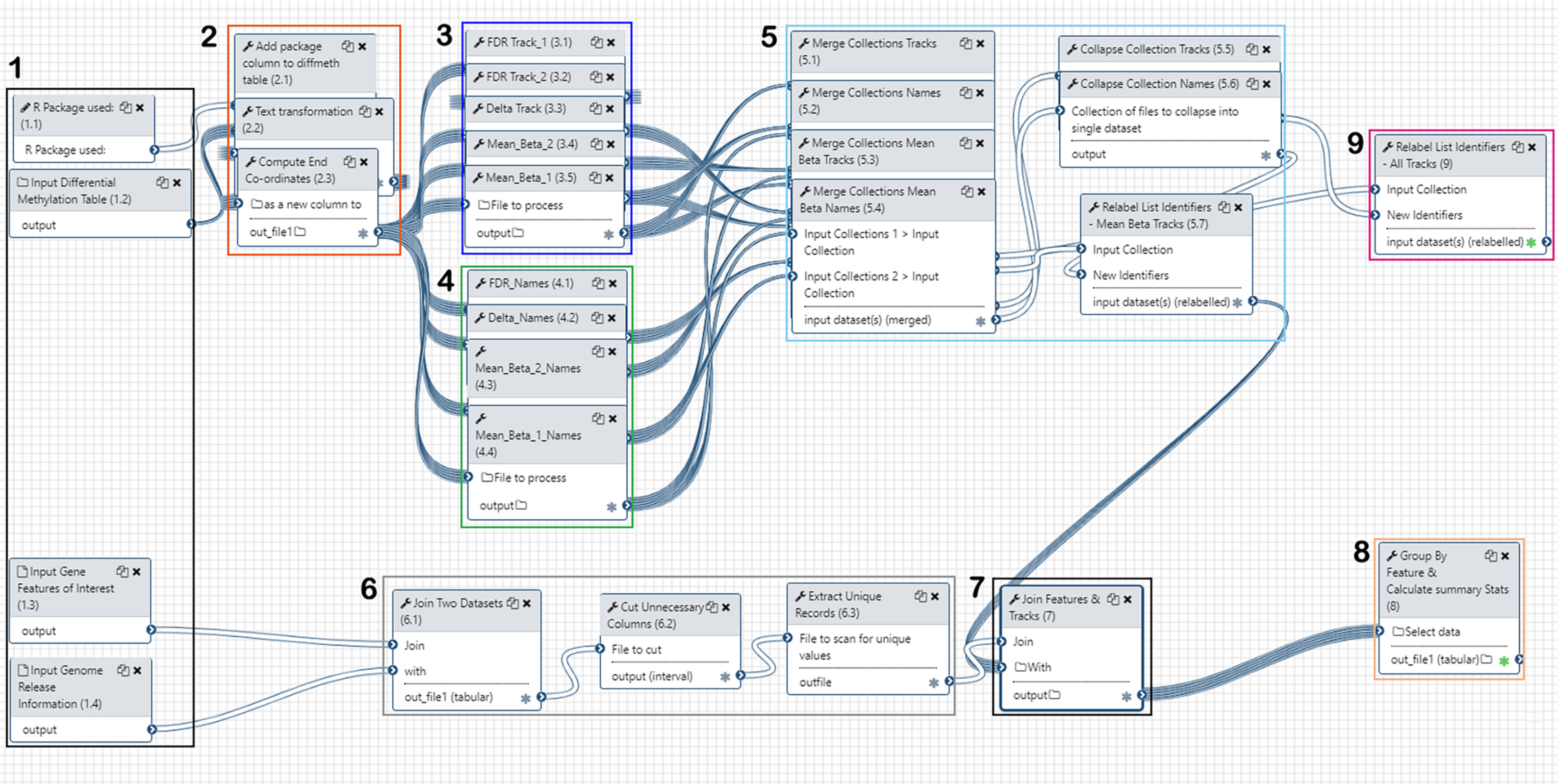
1
R Package used: (1.1)
R Package used:
Input Differential Methylation Table (1.2)
output
Input Gene Features of Interest (1.3)
output
Input Genome Release Information (1.4)
output
2
Add package column to diffmeth table (2.1)
Text transformation (2.2)
Compute End Co-ordinates (2.3)
as a new column to
out_file1
3
FDR Track_1 (3.1)
FDR Track_2 (3.2)
Delta Track (3.3)
Mean_Beta_2 (3.4)
Mean_Beta_1 (3.5)
File to process
output
4
FDR_Names (4.1)
Delta_Names (4.2)
Mean_Beta_2_Names (4.3)
Mean_Beta_1_Names (4.4)
File to process
output
5
Merge Collections Tracks (5.1)
Merge Collections Names (5.2)
Merge Collections Mean Beta Tracks (5.3)
Merge Collections Mean Beta Names (5.4)
Input Collections 1 > Input Collection
Input Collections 2 > Input Collection
input dataset(s) (merged)
Collapse Collection Tracks (5.5)
Collapse Collection Names (5.6)
Collection of files to collapse into single dataset
output
Relabel List Identifiers - Mean Beta Tracks (5.7)
Input Collection
New Identifiers
input dataset(s) (relabelled)
9
Relabel List Identifiers - All Tracks (9)
Input Collection
New Identifiers
input dataset(s) (relabelled)
6
Join Two Datasets (6.1)
Join
with
out_file1 (tabular)
Cut Unnecessary Columns (6.2)
File to cut
output (interval)
Extract Unique Records (6.3)
File to scan for unique values
outfile
7
Join Features & Tracks (7)
Join
With
output
8
Group By Feature & Calculate summary Stats (8)
Select data
out_file1 (tabular)

Figure 5

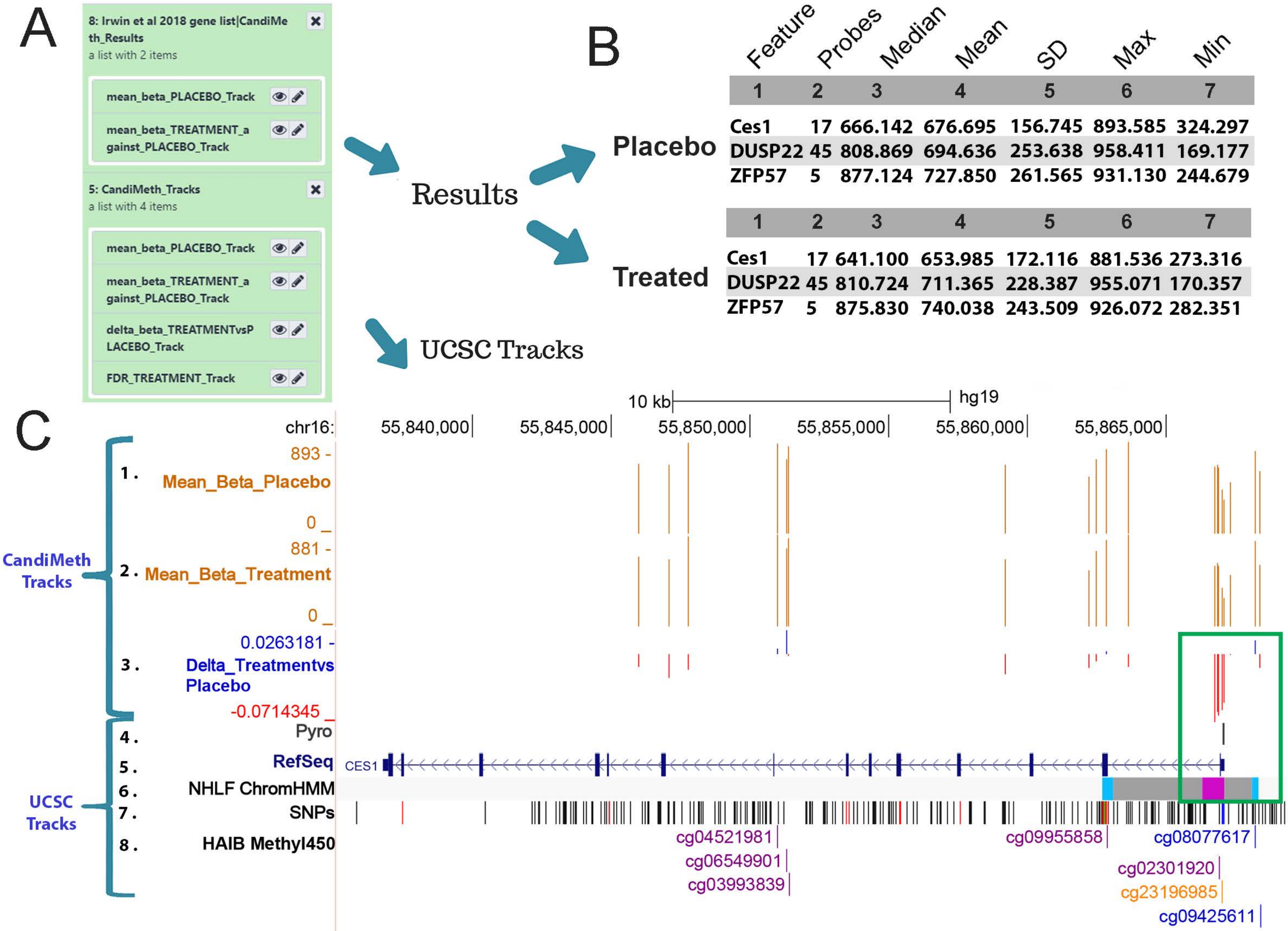
A
8: Irwin et al 2018 gene list|CandiMeth_Results
a list with 2 items
mean_beta_PLACEBO_Track
mean_beta_TREATMENT_against_PLACEBO_Track
5: CandiMeth_Tracks
a list with 4 items
mean_beta_PLACEBO_Track
mean_beta_TREATMENT_against_PLACEBO_Track
delta_beta_TREATMENTvsPLACEBO_Track
FDR_TREATMENT_Track
Results
UCSC Tracks
B
Feature Probes Median Mean SD Max Min
1 2 3 4 5 6 7
Placebo
Ces1 17 666.142 676.695 156.745 893.585 324.297
DUSP22 45 808.869 694.636 253.638 958.411 169.177
ZFP57 5 877.124 727.850 261.565 931.130 244.679
1 2 3 4 5 6 7
Treated
Ces1 17 641.100 653.985 172.116 881.536 273.316
DUSP22 45 810.724 711.365 228.387 955.071 170.357
ZFP57 5 875.830 740.038 243.509 926.072 282.351
C
10 kb
hg19
chr16:
55,840,000
55,845,000
55,850,000
55,855,000
55,860,000
55,865,000
CandiMeth Tracks
1. Mean_Beta_Placebo
893 -
0 _
2. Mean_Beta_Treatment
881 -
0 _
3. Delta_Treatmentvs Placebo
0.0263181 -
-0.0714345 _
UCSC Tracks
4. Pyro
5. RefSeq
CES1
6. NHLF ChromHMM
7. SNPs
8. HAIB Methyl450
cg04521981
cg06549901
cg03993839
cg09955858
cg08077617
cg02301920
cg23196985
cg09425611

Figure 6

A

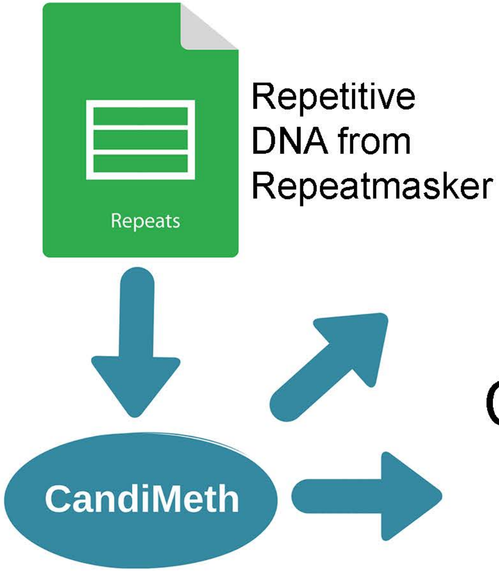

B

	Feature 1	Probes 2	Median 3	Mean 4	SD 5	Max 6	Min 7
WT	DNA	6603	570.000	333.001	495.235	13.000	952.000
	Line	19058	558.000	332.946	497.002	13.000	962.000
	Low_complexity	4232	69.000	248.196	190.519	13.000	947.000
	LTR	16227	511.000	313.360	478.816	15.000	953.000
	Satellite	1247	380.000	265.515	400.252	21.000	923.000
	Simple_Repeat	2951	101.000	295.152	274.33	17.000	951.000
	SINE	22399	601.000	308.351	523.311	16.000	955.000
d16 (KD)	DNA	6625	501.307	344.879	459.620	13.602	959.497
	Line	19110	489.348	343.962	457.704	13.312	965.908
	Low_complexity	4230	62.8057	243.318	173.712	17.177	951.222
	LTR	16258	427.397	322.464	430.180	14.879	957.649
	Satellite	1250	226.824	259.389	317.455	18.447	924.004
	Simple_Repeat	2959	83.7106	288.122	247.226	17.394	950.826
	SINE	22444	549.042	322.171	489.615	12.421	957.278

C

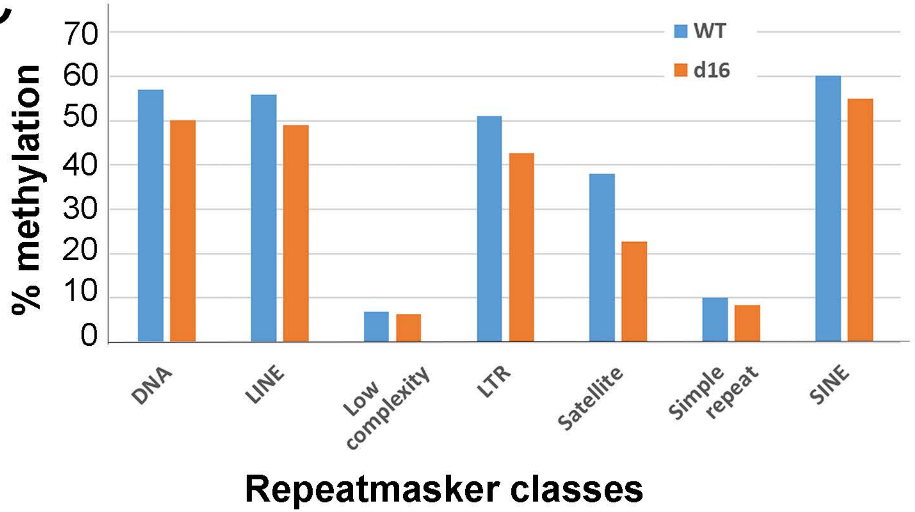

Figure 7

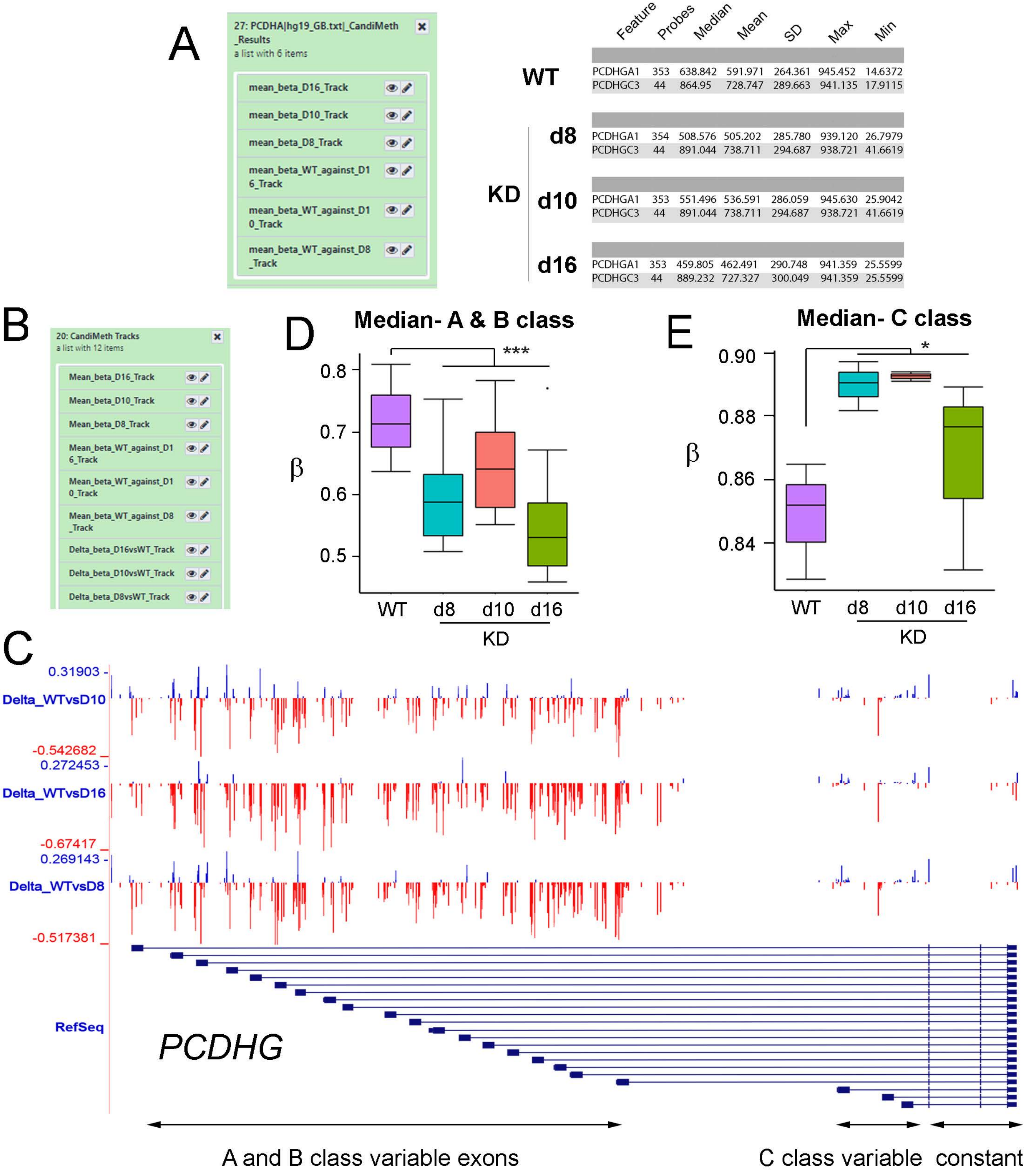
A
27: PCDHA|hg19_GB.txt|_CandiMeth _Results
a list with 6 items
mean_beta_D16_Track
mean_beta_D10_Track
mean_beta_D8_Track
mean_beta_WT_against_D1 6_Track
mean_beta_WT_against_D1 0_Track
mean_beta_WT_against_D8 _Track
Feature Probes Median Mean SD Max Min
WT
PCDHGA1 353 638.842 591.971 264.361 945.452 14.6372
PCDHGC3 44 864.95 728.747 289.663 941.135 17.9115
KD
d8
PCDHGA1 354 508.576 505.202 285.780 939.120 26.7979
PCDHGC3 44 891.044 738.711 294.687 938.721 41.6619
d10
PCDHGA1 353 551.496 536.591 286.059 945.630 25.9042
PCDHGC3 44 891.044 738.711 294.687 938.721 41.6619
d16
PCDHGA1 353 459.805 462.491 290.748 941.359 25.5599
PCDHGC3 44 889.232 727.327 300.049 941.359 25.5599
B
20: CandiMeth Tracks
a list with 12 items
Mean_beta_D16_Track
Mean_beta_D10_Track
Mean_beta_D8_Track
Mean_beta_WT_against_D1 6_Track
Mean_beta_WT_against_D1 0_Track
Mean_beta_WT_against_D8 _Track
Delta_beta_D16vsWT_Track
Delta_beta_D10vsWT_Track
Delta_beta_D8vsWT_Track
D
Median- A & B class
***
β
0.8
0.7
0.6
0.5
WT d8 d10 d16
KD
E
Median- C class
*
β
0.90
0.88
0.86
0.84
WT d8 d10 d16
KD
C
0.31903
Delta_WTvsD10
-0.542682
0.272453
Delta_WTvsD16
-0.67417
0.269143
Delta_WTvsD8
-0.517381
RefSeq
PCDHG
A and B class variable exons
C class variable
constant
