# Supplementary material for: CandiMeth: Powerful yet simple visualization and quantification of DNA methylation at candidate genes
Source: Gigascience. 2020 Jun 22;9(6):giaa066. doi: 10.1093/gigascience/giaa066 (PMC7307318; doi:10.1093/gigascience/giaa066)
Supplement: giaa066_Supplemental_Files [file giaa066_supplemental_files.zip › CandiMeth User Guide for GitHubv3.pdf]

CandiMeth User Guide

Contents

1. Overview: where to start 2
2. Getting set up 3
3. Using CandiMeth with the sample data provided 6
  3.1 Tutorial with the example data provided 6
    3.1.1 Inputs and starting the run 6
    3.1.2 Overview of outputs from the microRNA analysis 8
    3.1.3 Working with the output Results tables 9
    3.1.4 Graphing data directly in Galaxy 10
    3.1.5 Exporting data from Galaxy to Excel 11
    3.1.6 Working with output Tracks 14
    3.1.7 Exporting browser views as graphics files 20
    3.1.8 Quantifying methylation in different parts of the gene 22
  3.2 Looking at a new set of genes in the current methylation array dataset 23
  3.3 Looking at repetitive DNA elements such as LINES and ERV 25
  3.4 Using ChAMP-generated methylation data 27
4. Uploading and working with your own differential methylation data 28
  4.1 Locating data files in RnBeads 28
  4.2 Locating data files in ChAMP 30
  4.3 Uploading your data files to Galaxy 31
Appendix 1. Primer for those unfamiliar with the UCSC genome browser 33
Appendix 2. Quick guide to the Galaxy web environment 36
Appendix 3. Importing CandiMeth to a custom Galaxy instance 37

1. Overview: where to start

CandiMeth is aimed primarily at wet-lab biologists working in the general area of biomedical sciences who have access to DNA methylation data and want to look at methylation levels at specific candidate genes or regions, but are not adept at coding or programming.

Most commonly, the end-user of CandiMeth would have a report generated in html by one of the two main DNA methylation array processing pipelines RnBeads or ChAMP, which will give overviews of the data such as PCA analysis, graphs etc and then links to the processed data in the form of differential methylation tables showing beta values from the arrays, differences in methylation between samples and p values.

If you have tables of differential methylation values from RnBeads or ChAMP and want to look at specific genes then CandiMeth is a suitable tool.

CandiMeth will allow you to visualise the methylation at your genes of interest, as well as quantify it. Although there are various options to allow you to visualise the methylation, we recommend the UCSC genome browser, as this has a wide range of extra data on the genes which can enrich and inform your analysis.

- For those not familiar with the UCSC browser, see the Primer (Appendix 1)

CandiMeth basically takes the differential methylation data output by the RnBeads or ChAMP pipelines and uses the Galaxy web-based bioinformatics platform to map the data to the human genome and to assess and quantify regions of interest.

- For those unfamiliar with the Galaxy environment, see the Quick guide (Appendix 2)

CandiMeth users do not need to be very adept with either UCSC browser or Galaxy for CandiMeth to work, as the interface is very simple and the outputs in terms of visualisation and quantification very straightforward. If you have used the UCSC browser before, and have looked over the Galaxy webpage, then you should be able to try CandiMeth with little prior preparation.

- For those comfortable with the UCSC browser and who understand the basic screen layout in Galaxy, you need to get set up with the CandiMeth workflow and example data (Section 2)
- After that, we recommend using CandiMeth the first time with the sample data provided (Section 3)
- Once CandiMeth is working on your computer, you can try uploading and working with your own data (Section 4)
- Some additional help is available to enable conversion of ChAMP outputs to Excel files for import into CandiMeth (Section 4.2)
- If you run Galaxy on a local server or have a customised version, we provide a guide to importing CandiMeth below (Appendix 3)

2. Getting set up

[Note: CandiMeth, Galaxy and UCSC work best in Chrome or Firefox rather than Explorer. If Chrome is not your default browser, cut and paste the web-addresses in brackets into your browser window instead.]

1) CandiMeth runs in the Galaxy on-line environment: you will therefore need a working Galaxy account, which can be created for free here (http://www.usegalaxy.org) - those not familiar with Galaxy are referred to the brief introduction below

   [Tip! It may take a couple of seconds for the hyperlink to open.]

2) Once you have an account, go to :- https://usegalaxy.org/u/sarajayne-thursby/h/candimeth-history and click on the + button at the top RHS of the page – this will create a History in your own Galaxy account containing the reference genome information and some example data to be used in the CandiMeth workflow

It should look something like this:

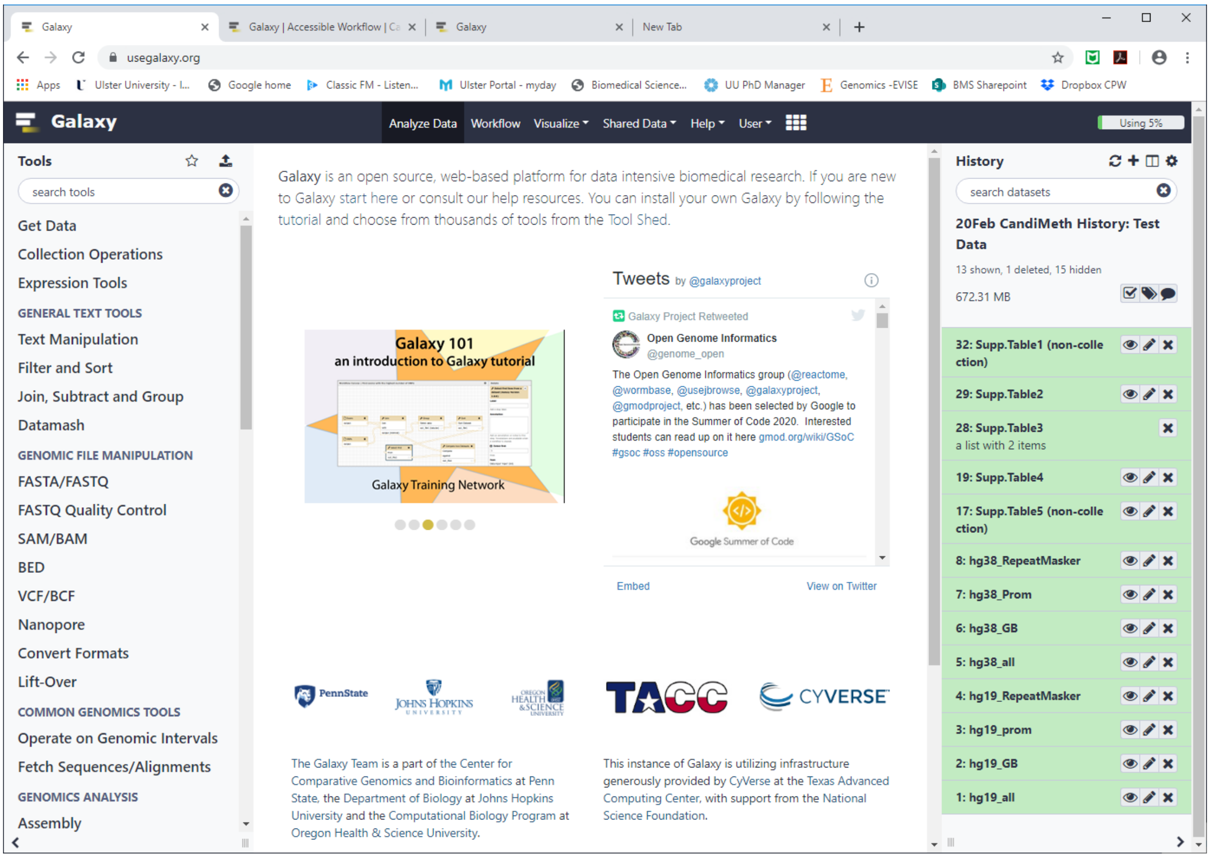

3) The input Differential Methylation Table has to be converted from a table into the form of a Dataset Collection: this is in case there are multiple differential methylation tables to be assessed, then CandiMeth can assess them all simultaneously and present them in the typical Results and

Tracks outputs, as opposed to multiple outputs that might make your history very crowded or initiate multiple histories that may become confusing due to their number.

There are two example files which can be converted and used, one which is an *RnBeads* output (Suppl.Table1) and one a *ChAMP* output (Suppl.Table5).

a) Click on the already checked box at the top of the History panel (mouse over shows "Operations on multiple datasets"): this will cause checkboxes to appear beside all of your datasets as well as some choices to appear at top
b) Check the box beside the Differential Methylation Table dataset e.g. Suppl.Table1
c) Under the pulldown menu beside "For all selected" choose "Build Dataset List"
d) In the window that appears, you can give the collection a new name e.g. "All Probes Set1" and click "Create"
e) A new entry will appear in the RHS with the new name and "a list with 1 (or more) items"- this is the Dataset Collection and is now ready to be processed by CandiMeth
f) -Click on the Check box at top right again as in (a) to go back to normal list view in RHS window

[Note: In the examples here and below, names which the user can choose themself are in green]

It should look something like this at RHS, with the new Dataset Collection ("All Probes Set1") at top

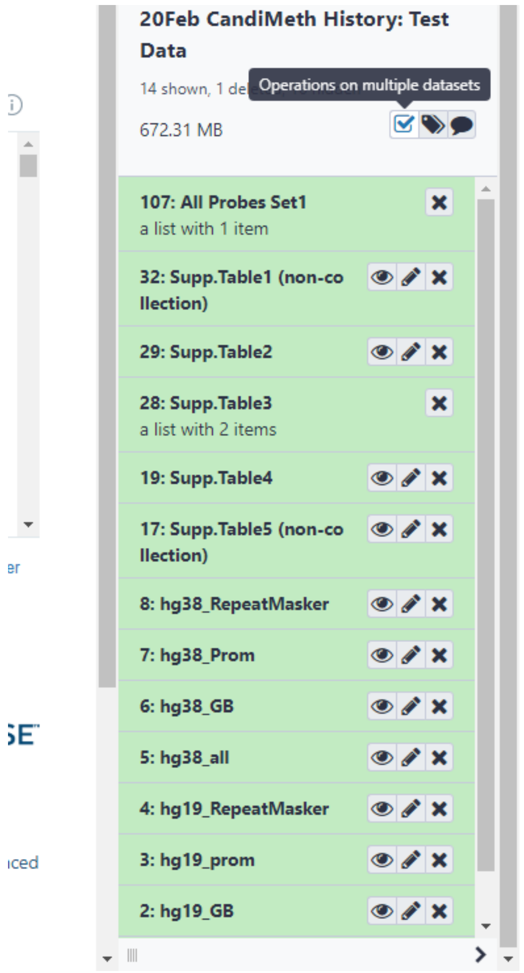

4) Following this, click https://usegalaxy.org/u/sarajayne-thursby/w/candimeth-8 and then click the 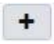 button at the top RHS of the page – this will import the CandiMeth workflow [CandiMeth] to your Galaxy account so you can use it. Click on “Start using this workflow” in the window that appears to bring you to the Galaxy webpage again

This should look something like this:

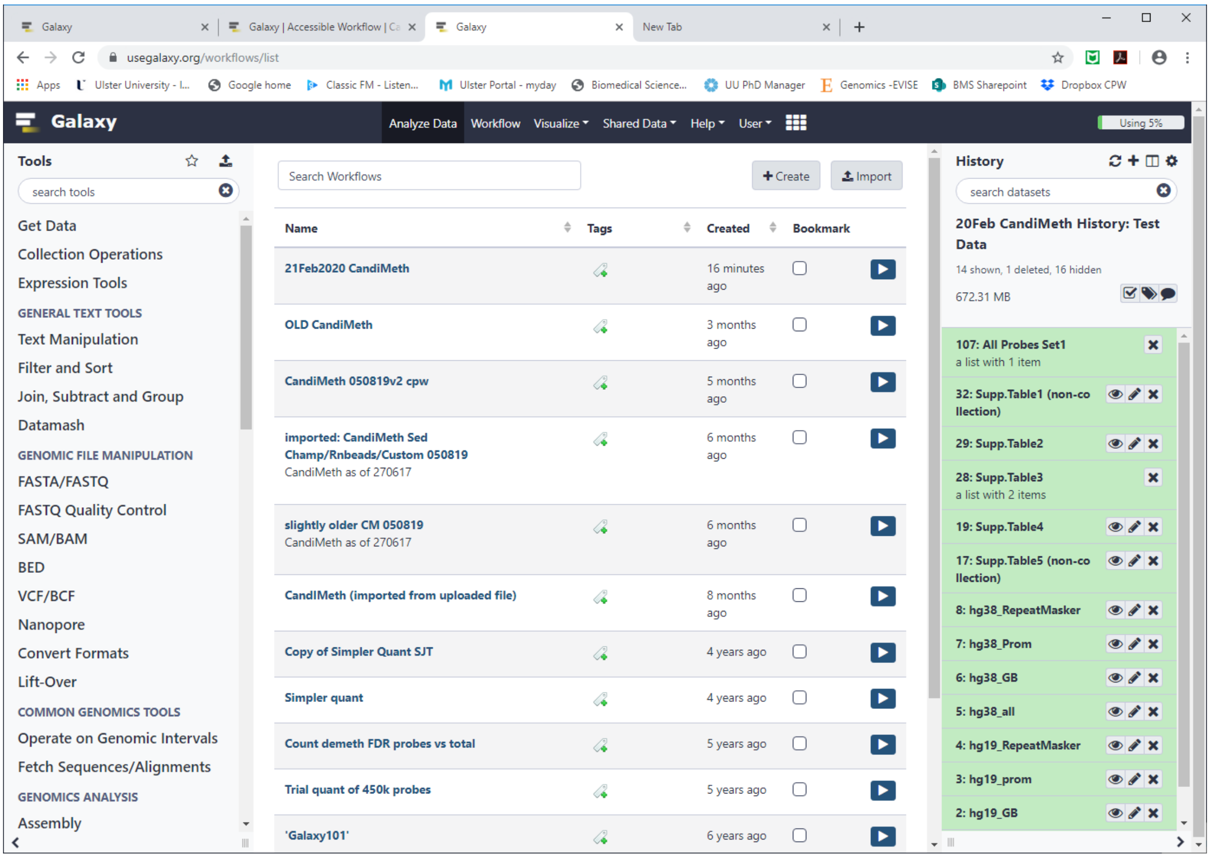

-the central window shows all workflows available to you: CandiMeth should be at the top if it was the last one you imported (the example above shows others the author was using too, will be absent). The RHS window should still be your Test data history.

If you navigate away from this view for whatever reason, you can find it again by going to the top of the Galaxy homepage and clicking on the "workflows" option

You should now be ready for your first test run with the sample data provided.

3. Using CandiMeth with the sample data provided

3.1 Tutorial with the example data provided

The first example takes data from an experiment where two cell lines were compared: one was normal or wild type (WT), the other had lower levels of the DNMT1 methyltransferase enzyme which methylates DNA (d8). DNA from the two types of cell was isolated and run on the 450K Illumina array to determine methylation levels. This data was processed using the RnBeads pipeline, which logged methylation at each position in each cell line, as well as comparing the levels at each position to see if they were significantly different between the cell lines. The RnBeads analysis was output as a table (Supp.Table1). Here we want to see if there are any differences in methylation at certain microRNA genes between the two cell lines: the list of genes is given in Supp.Table2. To do this we choose a version of the human genome map to work with (the version called hg19) and ask to look at all probes associated with the microRNA (hg19_all).

3.1.1 Inputs and starting the run

To start, click on the CandiMeth workflow in your Galaxy page (see step 2.4 above) and on the pull-down menu at RHS marked choose > Run

In the History Options at the top of CandiMeth, "Send results to a new history" click "Yes" (light grey) and give the new history a name of your choosing e.g. "[Date/run identifier] CandiMeth My Test Data"

[NOTE: we advise you to give each new history a unique identifier to avoid confusion]

1. Under <u>1: R Package Used: (1.1)</u> enter 'RnBeads'

2. For <u>2: Input Differential Methylation Table (1.2)</u> choose "All Probes Set1"

[Note: This was the example name used in step 2.3 above, alter as required]

3. At <u>3: Input Gene Features of Interest (1.3)</u> choose "Supp.Table2"

4. For <u>4: Input Genome Release Information (1.4)</u> choose "hg19_all"

You can now click the blue 'Run workflow' button at top right

The Workflow start window with all the above options chosen should look like the screenshot on the next page:

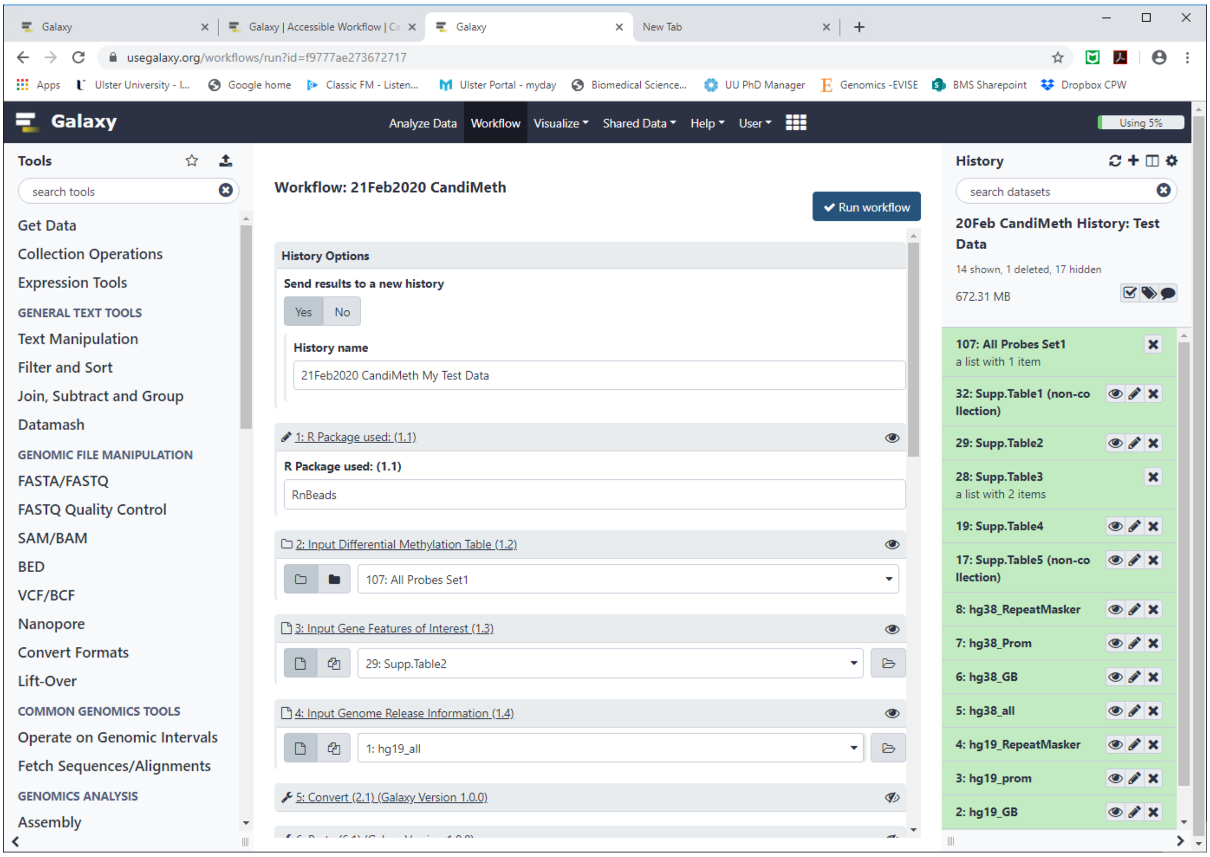

If all goes well, you should see a large green tick in the main (middle) window of Galaxy and the following text:

"Successfully invoked workflow CandiMeth

This workflow will generate results in a new history. Switch to that history now."

The window should also show two status bars, "Step scheduling" and "Job Execution" which will update you on the progress of the jobs.

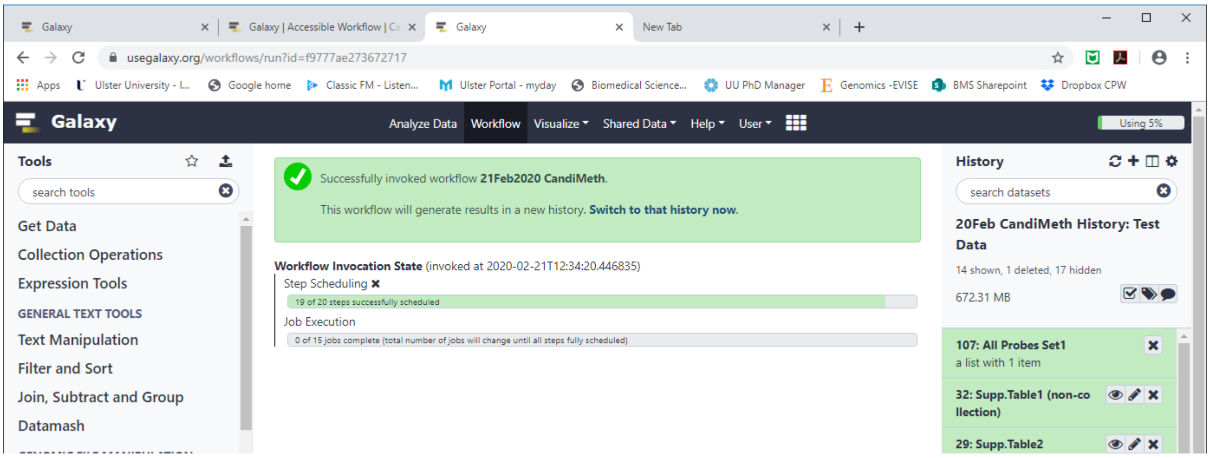

A typical test run with the data above may take ~15 mins to complete. Once both bars have gone green the new ◫ history “[Date/run identifier] CandiMeth My Test Data” is ready.

You can navigate to the new history at any point by following the link “Switch to that history now” at any point, or by navigating between histories using the “Switch to” function at top of the History pane on the RHS.

3.1.2 Overview of outputs from the microRNA analysis

CandiMeth produces two types of outputs; tabular Results and genome browser Tracks.

The outputs will look something like this for the example data above :

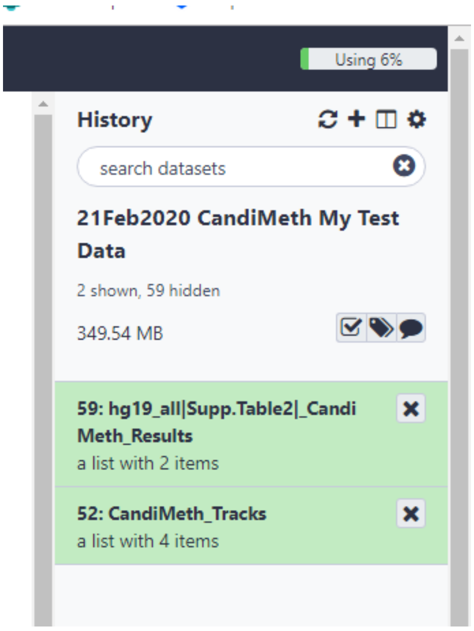

The two green boxes represent the Results and Tracks output collections respectively. The general format of these is as follows, with details changing one ach run. The blue text in each is a link to a more detailed list-

Results:
Number: genome release_probeset|input gene list|_CandiMeth_Results
a list with x items

Tracks:
Number:CandiMeth_Tracks
A list with x items

For the Results, the genome release_probeset and gene list are variables which were decided at the start of the run (see 3.1.1 above), so the choices are recorded in the outputs for clarity. Probeset refers to whether methylation across the promoter only, the gene body only, or both (all) is to be analysed. We will now look at the Results tables (3.1.3) and Tracks (3.1.6).

3.1.3 Working with the output Results tables

1. To access the tabular results, click on the link saying *CandiMeth_Results* which will open a new window at RHS. For the example data here, this will show the two items in the list (see screenshot at left below). One is a table of methylation values across microRNA genes in the WT cells mean_beta_WT_of_D8_Track and the second the methylation values in the cells with the lower DNMT1 enzyme levels mean_beta_D8_Track.

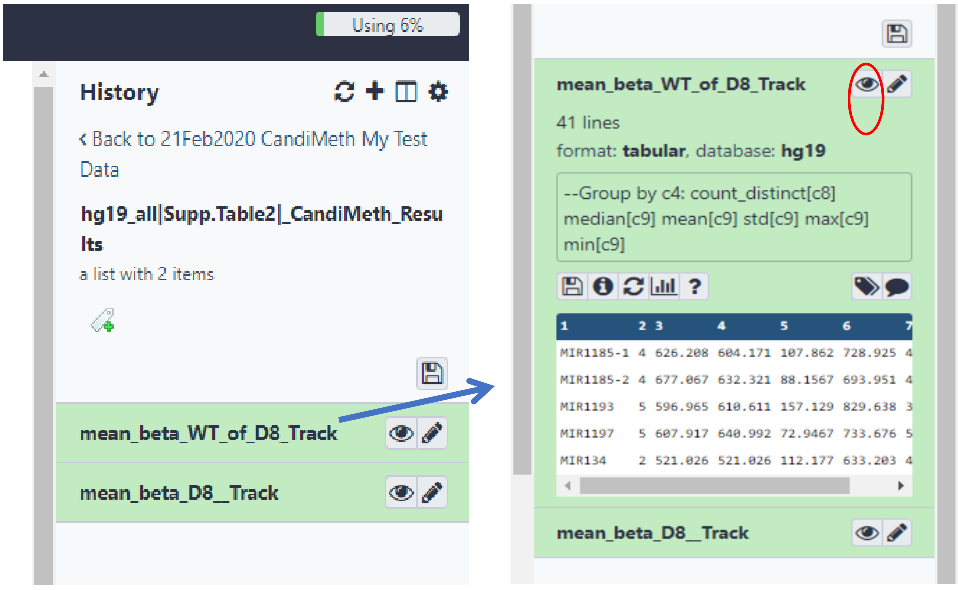

2. Clicking on the mean_beta_WT_of_D8_Track will show a preview (screenshot at right above) of the first 5 lines of the data table, as well as the header and other information.
3. To see a full table, click on the eye symbol (circled above) and the full table of data will appear in the central Galaxy window as shown below

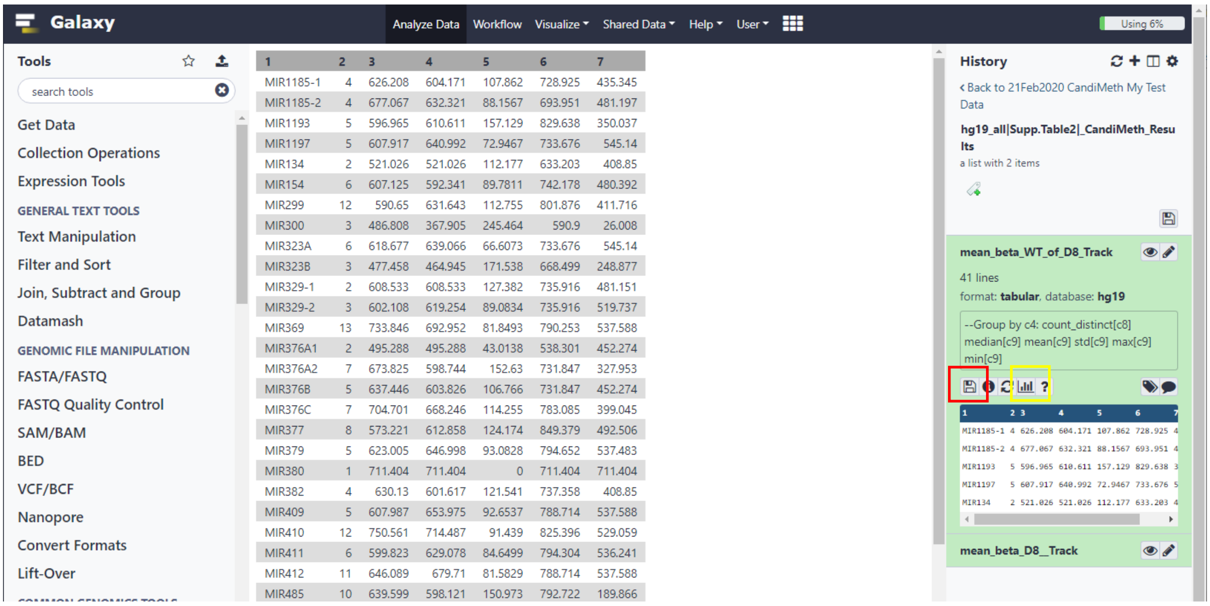

There are no headers in Galaxy but the key to the columns is as follows:-

1) Name of gene, 2) Number of array probes, 3) Median methylation, 4) Standard deviation, 5) Mean methylation, 6) Maximum value and 7) Minimum value

You can work with the results directly in Galaxy, using the Galaxy graphing and stats tool, or you can save (red box in screenshot above) the data from Galaxy onto your computer to allow you to work with it in other graphing and statistics programs such as Excel and SPSS, see section 3.1.5 below for this latter option.

3.1.4 Graphing data directly in Galaxy

This option is available to the user for each table: click on the button in the RHS window (boxed in yellow above). As an example we will use the *Bar diagram (NVD3)* option in the central Galaxy window.

Below we reproduce the first part of the table for reference:-

1	2	3	4	5	6	7
MIR1185-1	4	626.208	604.171	107.862	728.925	435.345
MIR1185-2	4	677.067	632.321	88.1567	693.951	481.197
MIR1193	5	596.965	610.611	157.129	829.638	350.037
MIR1197	5	607.917	640.992	72.9467	733.676	545.14
MIR134	2	521.026	521.026	112.177	633.203	408.85
MIR154	6	607.125	592.341	89.7811	742.178	480.392
MIR299	12	590.65	631.643	112.755	801.876	411.716
MIR300	3	486.808	367.905	245.464	590.9	26.008

The key to the column headers is as above (3.1.3 step 3): Name, number of probes, median etc On the Bar diagram tool window in the centre window we generated the following chart showing median methylation at the microRNA by choosing column 3 (median) in the Data series window, and adding labels etc to the graph. This is one example, there are many other options to explore.

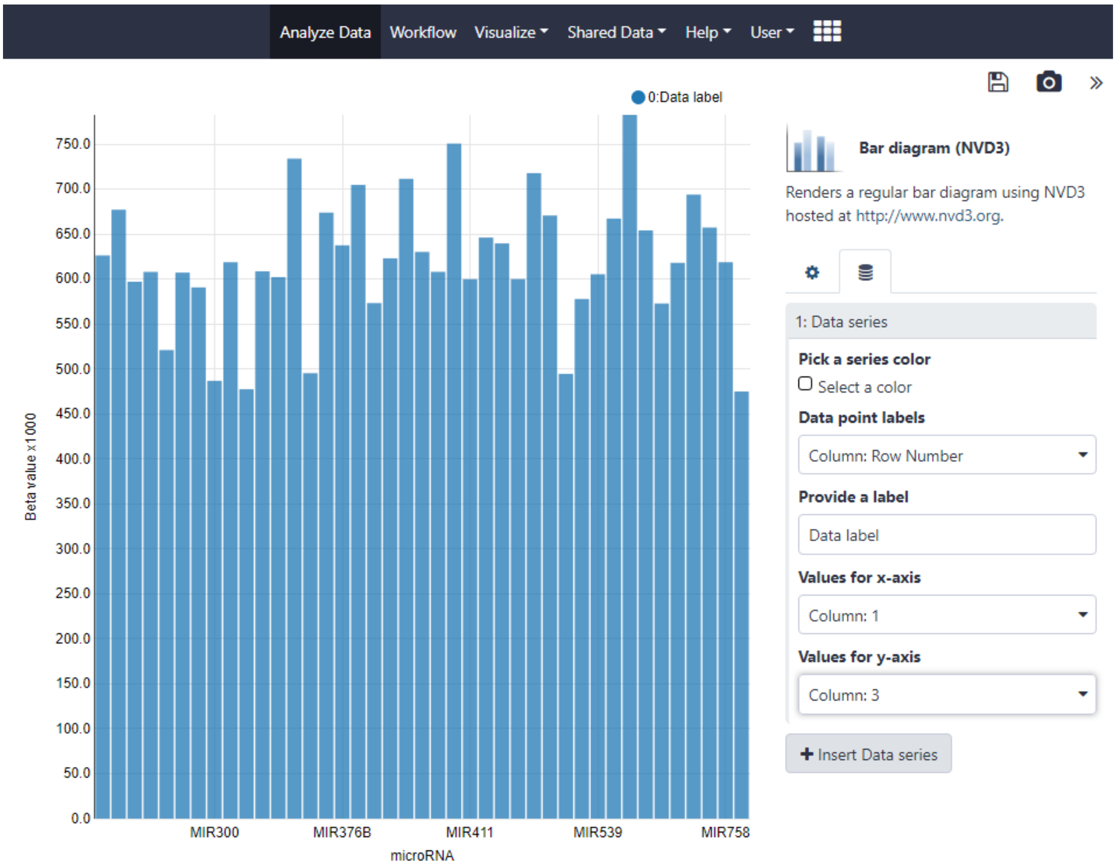

3.1.5 Exporting data from Galaxy to Excel

Tables of generated results can be downloaded and imported into programs such as Excel and Notebook by selecting the button just above the individual output in the RHS window (see p9): this will download the data in a generic tabular file format. The name of the file will be similar to that seen before (see 3.1.2 above), but will have some additional information, an example is-

GalaxyNumber: genome release_probeset|input gene list|_CandiMeth_Results_mean_beta.tabular

This file will have a .tabular suffix which allows it to be imported into a number of different programs such as SPSS or Excel. You should first save or move it to a specific folder on your computer.

To import a .tabular file into Excel:

1. Open Excel and using the Open command, locate the folder containing the .tabular file [Note: the file may not be visible unless you choose “all files” in the pull-down menu to the right of the *File name* window, since it is not a standard Excel suffix (.xls)]

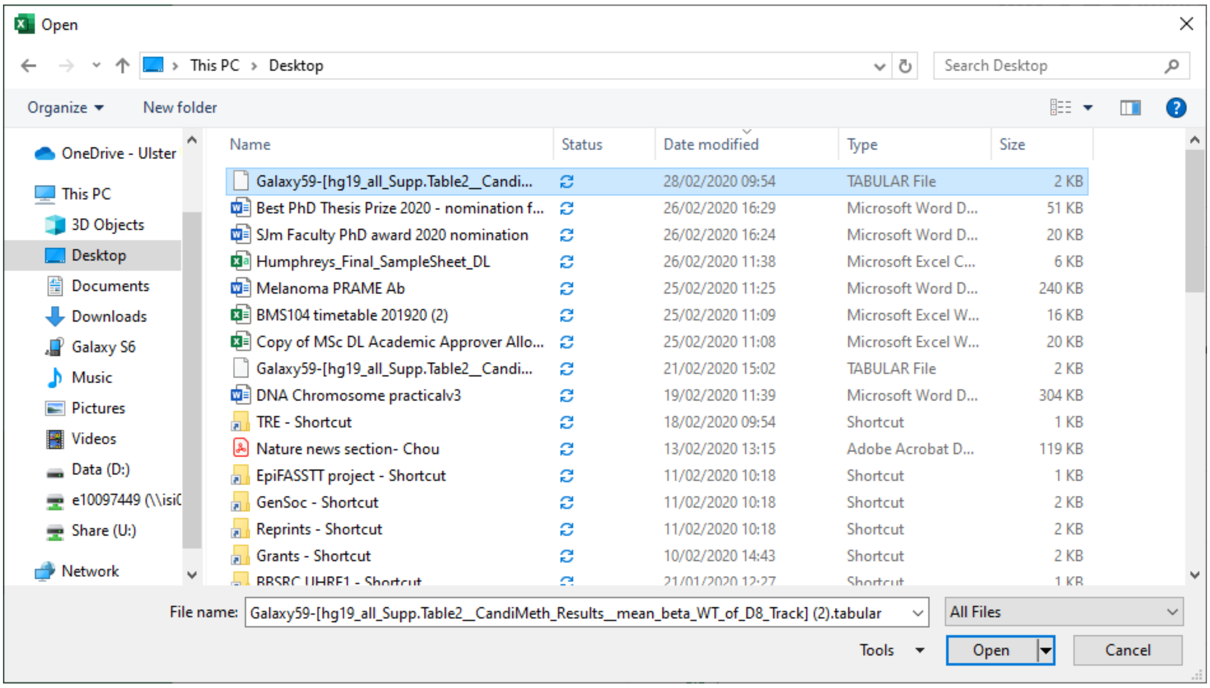

2. Select the Galaxy file you want to import into Excel as above, then click Open. This will cause the Text Import Wizard window to automatically open

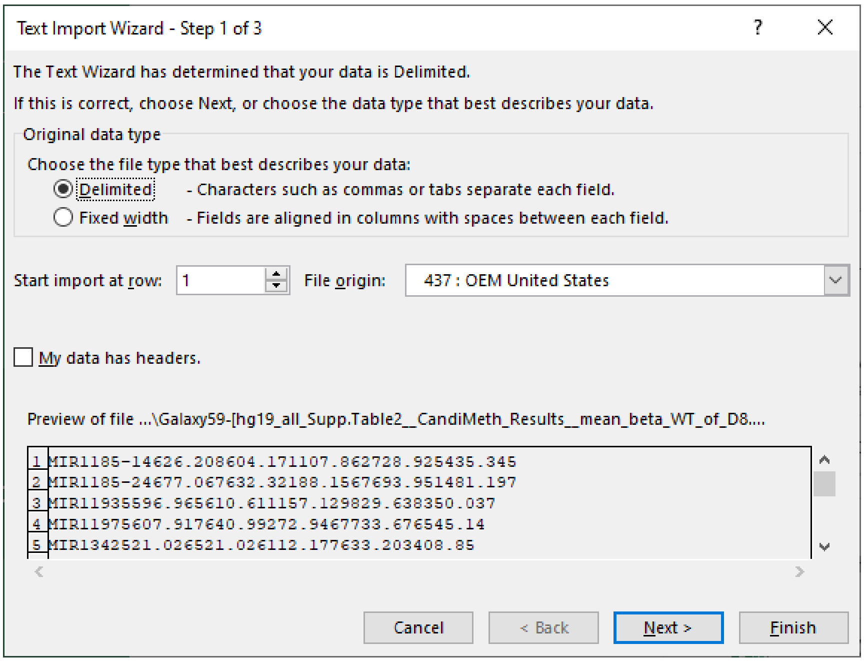

3. Go with the default option *Delimited*, click next and on the next window the default *Tab*,

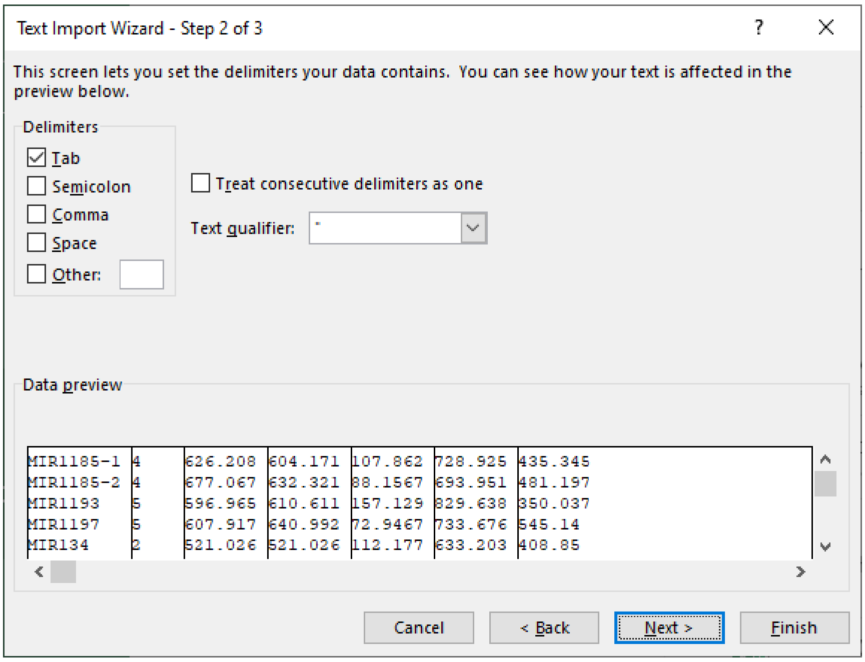

4. In the third and final window choose *General* (for formatting of columns): the imported file should then automatically open in Excel and look like the window below-

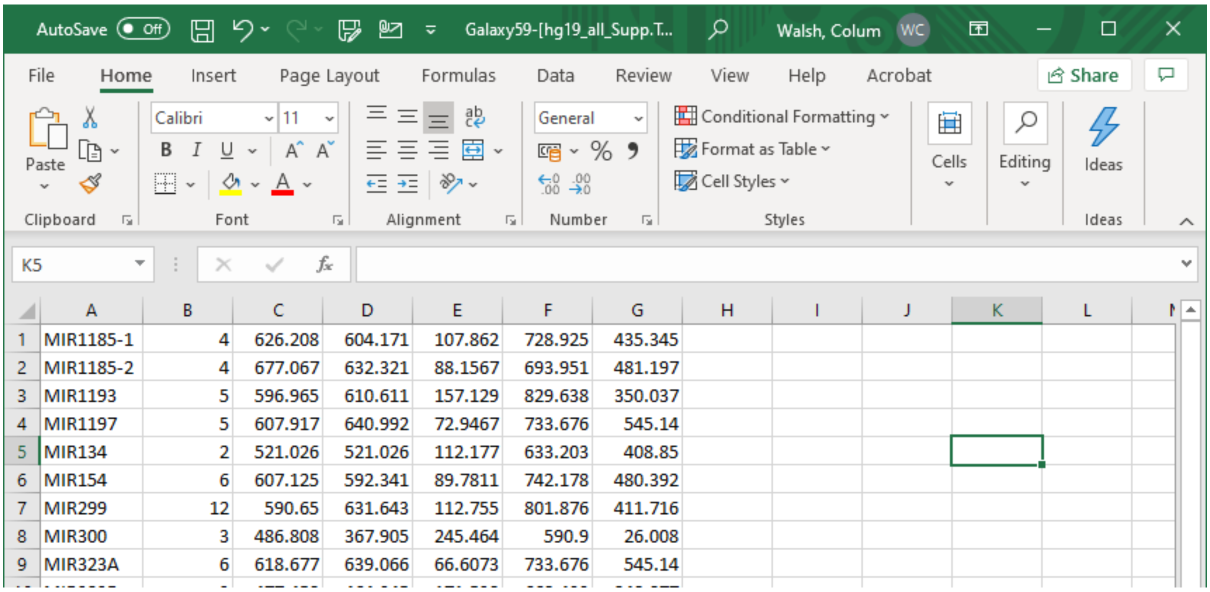

	A	B	C	D	E	F	G
1	MIR1185-1	4	626.208	604.171	107.862	728.925	435.345
2	MIR1185-2	4	677.067	632.321	88.1567	693.951	481.197
3	MIR1193	5	596.965	610.611	157.129	829.638	350.037
4	MIR1197	5	607.917	640.992	72.9467	733.676	545.14
5	MIR134	2	521.026	521.026	112.177	633.203	408.85
6	MIR154	6	607.125	592.341	89.7811	742.178	480.392
7	MIR299	12	590.65	631.643	112.755	801.876	411.716
8	MIR300	3	486.808	367.905	245.464	590.9	26.008
9	MIR323A	6	618.677	639.066	66.6073	733.676	545.14

5. This should exactly match your data output from Galaxy. There are no headers in Galaxy so you should add the row of output labels at top yourself:-
   column A) Name of gene, B) Number of array probes, C) Median methylation, D) Standard deviation, E) Mean methylation, F) Maximum value and G) Minimum value.
6. It would be sensible to give the Excel table a simpler name reflecting the data type eg "MicroRNA methylation in WT cells", but each file will have the unique identifiers automatically embedded in the long file name by default
7. In a similar fashion the data on methylation in the D8 cells can also be imported into an Excel file. Data from these files can then be cut and pasted into one file to allow direct graphing and statistical comparisons in Excel as indicated in the CandiMeth paper and bibliography therein

To navigate back to the window showing both Tracks and Results, just click on the link at the top of the RHS screen which should say "*<Back to….My Test Data*" or similar

3.1.6 Working with output Tracks

To access the Tracks generated as output, from the results History generated from your run, click on the link saying "*CandiMeth_Tracks* (a list with x items)", which will open a new window at RHS. For the example data here, this will show the four items in the list below (see screenshot at left). These are the two tracks showing absolute methylation (beta value) in 1)the WT cells used as a control mean_beta_WT_of_D8_Track and 2)the D8 cells which have decreased DNMT1 levels mean_beta_D8_Track. There are also two tracks showing comparisons between the WT and D8: these are 3)the track showing difference in methylation (delta beta or $\Delta\beta$) between WT and D8 called delta_beta_D8vsWT_Track and 4)a track showing only those probes where the difference in methylation is significant at a false discovery rate of 0.05, called FDR_D8_of_WT_Track.

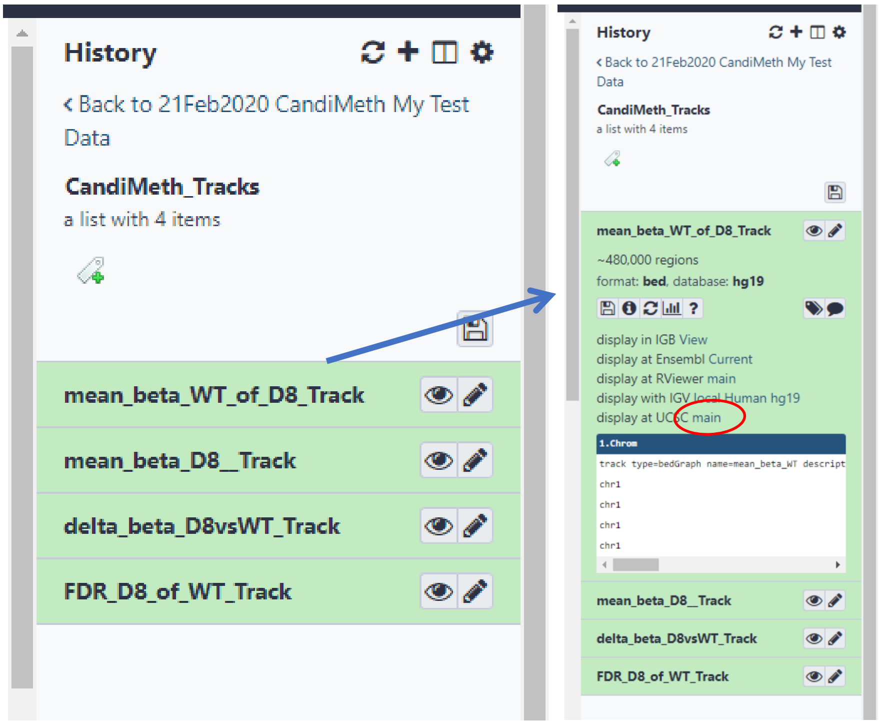

Clicking on the name of the Track at left e.g. mean_beta_WT_of_D8_Track will show the preview window (see RHS above). This should say under the title ~480,000 regions" for the 450K array and "format:bed, database:hg19" indicating that a type of track called a BED file has been generated, using the hg19 edition of the human genome map. The first five lines of the track data will also be shown, but this is a long table with 480,000 rows! To visualise the data, we instead:-

1. Click on one of the "display" options in the preview window: CandiMeth is optimised for use with the UCSC browser, so click on "display at UCSC main" by following the hyperlink in blue.
2. There will be a small delay, then a new tab will open in the browser, taking you to the familiar UCSC genome browser page, with the data from your first track displayed at the top (see next page for screenshot).
3. The default tracks on UCSC include roughly one from every major group (blue header), and at writing were UCSC_Genes, NCBI_RefSeq, Publications, GTEx gene, ENCODE regulation, Conservation and dbSNP_153. Your track will appear under the Custom Tracks header at top as mean_beta_WT. As this makes the window quite complex and busy, you

may wish to simplify the view by clicking on the “hide all” button (boxed in red on screenshot below), then add back just the locations of genes using NCBI_RefSeq >pack and mean_beta_WT>full (both boxed in yellow below). This should give you a simpler view which is easier to work with, similar to that below when using the test data:

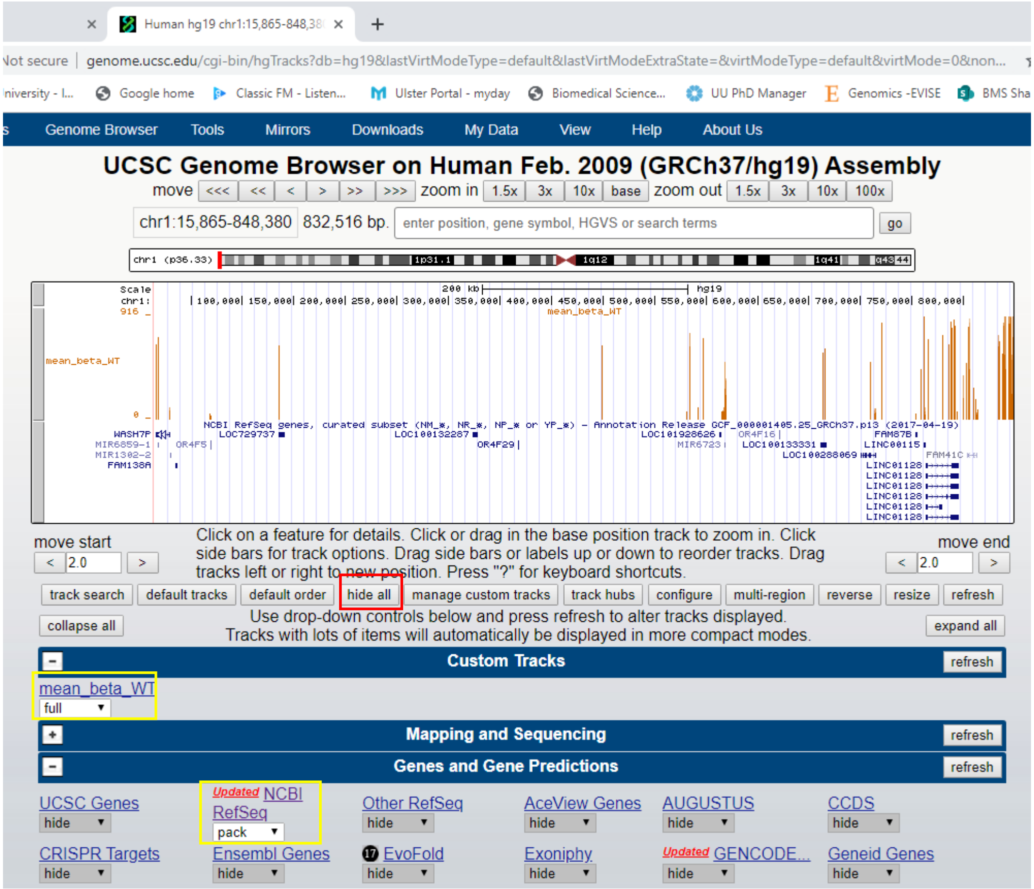

4. You now have a display showing the start of the genome map (chromosome 1p) with genes displayed in blue on the NCBI_RefSeq track at bottom, with methylation levels in the WT cells shown as peaks in the mean_beta_WT track along the top in brown.

   The height of each peak in the mean_beta_WT track corresponds to the methylation level at that position, with the minimum and maximum values seen in this window displayed at left as beta values x 1000 (0 and 916, equivalent to 0% and 91.6% methylated respectively).

5. This is a fully zoomable map as usual for UCSC: to illustrate, if you draw a box around the gene just visible at right above, *LINC01128,* this will magnify the view of that gene, showing the locations and extent of methylation at each probe across the gene (below)

   [Note: if a window opens for “Drag-and-select” simply tick “don’t show this again” and “zoom in”]

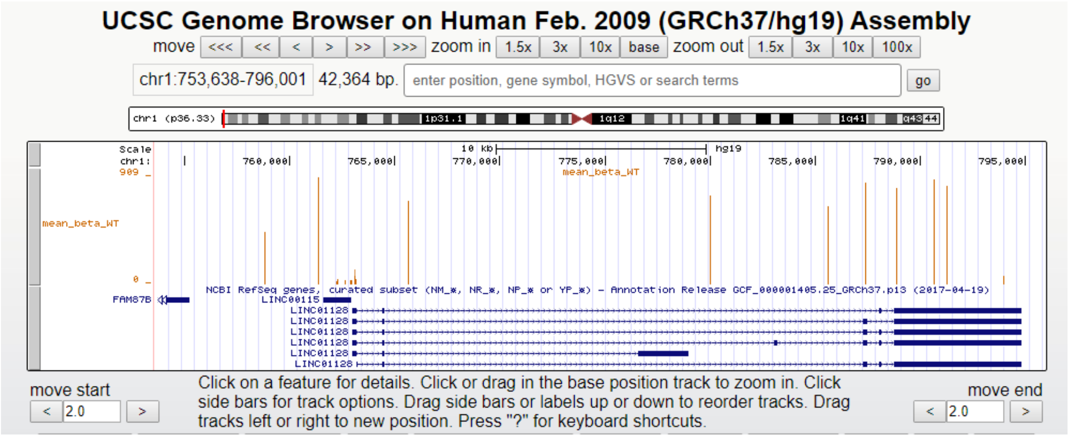

6. To overlay any other data on this map, simply choose from the UCSC pull-down menus: e.g. to show the locations of common SNPS, choosing dbSNP_153>dense under the *Variation* header further down the UCSC main controls will overlay a track with this information underneath the other tracks (arrow below)

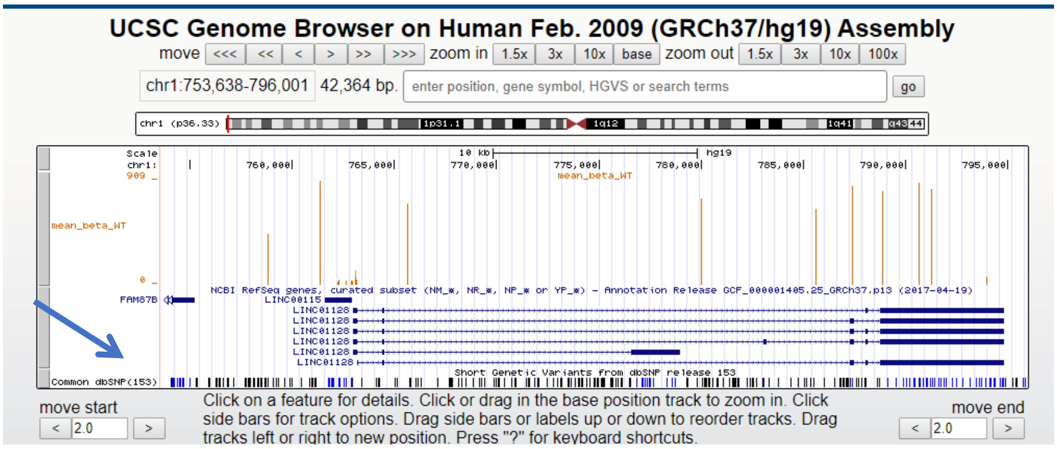

7. The screen currently only shows the data from one of your four tracks generated using the example data: to bring up the next track, follow steps 1-2 above for mean_beta_D8_Track: this will open a new tab in your browser showing the new data AND the track you already generated
8. Do the same (steps 1-2) for the remaining two tracks delta_beta_D8vsWT_Track and FDR_D8_of_WT_Track; the last window you open will now show ALL FOUR tracks (see screenshot below) and other tabs can be closed

[Note: leave the Galaxy tab open in the background, to allow access to Results etc]

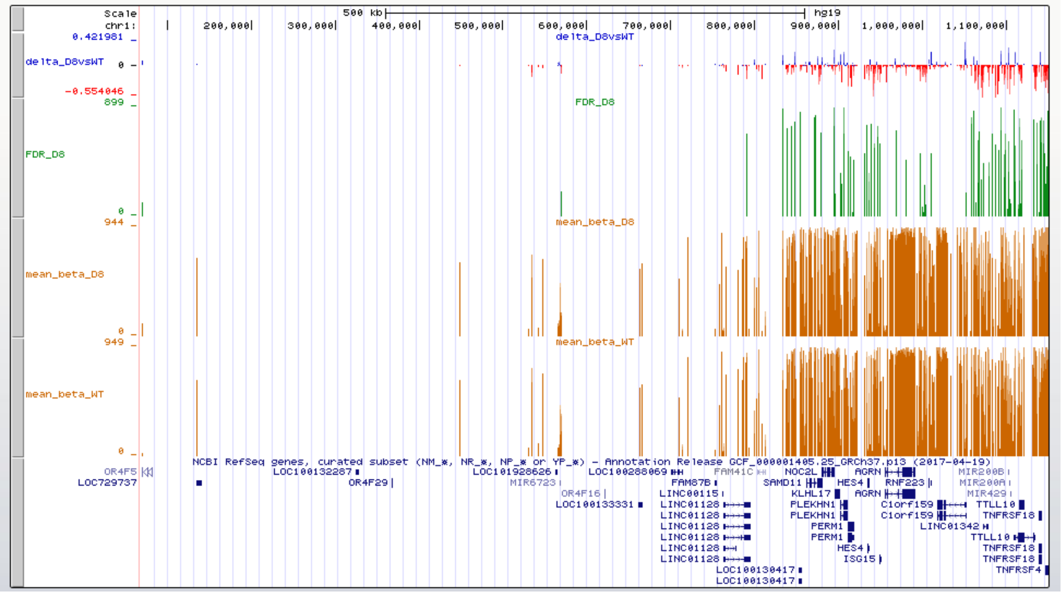

9. The window containing all the track data should look much as in the screenshot above for the example data: this shows the chromosome position and coordinates at the top, then the four tracks:
    a. delta beta track showing gains (blue) or losses (red) in D8 cells vs WT cells,
    b. the FDR track showing which of those differences is significant (green)
    c. the absolute methylation levels in the D8 cells (brown)
    d. the absolute methylation levels in the WT cells (brown)

10. Zooming in on LINC01128 as before (step 6 above) should produce a map like:-

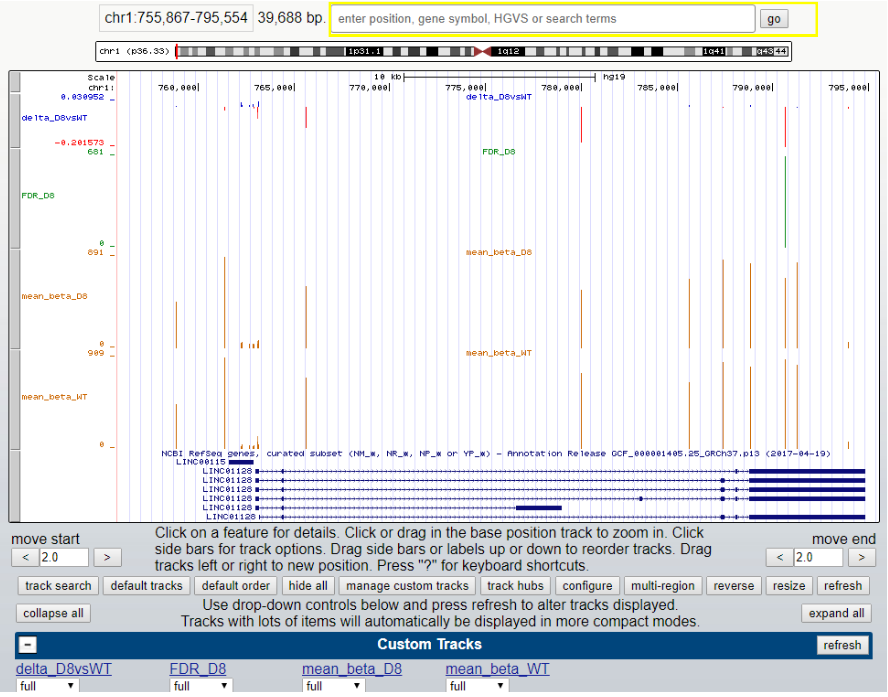

11. This shows that while we can see methylation levels are high across the gene, they only drop at a few positions (red at top), with only one (green) being FDR-significant.

12. Note that individual tracks can be toggled on or off with the buttons under Custom tracks, then hitting “refresh”: this can be particularly useful to just look at differences in methylation (delta beta)
13. While the Tracks open by default at the start of the genome map (chr1p), you can look at any gene in the human genome by typing its name into the search box at top of the screen (yellow box at top of last screenshot)
14. For this example, type “MIR1185-1” into the box: as you type, the name should appear under the box- click on the name to take you straight there

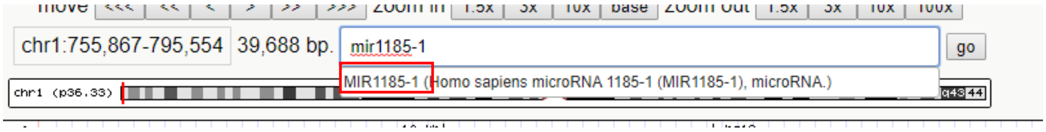

[Note: this is a short-cut to the location of this MIR gene as decided by UCSC]

15. Alternatively, if you type MIR1185-1 and hit “Go”, the next screen will give you a set of alternatives, based on who has mapped the gene

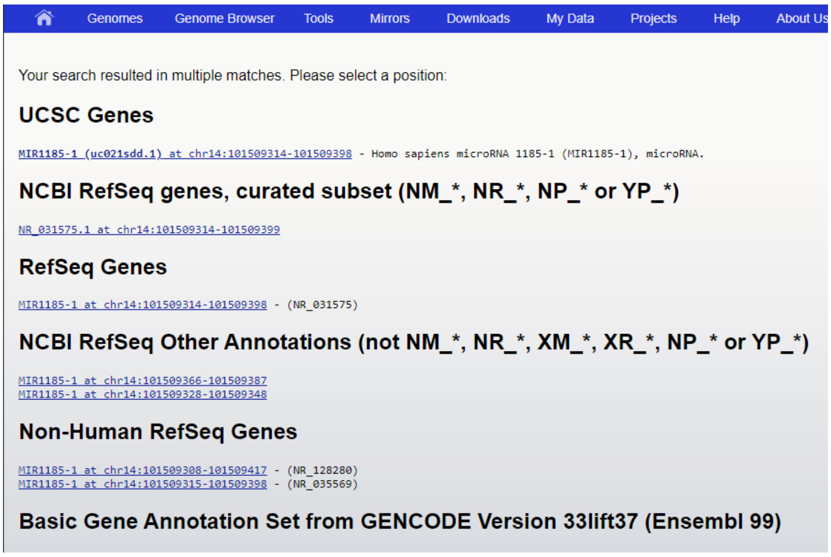

- you can click on any of the options to bring you to the map location indicated: hitting UCSC will bring you to the same location as in step 15 above. This screen can be useful when there is some dispute over map location of genomic features.

16. Following either steps 15 or 16 above will bring you to a zoomed-in map of MIR1185-1: this shows ONLY the body of this small gene, so information from only one array probe is visible at right at large thick bars

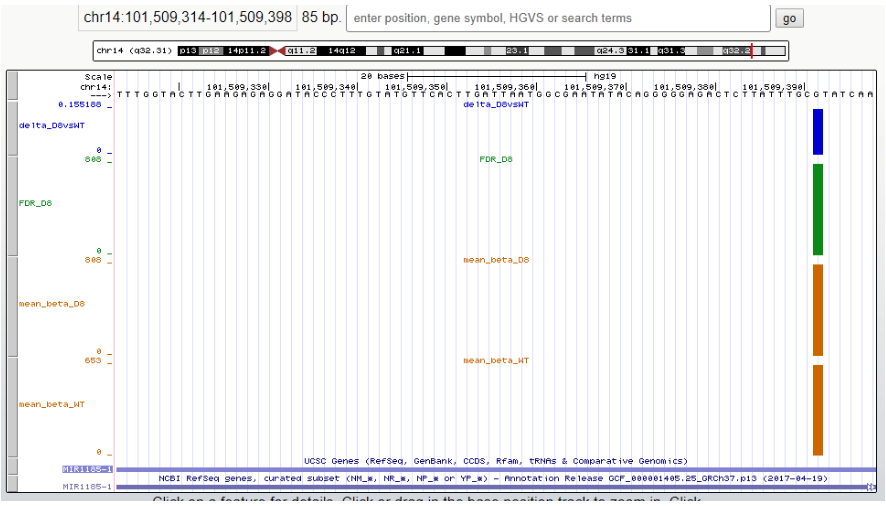

17. To get a better view of the promoter and surroundings, use the 10X zoom-out buttons

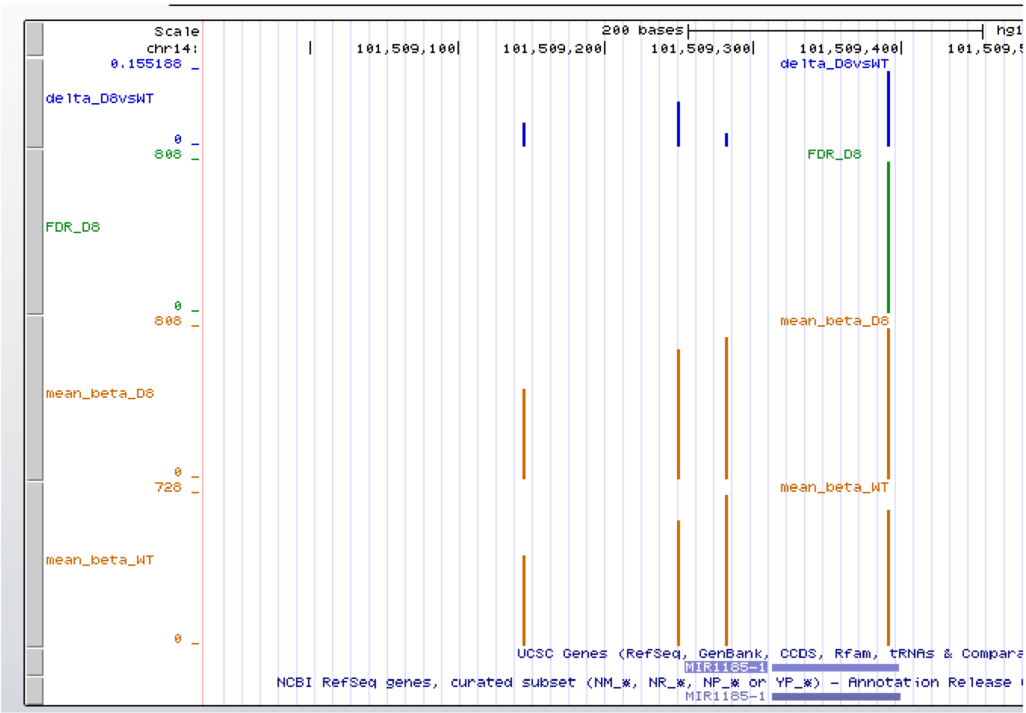

18. This shows a view of the four probes associated with this MIR: the mean and median methylation of these four probes were captured in the Results tables under 3.1.3 above
19. From the Tracks here it can be seen that the methylation is much lower at the probe furthest away from the gene (to left above), while the only probe showing significant differences in methylation between WT cells and those with low levels of DNMT1 (D8) is the probe at right (green above)

3.1.7 Exporting browser views as graphics files

It is often the case that the user wants to show a particular UCSC genome browser view of the data. You may also wish to modify the view slightly by, for example, removing the grid lines (a common request from journals). These facilities are provided by UCSC and can be accessed as follows:-

1. On the blue top ribbon in the UCSC browser window, click on View button, which will bring up a number of options in a pull-down menu

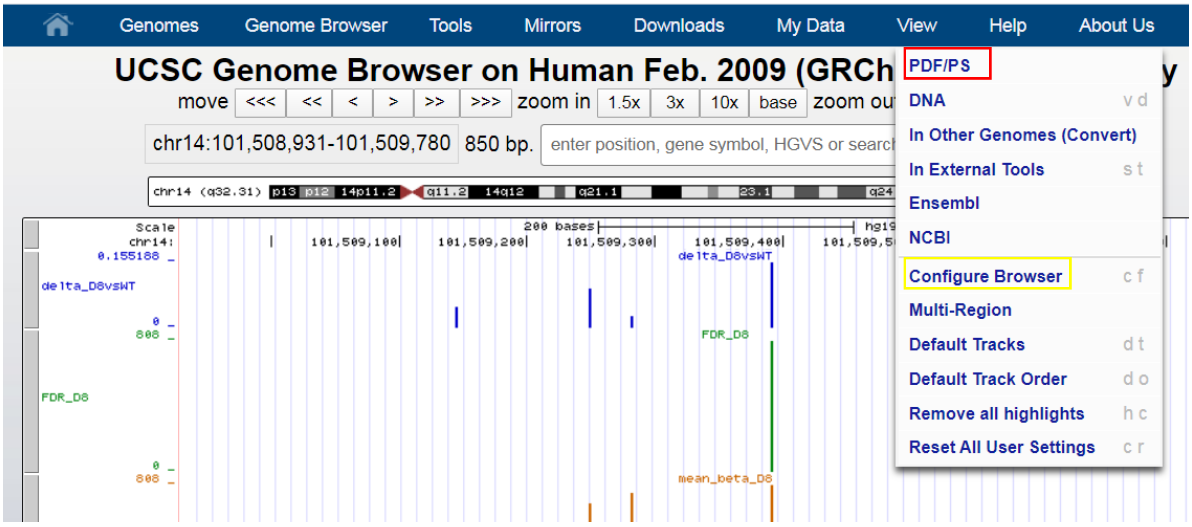

2. To first tidy the image, you can click on >Configure Browser (yellow box above)
3. Uncheck the boxes for “Show light blue vertical guidelines” and “Display description above each track” and click the gray “submit” box at top left
4. Your browser will return to the image you were viewing, which should now have no gridlines or labels in the middle of the screen
5. To export this view in a format you can include in documents, or further adjust in another program, click on the View>PDF/PS option (red box in top screenshot)

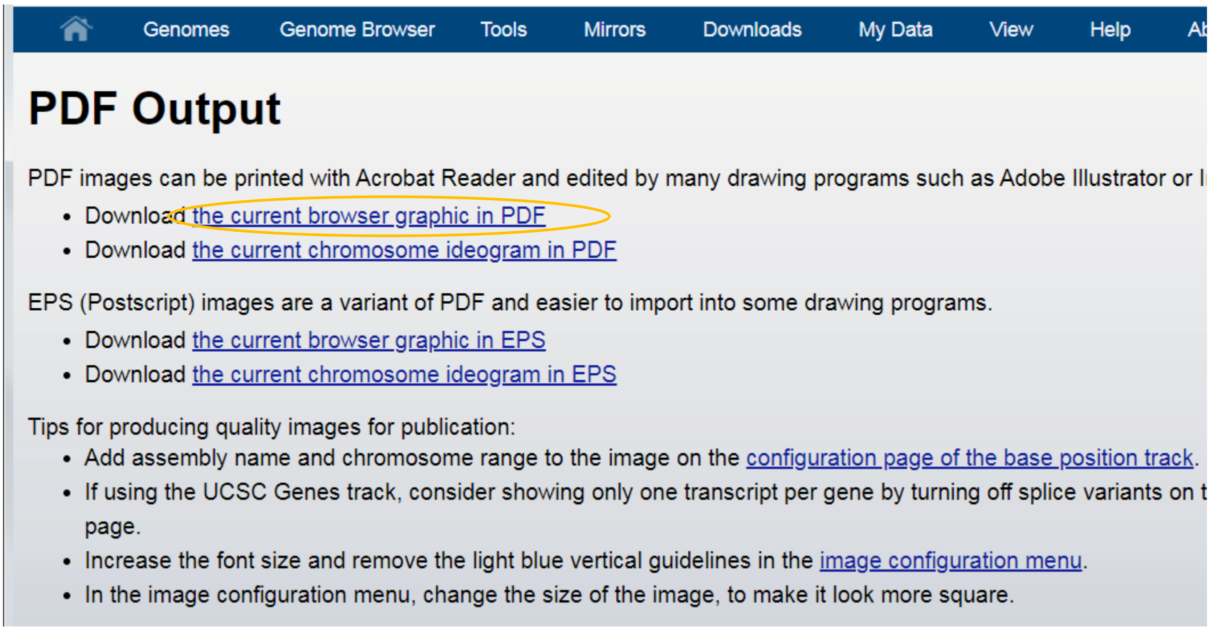

6. A new screen will appear (below) with a number of options: to save as a PDF file click on the top option (orange box above) [Note the tips for publication-quality images here]

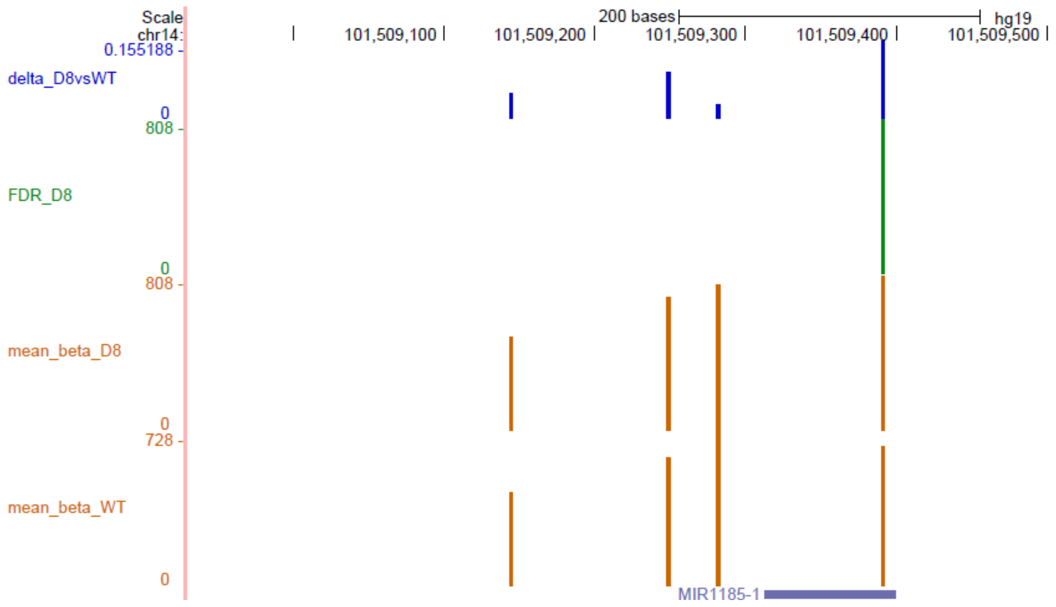

7. This should open a new screen showing the PDF version of the genome browser view (above), which can be downloaded and inserted in documents
8. As well as PDFs, postscript (PS) file format is also supported: most graphics software programs can import files in one or the other format for further adjustment if needed e.g. Adobe Illustrator or Photoshop

3.1.8 Quantifying methylation in different parts of the gene

In the example above, we looked at methylation across the whole gene locus (promoter and gene body) for the microRNA genes in our list. CandiMeth is however designed to look at different parts of the gene, as these can often behave differently.

3.1.8.1 Promoter methylation only

To ONLY look at the methylation in the promoter regions of the microRNA genes, the same settings as in 3.1.1 above can be used EXCEPT that under Input 4 choose “hg19_prom”

[Note: if you have already run CandiMeth, remember to switch back to the original history containing the test data “[Date/run identifier] CandiMeth My Test Data” History by using the “switch history” tool ◫ in the top RHS of the screen: choose the history needed by clicking the grey “*Switch to*” button at top left of the History, then >*Analyse data* on the top bar of Galaxy. You should return to the standard Galaxy view but with the desired History in the RHS window]

The output tables in the Results folder will now only average the methylation across the probes found in the MIR promoters (defined as -500bp to +1bp from the gene start).

3.1.8.2 Gene body methylation only

To ONLY look at the methylation in the gene bodies of the microRNA genes, the same settings as in 3.1.1 above can be used EXCEPT that under Input 4 choose “hg19_GB”

The output tables in the Results folder will now only average the methylation across the probes found in the MIR gene bodies (defined as +1bp from the gene start, through all of the exons and introns, to the transcriptional end site (TES)).

That these two parts of the genes can vary significantly, or even show opposite effects, is well-documented in the literature and can be illustrated by the graphics below:

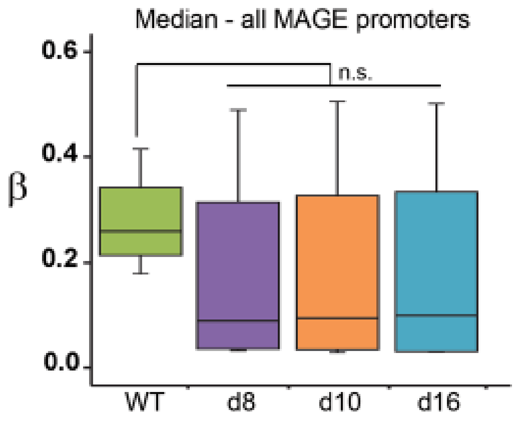

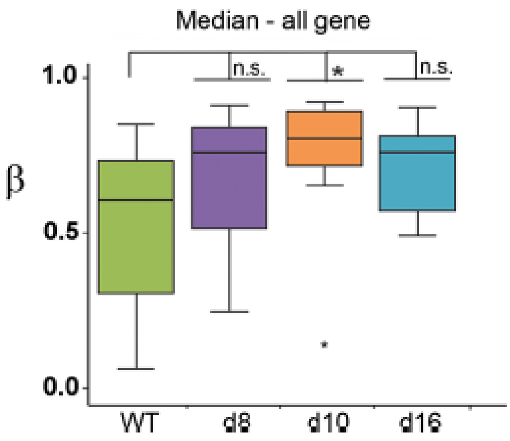

This shows that median methylation (β value) of the promoters of MAGE genes decreased in D8 and other DNMT1- depleted cell lines relative to WT (left), while methylation at the gene bodies went up (right). [graphic generated in SPSS after CandiMeth analysis, see O’Neill et al E&C 2018]

3.2 Looking at a new set of genes in the current methylation array dataset

In the example above, the microRNA (MIR) gene list (Supp.Table 2) was used to query the methylation array data from the comparison of DNMT1-depleted and WT cells (Supp.Table1, converted into a collection All Probes Set 1). Once array data has been uploaded and converted however it is perfectly possible to look at any other gene or genes you are interested in.

1) Navigate back to the History containing the Test data, which includes All Probes Set1 (the data you will query) by using the navigation symbol as before (see note in 3.1.8.1 above)
2) Now on the left hand side (LHS) Tools window choose >*Get data* (yellow oval on screenshot) then >***Upload file*** *from your computer* (red oval)

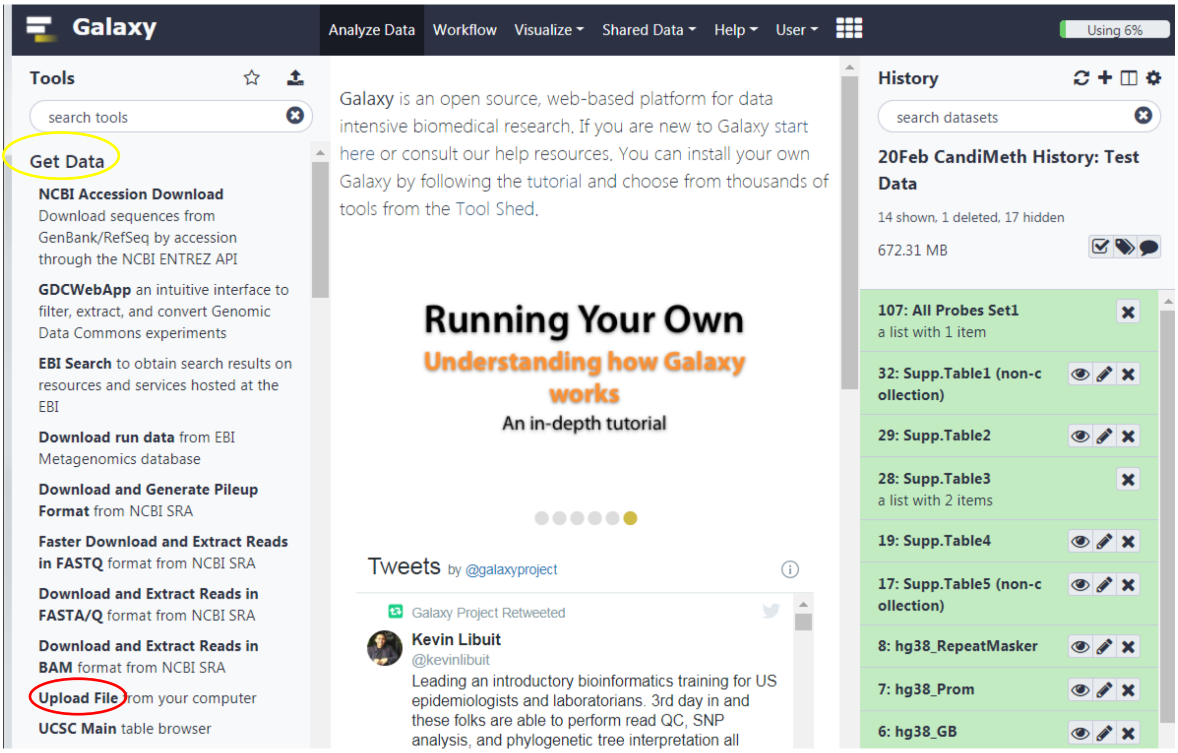

3) This will open a new window where you can put in the names of the genes you are interested in in several different ways (a-c below), choose that which suits best:

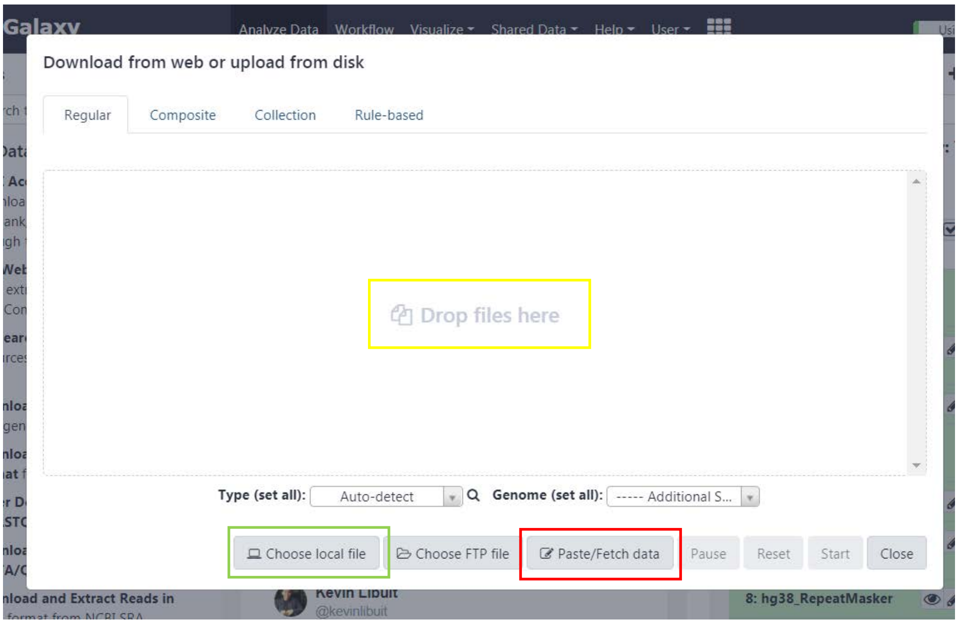

a) Click on >*Paste/Fetch data* (red square in screenshot above), then just type or paste in the names of the genes you wish to investigate onto separate lines as in the example below (here the three genes from Case Study 2, main CandiMeth paper)

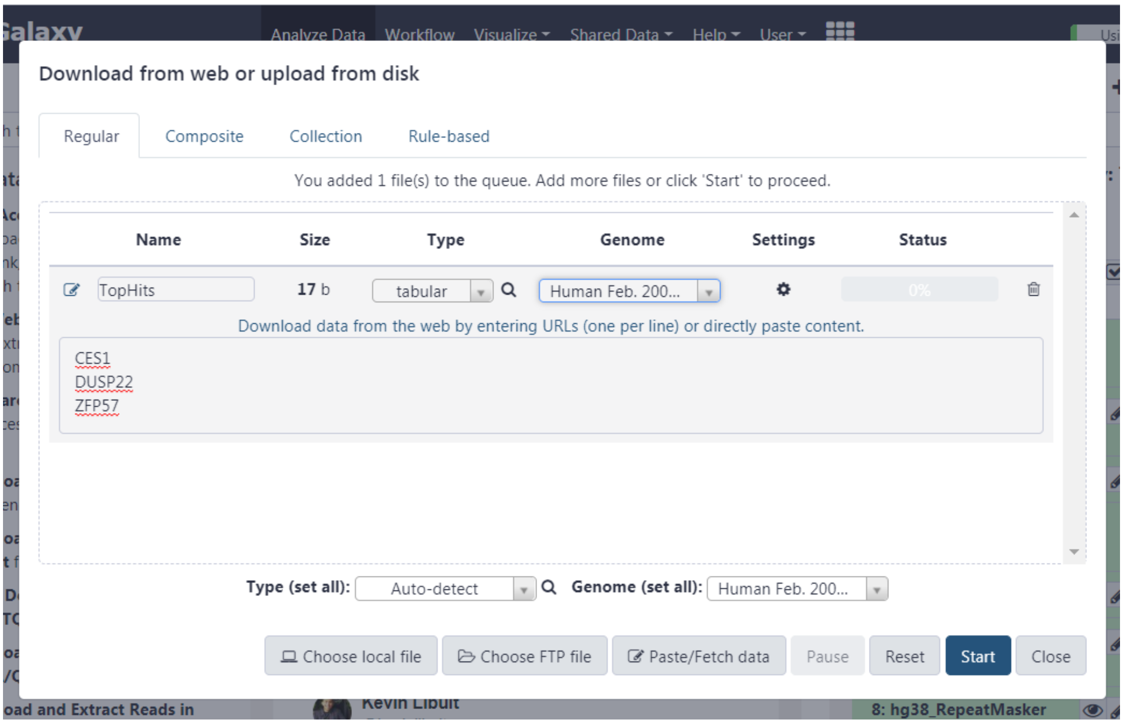

-Along the top of the window, give the new list a **Name** e.g. "TopHits", choose >*tabular* under **Type**, and under **Genome** choose >*Human Feb.2009 (GRCh37/h19) (hg19)* [this will appear as an option if you start to type *hg19*]

-Click "Start": the file should upload to Galaxy and appear as a separate dataset on the RHS with the name you gave it, in this case "TopHits": you can close the Upload window and go to step 4

b) If you have a longer or more complex list, this can be written in a word-processing program such as Word and saved as a *text only* or *Plain text* file (*.txt), before uploading directly using the uploaded directly from a .txt file format (e.g. Supp Table 2) by following steps 1-3 above i.e. >Upload file >Choose local file, the format should be Tabular

c) You can also simply drag and drop a text-only file created as in (b) into the window shown at the start of step 3 above

4) This new list can then be used to query the array data by Running CandiMeth and choosing "TopHits" as Input 3 (See 3.1.1 step 3) instead of the MIR gene list in Supp.Table2

5) The Results folder will now contain Tables showing methylation levels for the new list of genes

3.3 Looking at repetitive DNA elements such as LINES and ERV

Many epigenome-wide studies are interested in assessing methylation at repetitive DNA elements instead of endogenous genes, often using a wet-lab analysis technique such as pyrosequencing to assay LINE-1 methylation for example. A substantial number of probes on the 450K and EPIC arrays fall within repetitive DNA elements however, allowing analysis of methylation across these elements. As an example, we can look at methylation across repetitive elements in the WT and d8 dataset. To do so we:

1. Switch to the original history containing the test data "[Date/run identifier] CandiMeth My Test Data" using the "switch history" tool in the top RHS of the screen [see note in 3.1.8.1 above]

2. Under >Workflow on the top black Galaxy header, click on the CandiMeth workflow and on the pull-down menu at RHS marked choose > Run

3. Choose to Send the results to a new history e.g. "[Date/run identifier] CandiMeth Repeats"

4. Under <u>1: R Package Used: (1.1)</u> enter 'RnBeads'

5. For <u>2: Input Differential Methylation Table (1.2)</u> choose "All Probes Set1"

6. At <u>3: Input Gene Features of Interest (1.3)</u> choose "Supp.Table4"

   [Note: this contains the names of the different types of repetitive DNA as identified by the *RepeatMasker* program (see below)]

7. For <u>4: Input Genome Release Information (1.4)</u> choose "hg19_RepeatMasker"

You can now click the blue 'Run workflow' button at top right. Results will appear in the new History and should resemble the table below for the WT cells :

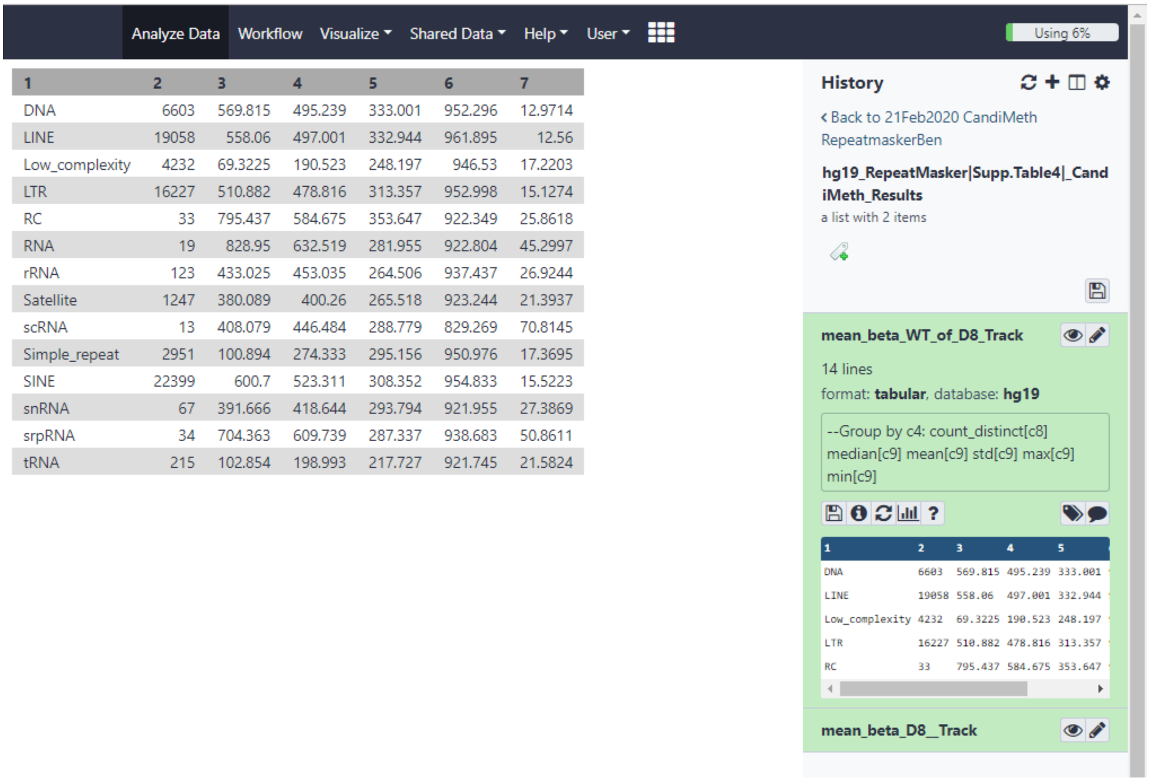

1	2	3	4	5	6	7
DNA	6603	569.815	495.239	333.001	952.296	12.9714
LINE	19058	558.06	497.001	332.944	961.895	12.56
Low_complexity	4232	69.3225	190.523	248.197	946.53	17.2203
LTR	16227	510.882	478.816	313.357	952.998	15.1274
RC	33	795.437	584.675	353.647	922.349	25.8618
RNA	19	828.95	632.519	281.955	922.804	45.2997
rRNA	123	433.025	453.035	264.506	937.437	26.9244
Satellite	1247	380.089	400.26	265.518	923.244	21.3937
scRNA	13	408.079	446.484	288.779	829.269	70.8145
Simple_repeat	2951	100.894	274.333	295.156	950.976	17.3695
SINE	22399	600.7	523.311	308.352	954.833	15.5223
snRNA	67	391.666	418.644	293.794	921.955	27.3869
srpRNA	34	704.363	609.739	287.337	938.683	50.8611
tRNA	215	102.854	198.993	217.727	921.745	21.5824

In the output Table, the different types of repetitive element as identified by the *RepeatMasker* algorithm [*www.repeatmasker.org*] are indicated, together with the number of probes etc in the same outout format as before ie

1) Name of gene, 2) Number of array probes, 3) Median methylation, 4) Standard deviation, 5) Mean methylation, 6) Maximum value and 7) Minimum value

The Names of the elements can be found on the RepeatMasker track in UCSC. As can be seen from the example Table above, a number of repeats are covered by less than 1000 probes, which may be less reliable. Names of the classes with >1000 probes on the array includes:-

- DNA — DNA repeat elements
- LINE — Long Interspersed Nuclear Elements such as LINE1
- Low_complexity — Low complexity repeats which do not fall into other categories
- LTR — LTR-containing elements such as endogenous retroviruses (ERV)
- Satellite — Satellite repeats, found near the centromeres
- Simple_repeat — Largely microsatellites, which are interspersed
- SINE — Short Nuclear Interspersed Elements such as Alu elements

Methylation varies greatly across these elements as can be seen from the minimum and maximum values, but comparisons of median methylation can nevertheless be valuable.

In Case Study 3 in the main CandiMeth paper for example, methylation in DNMT1-depleted cells (d16 in this case) can be seen to affect satellite repeats, but have little effect on microsatellites (Simple_repeat), many of which would lack any CG.

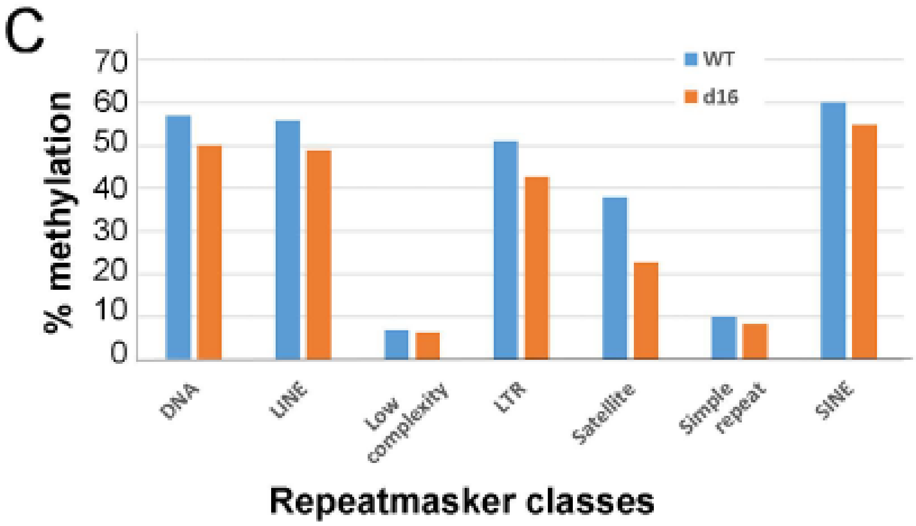

3.4 Using ChAMP-generated methylation data

While the above examples all use the data processed by the RnBeads package in R, CandiMeth can also work with data which has instead been processed using the ChAMP package. An example dataset has been provided in the Test History, Supp.Table 5. This has been uploaded as an Excel output (.csv), so it needs first to be converted into a dataset collection (See also Section 2, Step 3).

1. Convert the ChAMP csv file to a dataset collection
    a. Click on “Operations on multiple datasets” at top RHS
    b. Check the box beside Suppl. Table 5
    c. Under “For all selected” choose “Build Dataset List”
    d. Give the collection a new name e.g. “All probes ChAMP1”
    e. Once the new dataset collection appears, click on the “Operations” box again to return to normal view

2. Choosing inputs and starting the run

    a. Click on Workflows on the top ribbon and choose CandiMeth and click on the arrow

    b. Choose the option to send the results to a new History e.g. “[date] ChAMP test1”

    c. Under 1: R Package Used: (1.1) enter ‘ChAMP’

    d. For 2: Input Differential Methylation Table (1.2) choose “All Probes ChAMP1”

       [Note: This was the example name used in step 1(d) above, alter as required]

    e. At 3: Input Gene Features of Interest (1.3) choose “Supp.Table2”

    f. For 4: Input Genome Release Information (1.4) choose “hg19_all”

3. Once the workflow has finished, similar tables of Results and Tracks should appear as before (see sections 3.1.1-3.1.8) for these sample microRNA data, and all the same types of operations (looking at promoters vs gene bodies, repeat analysis, new gene queries etc) can be carried out

[Note: If your outputs from ChAMP are not normally being produced as the .csv files needed for Step 1 above, please show whoever is running the ChAMP pipeline Appendix 3 below, which contains the few lines of coding needed to do this]

4. Uploading and working with your own differential methylation data

For this you need at least one file containing information on methylation differences between two samples produced from either RnBeads or ChAMP.

4.1 Locating data files in RnBeads

1. If you received your data back as a completed Report folder with an index.html page then click on that, which should bring up a list of all reports, including differential methylation:

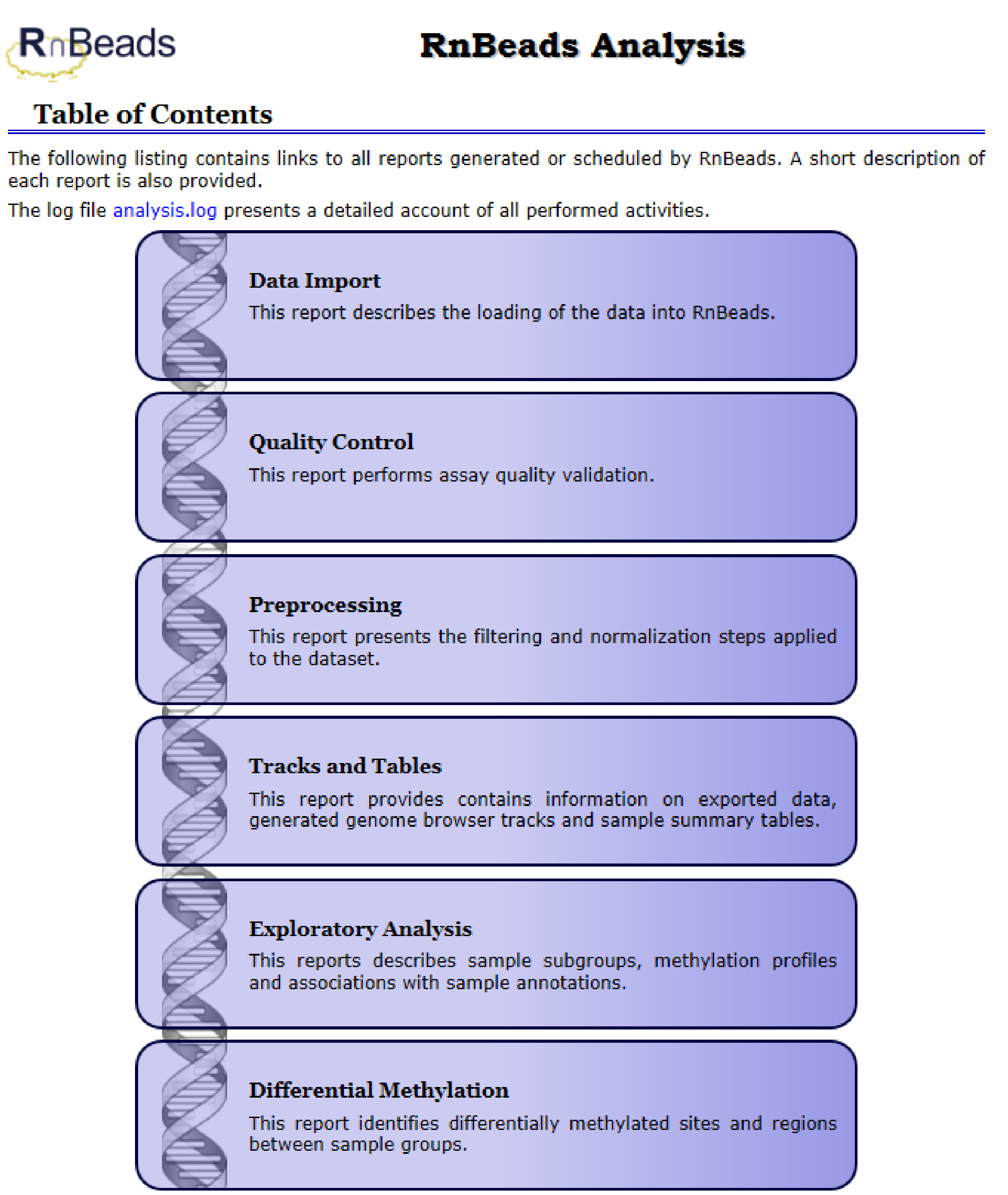

[Note: if Differential Methylation is absent then this type of analysis has not yet been done]

2. Click on the Differential Methylation report and look to see what comparisons have been done, for example:

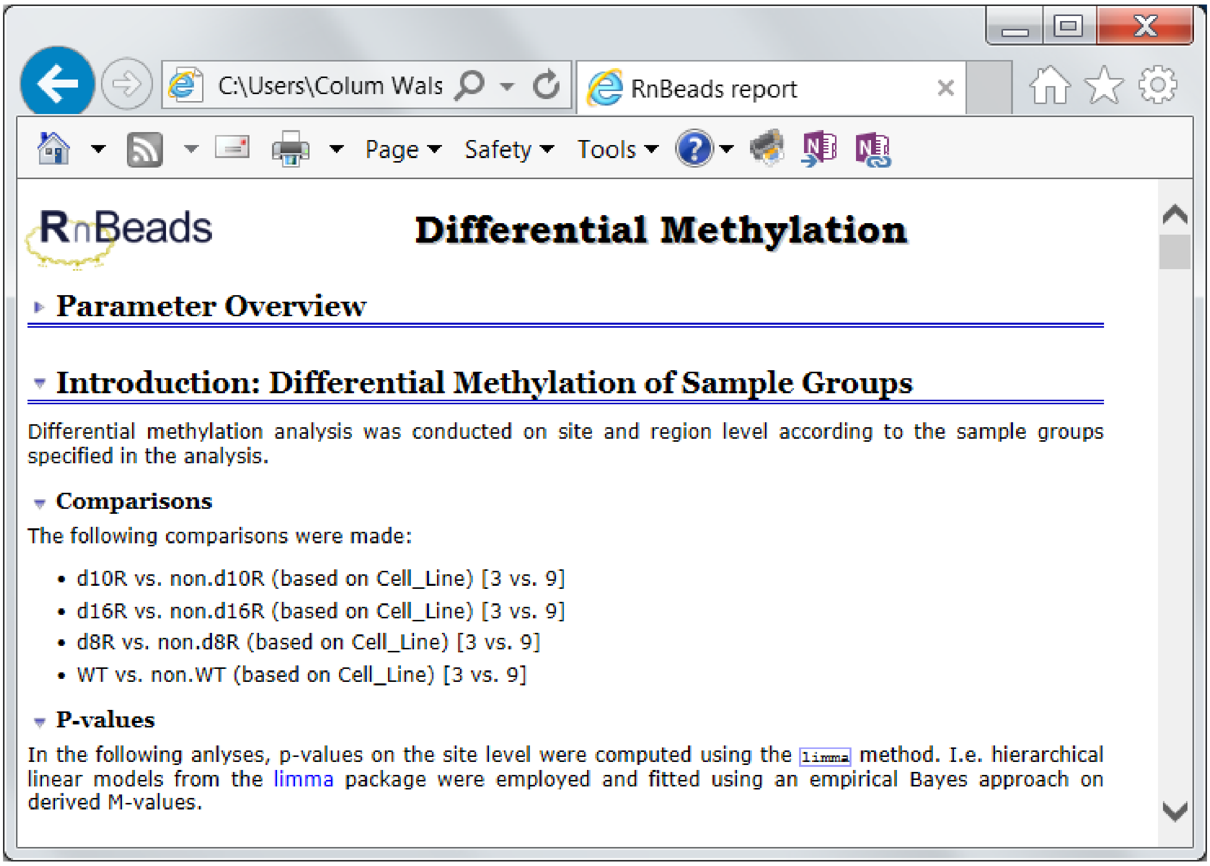

3. Each comparison listed will generate a differential methylation table labelled cmp1, cmp2 etc
4. Scroll down the page to find links to the actual differential methylation files, under the heading Differential Methylation Tables (boxed in green below):

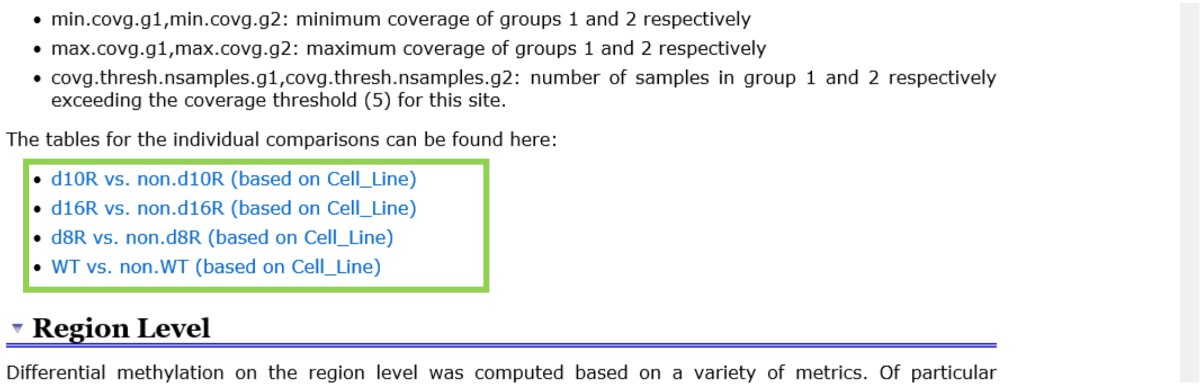

5. These hyperlinks take you the file itself- the address can be seen by holding the mouse over the link. These files are usually located in the "differential_methylation_data" folder of your Results folder and is named something similar to "diffMethTable_site_cmp1.csv" for comparison 1 etc

   [NB: the file must have data on all sites i.e. contain "_site_" to work for all types of analysis]

6. You can Open or Save As to copy the file to a new location for uploading, or use the original file in the differential_methylation_data folder

   [Tip: these are usually large files and may take some time to download/upload]

4.2 Locating data files in ChAMP

If your ChAMP related output has not been produced as a .csv file outside of R, please see the below instructions on how to write the differential methylation table to a .csv file. This code will need to be implemented in R while using the ChAMP package, so it may be appropriate to pass these on to whoever is providing bioinformatics support for the project.

-For just one comparison:

$$\mathrm{write.csv(myDMP[[x]], file = "comparison1.csv", quote = FALSE)}$$

(where x is the element number of the file comparison you wish to write to the .csv file and myDMP is the resulting object of running champ.DMP() as within the ChAMP vignette (https://www.bioconductor.org/packages/3.7/bioc/vignettes/ChAMP/inst/doc/ChAMP.html#section-differential-methylation-probes)

-For the output of multiple comparisons:

$$compnames < -names(myDMP)$$

$$for(i\ in\ 1{:}\ length(compnames))\{write.csv(myDMP[[i]], file = paste(compnames[i], \text{".csv", sep="")}, \text{quote=FALSE)}\}$$

This will create all probes differential methylation tables within your documents folder as below:

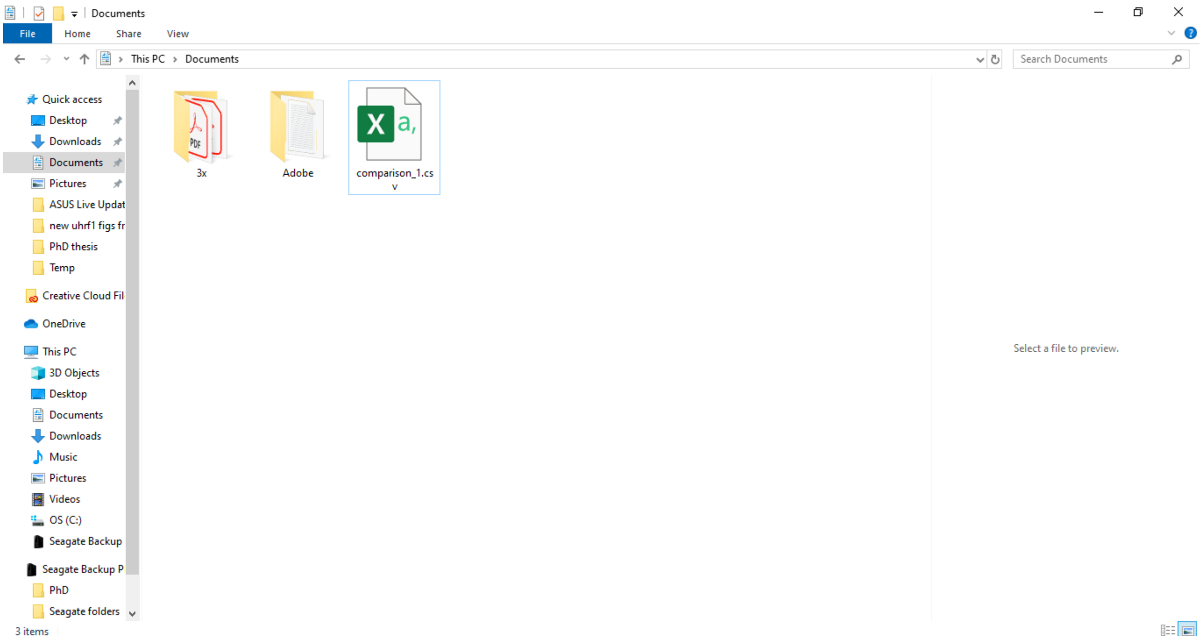

When opened, this file will look similar to the image below:

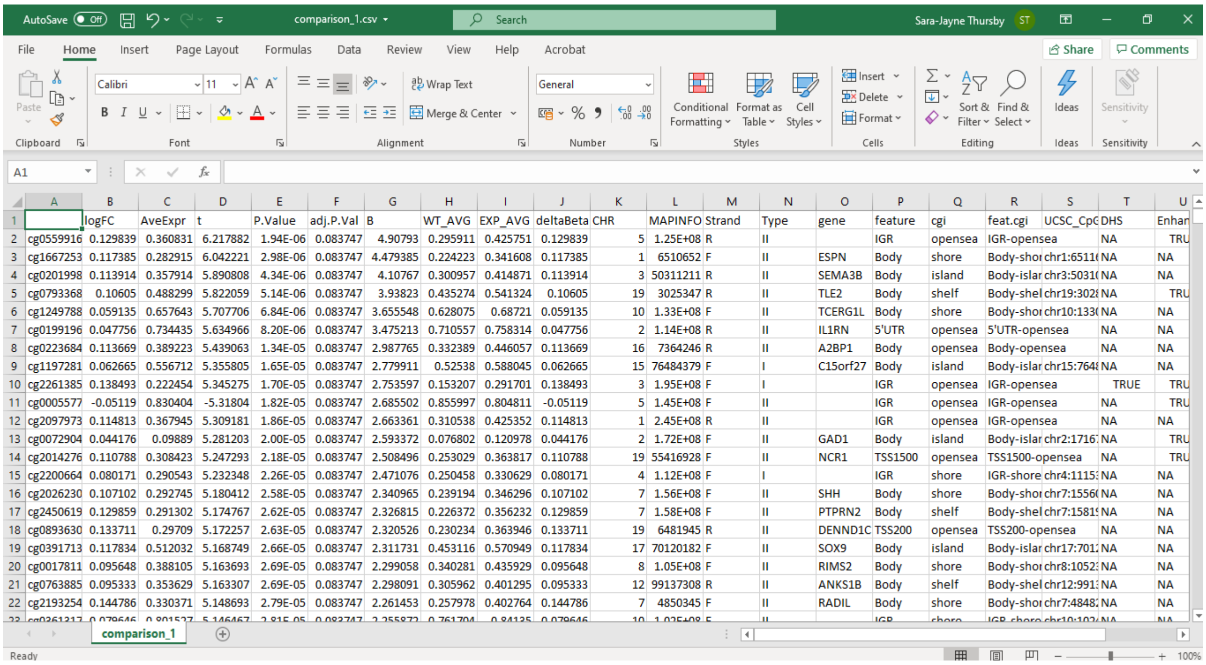

	A	B	C	D	E	F	G	H	I	J	K	L	M	N	O	P	Q	R	S	T	U
1		logFC	AveExpr	t	P.Value	adj.P.Val	B	WT_AVG	EXP_AVG	deltaBeta	CHR	MAPINFO	Strand	Type	gene	feature	cgi	feat.cgi	UCSC_CpG	DHS	Enhan
2	cg0559916	0.129839	0.360831	6.217882	1.94E-06	0.083747	4.90793	0.295911	0.425751	0.129839	5	1.25E+08	R	II		IGR	opensea	IGR-opensea		NA	TRU
3	cg1667253	0.117385	0.282915	6.042221	2.98E-06	0.083747	4.479385	0.224223	0.341608	0.117385	1	6510652	F	II	ESPN	Body	shore	Body-shor	chr1:6511	NA	NA
4	cg0201998	0.113914	0.357914	5.890808	4.34E-06	0.083747	4.10767	0.300957	0.414871	0.113914	3	50311211	R	II	SEMA3B	Body	island	Body-islar	chr3:5031	NA	NA
5	cg0793368	0.10605	0.488299	5.822059	5.14E-06	0.083747	3.93823	0.435274	0.541324	0.10605	19	3025347	R	II	TLE2	Body	shelf	Body-shel	chr19:302	NA	TRU
6	cg1249788	0.059135	0.657643	5.707706	6.84E-06	0.083747	3.655548	0.628075	0.68721	0.059135	10	1.33E+08	F	II	TCERG1L	Body	shore	Body-shor	chr10:133	NA	NA
7	cg0199196	0.047756	0.734435	5.634966	8.20E-06	0.083747	3.475213	0.710557	0.758314	0.047756	2	1.14E+08	R	II	IL1RN	5'UTR	opensea	5'UTR-opensea		NA	NA
8	cg0223684	0.113669	0.389223	5.439063	1.34E-05	0.083747	2.987765	0.332389	0.446057	0.113669	16	7364246	R	II	A2BP1	Body	opensea	Body-opensea		NA	NA
9	cg1197281	0.062665	0.556712	5.355805	1.65E-05	0.083747	2.779911	0.52538	0.588045	0.062665	15	76484379	F	I	C15orf27	Body	island	Body-islar	chr15:764	NA	NA
10	cg2261385	0.138493	0.222454	5.345275	1.70E-05	0.083747	2.753597	0.153207	0.291701	0.138493	3	1.95E+08	F	I		IGR	opensea	IGR-opensea		TRUE	TRU
11	cg0005577	-0.05119	0.830404	-5.31804	1.82E-05	0.083747	2.685502	0.855997	0.804811	-0.05119	5	1.45E+08	F	II		IGR	opensea	IGR-opensea		NA	TRU
12	cg2097973	0.114813	0.367945	5.309181	1.86E-05	0.083747	2.663361	0.310538	0.425352	0.114813	1	2.45E+08	R	II		IGR	opensea	IGR-opensea		NA	NA
13	cg0072904	0.044176	0.09889	5.281203	2.00E-05	0.083747	2.593372	0.076802	0.120978	0.044176	2	1.72E+08	F	II	GAD1	Body	island	Body-islar	chr2:1716	NA	TRU
14	cg2014276	0.110788	0.308423	5.247293	2.18E-05	0.083747	2.508496	0.253029	0.363817	0.110788	19	55416928	F	II	NCR1	TSS1500	opensea	TSS1500-opensea		NA	TRU
15	cg2200664	0.080171	0.290543	5.232348	2.26E-05	0.083747	2.471076	0.250458	0.330629	0.080171	4	1.12E+08	F	I		IGR	shore	IGR-shore	chr4:1115	NA	NA
16	cg2026230	0.107102	0.292745	5.180412	2.58E-05	0.083747	2.340965	0.239194	0.346296	0.107102	7	1.56E+08	F	II	SHH	Body	shore	Body-shor	chr7:1556	NA	NA
17	cg2450619	0.129859	0.291302	5.174767	2.62E-05	0.083747	2.326815	0.226372	0.356232	0.129859	7	1.58E+08	F	II	PTPRN2	Body	shelf	Body-shel	chr7:1581	NA	NA
18	cg0893630	0.133711	0.29709	5.172257	2.63E-05	0.083747	2.320526	0.230234	0.363946	0.133711	19	6481945	R	II	DENND1C	TSS200	opensea	TSS200-opensea		NA	NA
19	cg0391713	0.117834	0.512032	5.168749	2.66E-05	0.083747	2.311731	0.453116	0.570949	0.117834	17	70120182	F	II	SOX9	Body	island	Body-islar	chr17:701	NA	NA
20	cg0017811	0.095648	0.388105	5.163693	2.69E-05	0.083747	2.299058	0.340281	0.435929	0.095648	8	1.05E+08	F	II	RIMS2	Body	shore	Body-shor	chr8:1052	NA	NA
21	cg0763885	0.095333	0.353629	5.163307	2.69E-05	0.083747	2.298091	0.305962	0.401295	0.095333	12	99137308	R	II	ANKS1B	Body	shelf	Body-shel	chr12:991	NA	NA
22	cg2193254	0.144786	0.330371	5.148693	2.79E-05	0.083747	2.261453	0.257978	0.402764	0.144786	7	4850345	F	II	RADIL	Body	shore	Body-shor	chr7:4848	NA	NA

This file is in comma separated variable (.csv) format and can now be uploaded to Galaxy to be used in CandiMeth, as detailed in the next section and in section 3.2 above.

4.3 Uploading your data files to Galaxy

This is essentially as in section 3.2 above, where you uploaded a new list of gene names. In brief,

1. Navigate back to the History containing the Test data
2. Using the Tools window (LHS) choose >Get Data > Upload File > Choose Local File and locate the methylation data you wish to upload (e.g. diffMethTable_site_cmp1.csv). Alternatively, you can drag and drop it in.
3. Set “Type (set all)” to whatever kind of file you are uploading, for example, RnBeads based outputs are usually comma separated variable files (.csv)

   [Tip: The default “Auto-detect” setting works well for most file formats]

4. Set “Genome (set all)” to either Human Feb. 2009 (GRCh37/hg19) or Human Dec. 2013 (GRCh38/hg38) depending on what genome your array was mapped to.

   [Tip: You can type in Human here to bring up all the human genomes and save time]

   The window should look something like this:-

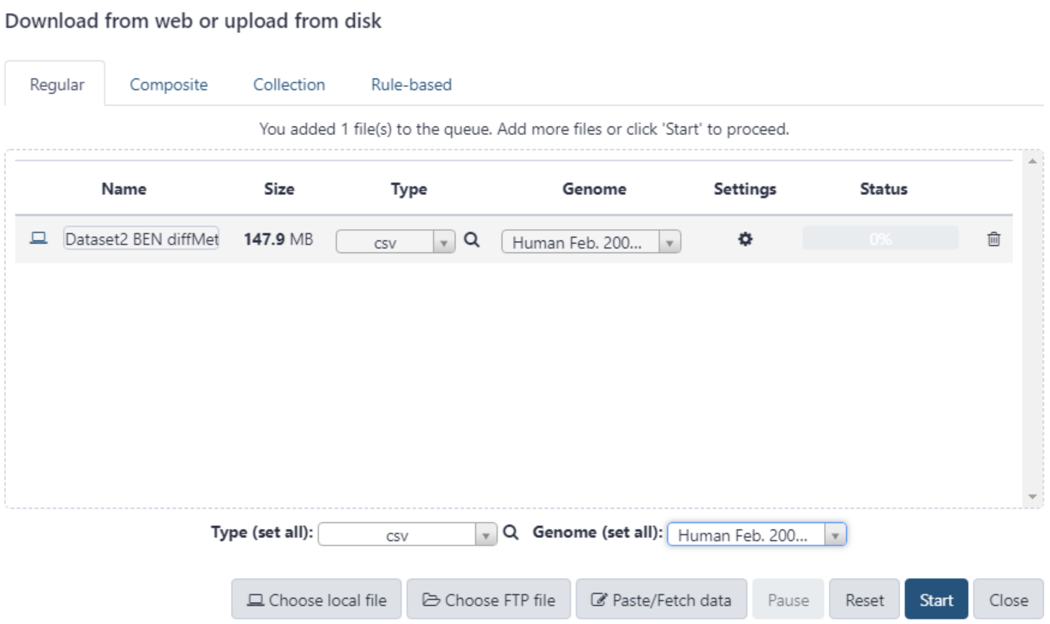

5. Click “Start”: the file should upload to Galaxy and the upload window will go green and a tick appear under “Status”- this can now be closed
6. In the main window, the new data will appear as a dataset on the RHS: this goes from grey to orange then finally green if all is well
7. NB: the input Differential Methylation Table still has to be converted from a table into the form of a Dataset Collection as before (see Section 2 above)
8. Give the collection a more specific name e.g. “Dataset2 [your name]” and click “Create”
9. A new entry should appear in the RHS with the new name and “a list with 1 (or more) items”-this is the Dataset Collection and is now ready to be processed by CandiMeth

Appendix 1. Primer for those unfamiliar with the UCSC genome browser

As a result of major efforts from many scientists around the world, the complete genetic code present in our DNA (our genome) has been characterised and mapped. As we have ~4 billion individual "bits" or DNA bases, split into 23 chromosomes, one of the main problems that arose was how to find the information for any specific gene, and relate it to its surrounding genes and other information we might have such as whether the gene was associated with specific diseases. In answer to these problems, a new way of looking at genetic information called a genome browser was devised, which works much like a web browser, but specific to our genes and information associated with them. One of the most popular such browsers is the one devised by the University of California at Santa Cruz (UCSC), called for short the UCSC genome browser. The UCSC browser can be used to look at information from other species too, such as mouse and many others: a link to human genome browser version can be found here: https://genome-euro.ucsc.edu/cgi-bin/hgGateway?redirect=manual&source=genome.ucsc.edu and should look like:

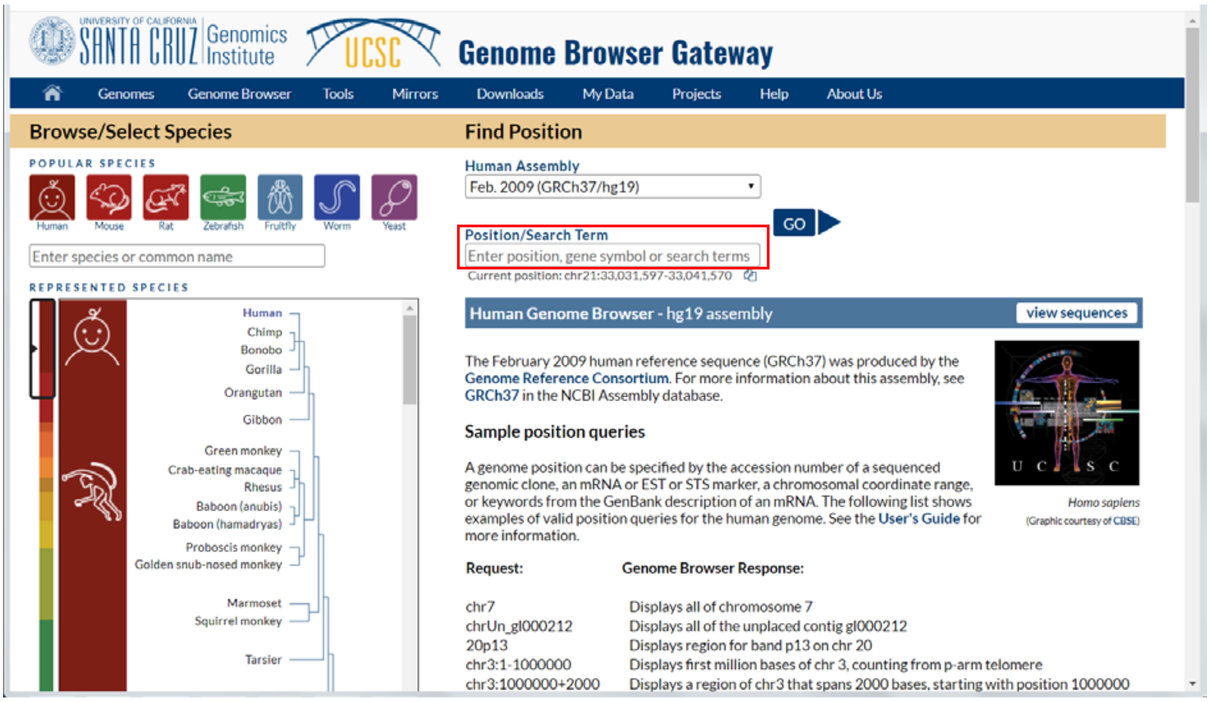

A simple way to think of the UCSC genome browser is to think of it as basically a zoomable map, not unlike Google maps. It shows us a representation of chromosomes in our cells, with the positions of the different genes indicated on each. We can take a virtual tour of our genome by scanning around, or zoom in to look at specific regions in greater detail. Most usefully, we can search for a genetic "address" and the browser will locate it for us and take us to a close-up view of the gene and its surroundings.

To get started, write in the name of a gene in the "Position/Search term" box (outlined in red above). Like many apps, the browser tries to guess your destination, so start by writing the name for the human hemoglobin gene "Hbb" in the box: the "best guess" will appear under the box, choose that using the mouse so that it is highlighted and appears in the box (right) and click the big blue "Go" button.

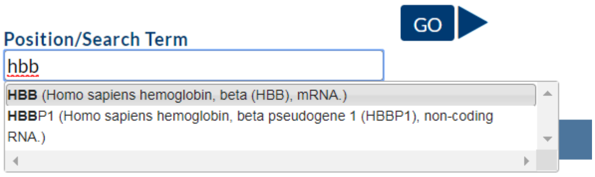

This will open a new view in the browser which may be quite crowded with information, like a busy map. This is because, like the maps we use to navigate in everyday life where we can find the nearest coffee shop or petrol station, new interesting information and locations are constantly being added to the genome map, a bit like a news feed, and this can make it quite busy.

To get a simple map with just the Hemoglobin gene, do the following:

- Click on >Hide all (red box below)
- Under UCSC genes, choose “dense” (yellow box below)

The view should now look something like this:

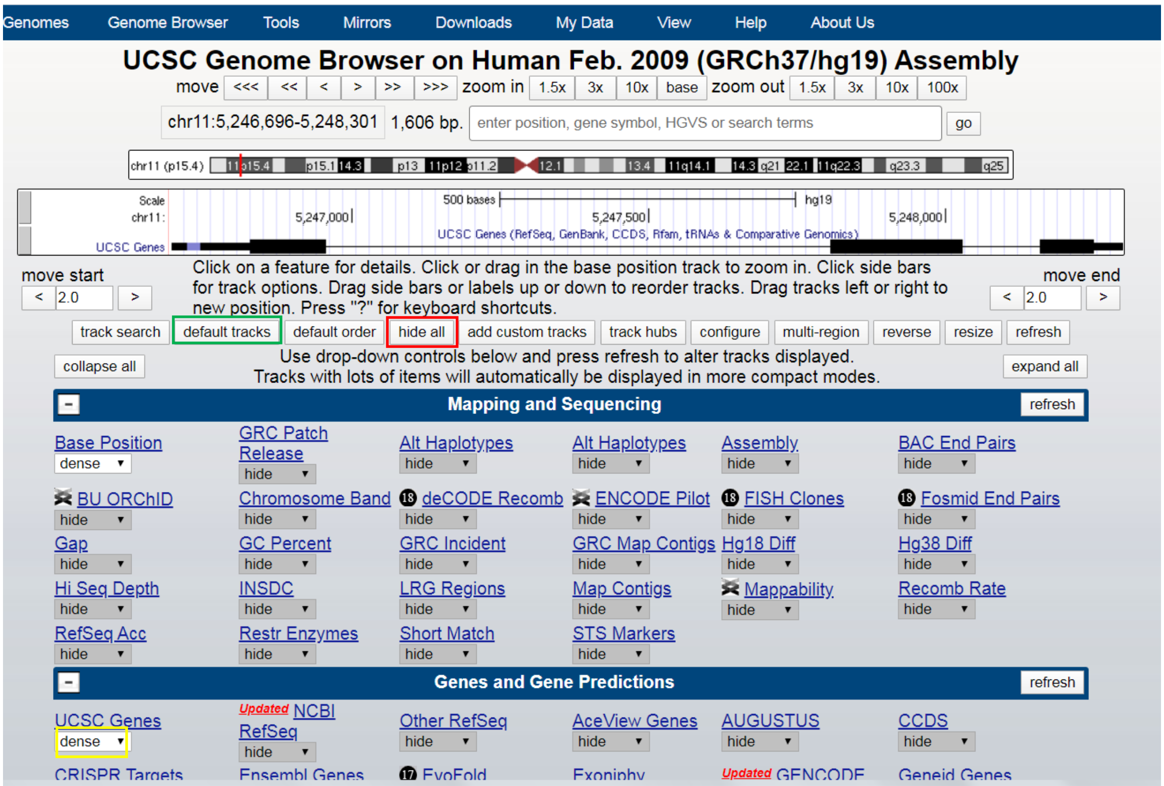

- Here we see the Hemoglobin gene (labelled UCSC gene) outlined on the map as black boxes joined by thin lines: these are the exons (boxes) and introns (lines)
- Above there is a little picture or ideogram of human chromosome 11, showing where the gene is located (red line at left)

There is a wealth of other data available for each gene, each available through the pull-down menus shown. Each generates a new line or “track” on the map below the gene, showing the new information in parallel. To get a flavour of this, the user can try “default tracks” (green box above) which will show the same gene, but with information below it from every major group (blue header):

- UCSC_Genes — The default map, best current estimate of the start and end of the gene
- NCBI_RefSeq — Gene start and end as defined conservatively, based on curated data
- Publications — Scientific papers with links to this gene
- GTEx gene — Data on where the gene is thought to be expressed
- ENCODE — Clues as to how the gene may be regulated based on epigenetic info
- Conservation — Showing which regions of the gene may be conserved in vertebrates
- dbSNP_153 — Known variations in the DNA sequence at this gene

These lines of information are fully clickable in the top window: right-clicking or double-clicking can bring you to pages with further information on each track.

We can also add our own data to the genome maps using our own tracks, which is what we do using CandiMeth (see section 3.1.6ff in main Guide). More details on how to export data, views of the maps and more can also be found there.

Like political maps, the human genome map is constantly being updated with the latest borders and newest information. These updates are known as genome builds or genome assemblies, an example is the GRCh37/hg19 (Genome Release Consortium human build 37) genome build used in section 3.1.

Further documentation on the UCSC genome browser can be found here:
https://genome.ucsc.edu/goldenPath/help/hgTracksHelp.html

-Happy browsing!

Appendix 2. Quick guide to the Galaxy web environment

Galaxy is a free online environment for user friendly data science. It can be located here, https://usegalaxy.org/ and requires users to create an account and log-in to utilise the service (registration for this service can be found here, https://usegalaxy.org/root/login?is_logout_redirect=true). Once logged in the home page should look something similar to the below image:

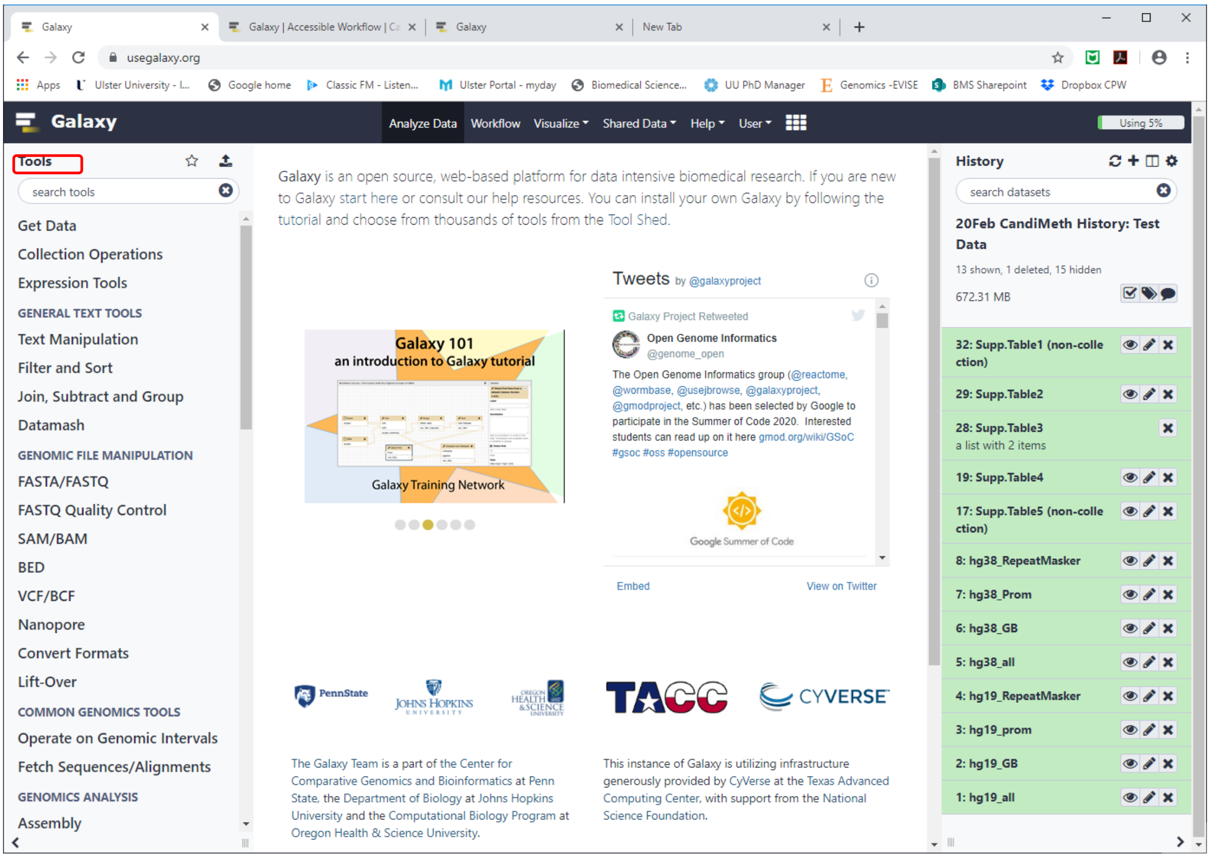

Here, tools can be found in the tools panel on the left-hand side and any process that the user has executed can be found in the history column in the right-hand side. Every process a user executes creates a new item in the history column. Multiple histories can be created via the gear icon and multiple histories can be viewed using the book-like icon. Data can be uploaded to the Galaxy interface, as detailed in section 4.3 and using the "Get data" function (red box above) the user can load data from publicly available external sources such as UCSC genome browser or NCBI.

Further detail regarding the Galaxy interface can be found here (https://galaxyproject.org/tutorials/g101/) or in the Galaxy Training Network (https://training.galaxyproject.org/).

Appendix 3. Importing CandiMeth to a custom Galaxy instance

1. To download and upload CandiMeth to an alternative Galaxy instance, navigate to the workflow tab using the “workflow” tab at the top of the Galaxy window.

2. Click on the import button (circled in yellow here) at the top right of the window

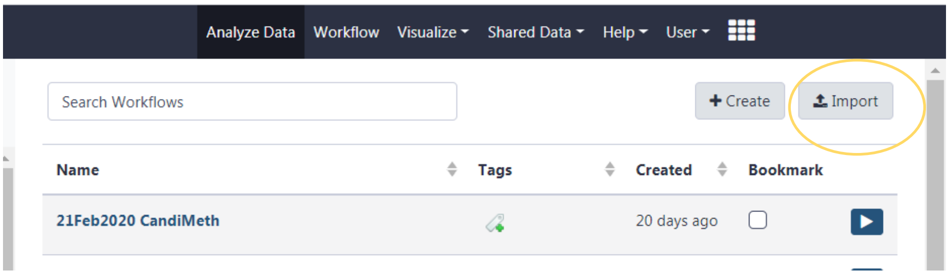

3. Paste this link https://usegalaxy.org/u/sarajayne-thursby/w/candimeth-8/json

   into the “Archived Workflow URL” section of the import workflow screen and click on import workflow, as below

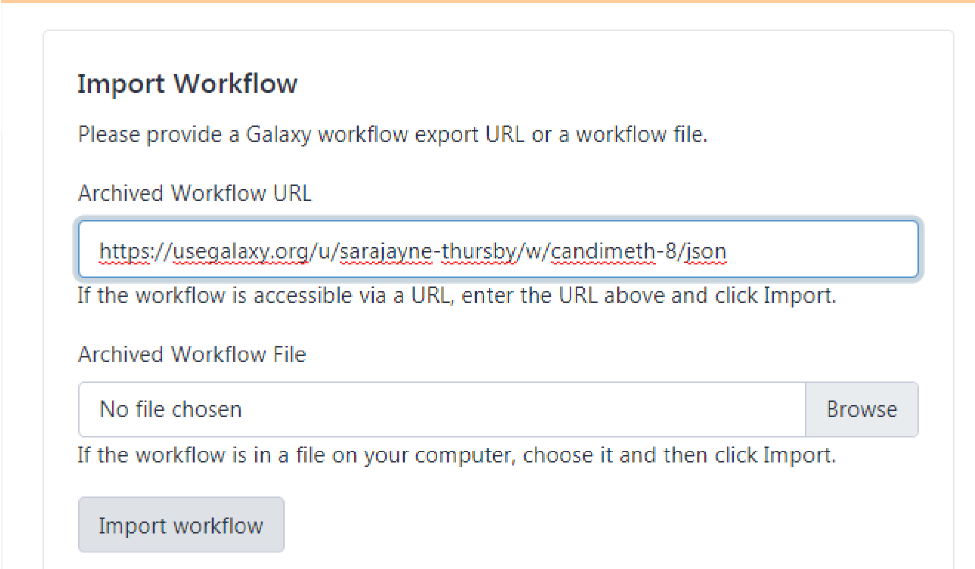

4. CandiMeth should then show up in the list of workflows available to you, as below.

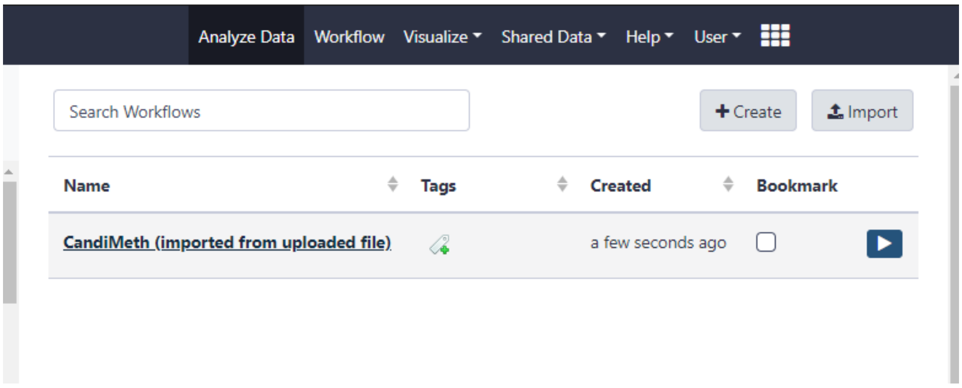

If any difficulties persist in this or other procedures, please contact the authors.
