# Supplementary material for: CandiMeth: Powerful yet simple visualization and quantification of DNA methylation at candidate genes
Source: Gigascience. 2020 Jun 22;9(6):giaa066. doi: 10.1093/gigascience/giaa066 (PMC7307318; doi:10.1093/gigascience/giaa066)
Supplement: giaa066_Response_to_Reviewer_Comments_Original_Submission [file giaa066_response_to_reviewer_comments_original_submission.pdf]

Author's Response To Reviewer Comments

Close

GIGA-D-19-00417
Response to Editorial and Reviewers' comments

CandiMeth: Powerful yet simple visualization and quantification of DNA methylation at candidate genes
Sara-Jayne Thursby; Darin K Lobo; Kristina Pentieva; Shu-Dong Zhang, Ph.D.; Rachelle E Irwin; Colum P Walsh, Ph.D.

Dear Professor Zhou,

Many thanks for your letter with an interim decision on our MS above and we appreciate the kind words and positive feedback from the reviewers and yourself. This is particularly the case given that they experienced problems with the workflow, which we had hoped to avoid through having it tested by several users prior to submission. The difficulty was due in large part to the withdrawal of a tool from Galaxy without notice, as well as a time-dependent decay of the converted dataset collections. We have addressed both these issues, tested extensively, and revised the manuscript [revisions in blue]. Our detailed responses to the comments, which we have numbered for ease of reference, are given below.

Editor's comments:

1. .. please register any new software application in the bio.tools and SciCrunch.org databases to receive RRID (Research Resource Identification Initiative ID) and biotoolsID identifiers

The CandiMeth RRID: SCR_017974 and Biotools identifier (Biotools:CandiMeth) have been added to the abstract and at the start of Methods on p5

2. Please also ensure that your revised manuscript conforms to the journal style, which can be found in the Instructions for Authors on the journal homepage.

We have done our best to match journal style as indicated on the homepage

3. If the data and code has been modified in the revision process please be sure to update the public versions of this too.

These have been updated to match the latest version

Reviewer #1:
It's been a delight reading your manuscript. I agree that publishing such workflows that ultimately serve as a tool is worth considering. This paper describes so concisely the method used on CandiMeth that even without deep knowledge on the subject it is easy to understand.
We really appreciate the positive comments here and have made every effort to address the suggestions made, as indicated below
Specific Comments:
1. While reading p4, on line 6 I missed a reference to indicate the comparison of inter-sample reproducibility

Thank you for highlighting this: we have inserted the reference we were thinking of, showing high inter-sample reproducibility using the array (Bibikova et al Genomics 98:288 2011), and rephrased the sentence to make our meaning clearer:
.."where a lower CpG resolution is satisfactory, but where greater inter-sample reproducibility is required [16]." [p4, L6]

2. On Fig 2, is it normal that bars exceed the marked limits on tracks and overlap?

This issue was due to some inadvertent editing of the image before submission: a new version has been generated using CandiMeth and, as can be seen, the default spacing between the tracks in the UCSC browser is sufficient and there is no overlap

3. p12, ChAMP output is Supp Table 5, I suggest including this information in the GitHub Guide.

For this resubmission, we have written a much more extensive step-by-step User Guide which is available through the GitHub page (section A there) and is attached as a file with this resubmission. This more complete User Guide has specific sections on using ChAMP data (sections 3.4 and 4.2). We have retained a Quick Start guide on the GitHub page for more experienced users in Markdown format (section G) and this also flags more clearly that ChAMP outputs can be used in addition to RnBeads.

"At least one file containing information on methylation differences between two samples produced from either RnBeads or ChAMP (for Input Type 1 below)" [GitHub, section G, L5]

4. Abbreviations, include KD since it's repeatedly used in the figures.

Done

5. GitHub and Workflow.

The website is very well designed, and docs are easy to follow and very detailed. I followed these instructions to test it but the workflow didn't get to run. My new histories are empty. I also tried to copy the files into a new history and run the workflow from there, with same result. I got an error file from Galaxy, which I'll try to attach to this report.

This was very unfortunate and we apologize for the inconvenience. Several of us had tested the workflow and histories before submission, when everything worked well. We have traced the problem to two sources: converting the R output files into a dataset collection for input and the unnotified removal of tools from Galaxy.

To maximize ease of use for the bioinformatics novices, we wanted to have all the datasets ready to use and so had converted the input differentially methylated probes table from R into a dataset collection in the History. However unfortunately these appear to be temporary files and decay over time. We have therefore not done any conversions this time and instead have moved the instructions in the guide on how to do that to earlier in the procedure (now step 5 in the Quick Start guide and Section 2,Getting Set Up step 3 in the complete User Guide), so that the user does the conversion themselves, which solves that issue.

6. Next I downloaded the history and workflow locally to test on my local Galaxy in Docker. Two tools weren't installed and I had to find on the Galaxy Tool Shed, finding their names on the original galaxy.org server (the names on the paper or workflow are modified).

This was the second problem: the Galaxy team had removed the Cut and Join tools from the newer version of Galaxy between submission of the MS and review, so the workflow would not work. We have removed these tools and recoded the relevant parts in AWK (Steps 6.1 and 6.2 in the new version of Fig.4). The relevant text has been added or changed in the Figure legend and main MS (pp14 & 15) as well. Table 1 has also been updated with the new tool names, and we have added a column indicating what parts of the workflow use each tool.

In addition to the changes above, we had to recode the original import of data in SED due to withdrawal of another tool in the interim, this has also been updated in the table, Fig.4 (new step 2.1) and relevant text on p14.

7. When importing the history, the dataset lists need to be re-created manually, so I did it with Supp Table 1.

See response to point 6 above

8. When running the workflow it got stuck again, this time I realized that a box called variable was not linked, and removing it the workflow would run but not create outputs due to this missing input.

This was related to the tool issue and is no longer a problem with the updated workflow

9. I run Galaxy 18.04, while the workflow requires Galaxy 19 features

Unfortunately we only have access to Galaxy 19 features as we do not have a private instance: however we wanted the workflow to be accessible by users without access to their own instance so have

concentrated on that version. The Tools in Table 1 can be used to downgrade the workflow to function with Galaxy 18.04 if so desired however.
We have also provided a link to a downloadable version which should import and work on other Galaxy instances- see points 12 and 13 below and Appendix 3 of the User Guide.
10. Indicate minimum requirements to the workflow

If the user is working through the on-line version of Galaxy (usegalaxy.org) as is the intention, this is quite platform-independent. In order to understand and potentially alter the workflow they would need to know what tools are used where in the workflow, and this is indicated in the updated Table 1. For their own instance of Galaxy, we have included a link to download and import the workflow (Appendix 3 of User Guide) and a .yaml file detailing the technical requirements. We now refer to this under Overview on p8 of the revised MS:

"CandiMeth is optimised to work on the latest version of Galaxy (19.0) through the Galaxy website (www.usegalaxy.org), thus making it platform-independent. For users who have their own instance of Galaxy, the workflow can be downloaded and imported via a link on the GitHub page above, where a .yaml file is also available."

11. Functional workflow for a lower Galaxy version is suggested but not required.

See response to point 10 above and to next point

12. Usage of this workflow only for users of galaxy.org misses out a large proportion of researches. I suggest inclusion of instructions to download and upload the data and history to a custom Galaxy instance, if not already present.

We have now included information in the User Guide and a link to allow users to download the workflow and import it into their custom Galaxy instance (User Guide Appendix 3)
This is mentioned in the main MS text (p8) now as well.

13. Include a .yaml file listing the packages used by the workflow as specified by Galaxy

This has been created and uploaded with the revised MS and mentioned in the text (p8)

14. I'd like to be able to use the workflow, please check if there's a bug or why it isn't working for me

We're pleased the reviewer wishes to use CandiMeth going forward: the new version should be clearer and more robust, and was working for multiple users at time of resubmission

15. A typo on the docks: on part C, candimeth outputs, third line, "you wish to view"

Done

Reviewer #2:
In general I like this work, it focused on a very straightforward but common required demand: Mapping differential methylated status to whole genome, and match track with other genomic results. It's a good attempt to integrating traditional R package, cloud computing resource, and UCSC browser. I hope this pipeline is robust enough among these various tools, and hopefully the author in the future would not be troubled too much for constantly upgrading of any of these gears.

We thank the reviewer for their positive and constructive comments and are pleased that they also feel it meets a common demand. The concern regarding tools is justified given the withdrawal of two tools between submission and review: we have recoded parts of the pipeline as indicated, and will be monitoring the workflow on a weekly basis, as we have a number of frequent users in our own labs.

We have also given more comprehensive guidance now as part of a new User Guide, an extensive document taking users step-by-step through tutorials and indicating how to upload and convert data as well as many other functions. There is also a Quick Start Guide as section G on the GitHub page for those familiar with the program and who want a quick reference guide only.

It does highlight a need for Galaxy to be more transparent and less cavalier in their treatment of Tools,

as we have experienced this problem once before during workflow development: we have written to those maintaining the Galaxy resource and hopefully this will help ensure changes are flagged in time to allow smooth transitions to newer CandiMeth versions to adapt to new tools.

Specific Comments:

1. I run the default CandiMeth history, with RnBeads (Supp.Table 1), but the "results table" for region statistic are always empty with 0 rows. The tracks are generated successfully, but seems the results tables are not. I just followed the Step-by-Step guild on google drive, not sure if I missed anything or the guild should be improved

This would have been due to the problems which arose between submission (tested and working) and review involving 1) dataset conversion and 2) Tool replacement. These have been detailed above (see responses 6 & 7 to Reviewer 1), and are now corrected and the new version extensively tested. In case our responses to reviewer 1 above are not visible to you, in brief: 1) we had converted the R output files into dataset collections in the history to try and make it more user-friendly, but the collections were not as stable when done like this- users must now convert the example data themselves, a simple step we have now detailed as step 5 in the Quick Start guide on GitHub and Section 2, Getting Set Up step 3 in the complete User Guide document; 2)some tools (Join, Cut) were withdrawn by the Galaxy team between submission and revision; we have recoded these parts and updated Fig4, Table 1 and the text to reflect these changes.

2. The ChAMP Demo is not working, without any data generated. I used default "Supp.Table 5" for test. The error is: "Input dataset 'Supp.Table5' was deleted before the job started"...

This would reflect the same problem with the converted dataset: if the user does the conversion themselves then this will not be a problem: as a visual reminder the Supp.Table5 dataset says underneath "(unconverted)". See also step 5 in the Quick Start guide on GitHub

"The input Differential Methylation Table has to be converted from a table into the form of a Dataset Collection: This is in case there are multiple differential methylation tables to be assessed, then CandiMeth can assess them all simultaneously and present them in the typical Results and Tracks outputs, as opposed to multiple outputs that might make your history very crowded: - Click on the already checked box at the top of the History panel (mouse over shows "Operations on multiple datasets"): this will cause checkboxes to appear beside all of your datasets as well as some choices to appear at top - Check the box beside the Differential Methylation Table dataset(s) - Under the pulldown menu beside "For all selected" choose "Build Dataset List" - In the window that appears, you can give the collection a new name e.g. "DMP set1" and click "Create" - A new entry will appear in the RHS with the new name and "a list with 1 (or more) items"- this is the Dataset Collection and is now ready to be processed by CandiMeth Upload Input Type 2: Candidate Features of interest"

There is also a more detailed guidance on this with screenshots etc as part of the more extensive User Guide (Section 2, Getting Set Up step 3). If these steps are followed, then the workflow will process the data without any errors.

3. Minfi is also an important package, and I think it generated DMP tables as well, however, the paper did not mention (or cite) minfi at all, nor the pipeline. Is that because minfi's result is similar to ChAMP or RnBeads or some other reasons?

Minfi is not an end-to-end pipeline per se, but rather an individual workpackage which has to be run using more bespoke coding in R: however it is called as part of the RnBeads and/or ChAMP pipelines, with the outputs then further handled and integrated into the html outputs by these two more user-friendly pipelines, which only require a few lines of code to run. As we are aiming primarily at users of RnBeads and ChAMP, the Minfi DMP outputs will be presented at the end of these pipelines as RnBeads or ChAMP DMP tables, and so are catered for in this way.

Minor suggestions:
4. CandiMeth provides some nice features like BLAT Primer Designing, Repeats Analysis, which are not mentioned in the end part of introduction. Some researchers (like me) would prefer to find key features like this on that part, so maybe it's a good idea to include them. I discovered these features only at the later section of paper

Two sentences have been added to the end of the Introduction to highlight these features:
“This also facilitates the design of assays to cover specific CGs using Blat.2 (p4)….. It also has a bespoke analysis allowing estimation of methylation differences at repetitive sequences by leveraging the RepeatMasker tracks at UCSC.” (top p5)

5. I would prefer to put Step-by-Step guild in Github repo as well in Markdown format, instead of a PDF on google drive...

As mentioned above, we have now written a much more extensive step-by-step User Guide (attached to resubmission and available as download from GitHub site, rather than Google Drive) which provides comprehensive instruction in how to use CandiMeth, with screenshots and complete tutorials. This has been found very useful and easy to follow by our team of beta- testers in-house. As this would be far too long to code in Markdown, we have instead put a brief Quick Start Guide for the experienced user in Markdown as section G of the GitHub page.

6. As far as I know, ChAMP does not provide csv download for DMP table, so I think it worth add one section in guild for data converting from those R package. It may only cost 1-2 lines of R code but still worth being mentioned.

We’d like to thank the reviewer for highlighting this: some lines of R code have been added to the Guide to allow users to convert their ChAMP outputs into csv file format (User Guide section 4.2 Locating data files in ChAMP):

“-If your ChAMP related output has not been produced as a .csv file outside of R, please see the below instructions on how to write your differential methylation table to a .csv file:

-For just one comparison:

```
write.csv(myDMP[[x]],file=”comparison1.csv”,quote=FALSE)
```

(where x is the element number of the file comparison you wish to write to the .csv file and myDMP is the resulting object of running champ.DMP() as within the ChAMP vignette (https://www.bioconductor.org/packages/3.7/bioc/vignettes/ChAMP/inst/doc/ChAMP.html#section-differential-methylation-probes)

-For the output of multiple comparisons:

```
compnames<-names(myDMP)
for(i in1:length(compnames)){write.csv(myDMP[[i]],file=paste(compnames[i],
"\".csv\",sep=\"\"),quote=FALSE)}"
```

This will create all probes differential methylation tables within your documents folder”

Further guidance and screenshots are shown in the User Guide

7. In many paper, "DMP table" means CpG probs only show significant differentiation between phenotypes, like P value <= 0.05 .eg. However, the "DMP Table" used in CandiMeth is actually all Probe's differential analysis result (including non-significant ones), without any cutoff selection. I think it should be mentioned in paper, as many tools would automatically return only significant probes.

We were careful to refer to the tables as containing differentially methylated probes or regions , not significantly differentially methylated probes/regions, but we have added some text to highlight this distinction more [under Example outputs, p10].
“Note that this track shows all differences in methylation, however small: the FDR-corrected probes are shown in the next track.”

The generation of a separate track only showing the FDR-significant probes in the outputs also should highlight this difference we hope. CandiMeth therefore generates three types of tracks, all of which we and our collaborators have found to be useful:- 1)absolute methylation level tracks, one per sample (raw β- all probes), 2)differential methylation ($\Delta\beta$) plotting differences in methylation between pairs of tracks and 3) tracks showing only the probes that are significant at FDR <0.05 from the comparisons. These designations have been made clearer in the example outputs section on p10.

We wanted to generate tracks showing all probes, as well as FDR-significant only probes, since in our experience we had found that many smaller sample sets had no probes with FDR<0.05. The comparison between the two types of track is also valuable for example if trying to establish if there are any probes in a region at all. The use of all differentially methylated probes rather than just FDR-corrected allowed us to detect the gain in methylation in the PCDHG exons shown in Case Study 4 here: our previous analysis of the PCDH loci (O'Neill et al Epigenetics & Chromatin 2018) had missed this due to only using the FDR-corrected probes.

8. "RnBeads" and "Rnbeads" can both be seen in paper, is that a typo? The same for "ChAMP" and "Champ".

Yes, apologies for these typos: we have gone back carefully over the text and ensured all instances match "RnBeads" and "ChAMP" (however the workflow accepts all variants!).

Close
