# Supplementary material for: CandiMeth: Powerful yet simple visualization and quantification of DNA methylation at candidate genes
Source: Gigascience. 2020 Jun 22;9(6):giaa066. doi: 10.1093/gigascience/giaa066 (PMC7307318; doi:10.1093/gigascience/giaa066)
Supplement: giaa066_Reviewer_2_Report_Original_Submission — Yuan Tian -- 12/30/2019 Reviewed [file giaa066_reviewer_2_report_original_submission.pdf]

Reviewer Report

Title: CandiMeth: Powerful yet simple visualization and quantification of DNA methylation at candidate genes

Version: Original Submission **Date:** 12/30/2019

Reviewer name: Yuan Tian

Reviewer Comments to Author:

In general I like this work, it focused on a very straightforward but common required demand: Mapping differential methylated status to whole genome, and match track with other genomic results. It's a good attempt to integrating traditional R package, cloud computing resource, and UCSC browser. I hope this pipeline is robust enough among these various tools, and hopefully the author in the future would not be troubled too much for constantly upgrading of any of these gears.
However, I get some problems when I was running the demo:
Key questions:
1: I run the default CandiMeth history, with RnBeads (Supp.Table 1), but the "results table" for region statistic are always empty with 0 rows. The tracks are generated successfully, but seems the results tables are not. I just followed the Step-by-Step guild on google drive, not sure if I missed anything or the guild should be improved.
2: The ChAMP Demo is not working, without any data generated. I used default "Supp.Table 5" for test. The error is: "Input dataset 'Supp.Table5' was deleted before the job started"...
3: Minfi is also an important package, and I think it generated DMP tables as well, however, the paper did not mention (or cite) minfi at all, nor the pipeline. Is that because minfi's result is similar to ChAMP or RnBeads or some other reasons?
Some minor suggestions:
1. CandiMeth provides some nice features like BLAT Primer Designing, Repeats Analysis, which are not mentioned in the end part of introduction. Some researchers (like me) would prefer to find key features like this on that part, so maybe it's a good idea to include them. I discovered these features only at the later section of paper.
2. I would prefer to put Step-by-Step guild in Github repo as well in Markdown format, instead of a PDF on google drive...
3: As far as I know, ChAMP does not provide csv download for DMP table, so I think it worth add one section in guild for data converting from those R package. It may only cost 1-2 lines of R code but still worth being mentioned.
4: In many paper, "DMP table" means CpG probs only show significant differentiation between phenotypes, like P value <= 0.05 .eg. However, the "DMP Table" used in CandiMeth is actually all Probe's differential analysis result (including non-significant ones), without any cutoff selection. I think it should be mentioned in paper, as many tools would automatically return only significant probes.
5: "RnBeads" and "Rnbeads" can both be seen in paper, is that a typo? The same for "ChAMP" and

"Champ".

Level of Interest

Please indicate how interesting you found the manuscript: Choose an item.

Quality of Written English

Please indicate the quality of language in the manuscript: Choose an item.

Declaration of Competing Interests

Please complete a declaration of competing interests, considering the following questions:

- Have you in the past five years received reimbursements, fees, funding, or salary from an organisation that may in any way gain or lose financially from the publication of this manuscript, either now or in the future?
- Do you hold any stocks or shares in an organisation that may in any way gain or lose financially from the publication of this manuscript, either now or in the future?
- Do you hold or are you currently applying for any patents relating to the content of the manuscript?
- Have you received reimbursements, fees, funding, or salary from an organization that holds or has applied for patents relating to the content of the manuscript?
- Do you have any other financial competing interests?
- Do you have any non-financial competing interests in relation to this paper?

If you can answer no to all of the above, write 'I declare that I have no competing interests' below. If your reply is yes to any, please give details below.

I declare that I have no competing interests.

I agree to the open peer review policy of the journal. I understand that my name will be included on my report to the authors and, if the manuscript is accepted for publication, my named report including any attachments I upload will be posted on the website along with the authors' responses. I agree for my report to be made available under an Open Access Creative Commons CC-BY license (http://creativecommons.org/licenses/by/4.0/). I understand that any comments which I do not wish to be included in my named report can be included as confidential comments to the editors, which will not be published.

Choose an item.

To further support our reviewers, we have joined with Publons, where you can gain additional credit to further highlight your hard work (see: https://publons.com/journal/530/gigascience). On publication of this paper, your review will be automatically added to Publons, you can then choose whether or not to claim your Publons credit. I understand this statement.

Yes Choose an item.
